# The miR-744-5p/connexin 43 axis modulates *Salmonella*-containing vacuole association with the Golgi network

**DOI:** 10.1038/s44318-026-00929-3

**Published:** 2026-10-10

**Authors:** Jane Dias, Caio Haddad Franco, Laura Maria Alcantara, Susana Costa, Miguel Mano, Ana Eulalio

**Affiliations:** 1 RNA & Infection Laboratory, Center for Neuroscience and Cell Biology (CNC-UC), Centre for Innovative Biomedicine and Biotechnology (CIBB), University of Coimbra, Coimbra, Portugal; 2 Functional Genomics and RNA-based Therapeutics Laboratory, Center for Neuroscience and Cell Biology (CNC-UC), Centre for Innovative Biomedicine and Biotechnology (CIBB), University of Coimbra, Coimbra, Portugal; 3 Collaborative Laboratory to Foster Translation and Drug Discovery (AccelBio), Cantanhede, Portugal; 4 School of Cardiovascular and Metabolic Medicine & Sciences, King’s College London, London, UK; 5 Centre for Bacterial Resistance Biology (CBRB), Department of Life Sciences, Imperial College London, London, UK

**Keywords:** Membranes & Trafficking, Microbiology, Virology & Host Pathogen Interaction, RNA Biology

## Abstract

MicroRNAs are increasingly recognised as important regulators of host–pathogen interactions. Using a large-scale functional screening approach, we identified miR-744-5p as a strong inhibitor of *Salmonella* infection. Time-course analyses in epithelial cells revealed that miR-744-5p moderately restricts early infection but exerts a much stronger inhibitory effect on late intracellular replication. This activity is conserved across *Salmonella* strains of distinct sequence types, whereas inhibition of *Shigella flexneri* is limited to early infection stages. Integrated transcriptomic analyses identified connexin 43 as a functionally relevant miR-744-5p target. Connexin 43 expression is strongly upregulated during *Salmonella* infection, and functional studies demonstrated that connexin 43 depletion selectively impaired late-stage intracellular replication, while CRISPRa-mediated connexin 43 upregulation enhanced bacterial proliferation. Notably, this function was independent of the canonical gap junction intercellular communication. Mechanistically, connexin 43 is required to maintain Golgi network organisation, enabling proper positioning of the *Salmonella*-containing vacuole. Thus, infection-induced downregulation of miR-744-5p promotes connexin 43 expression to support the formation of a permissive vacuolar replicative niche, revealing an unanticipated role for connexin 43 in Golgi homeostasis.

## Synopsis

Intracellular replication of *Salmonella* occurs at specialised vacuoles that are associated with the Golgi network. This study shows how host microRNA modulation upon *Salmonella* infection induces connexin 43 (Cx43) expression, thereby maintaining Golgi organisation and establishing a permissive intracellular replicative niche.

- Infection by *S. enterica* serovar Typhimurium downregulates miR-744-5p, leading to increased Cx43 expression during infection.
- Cx43 upregulation promotes the association of *Salmonella*-containing vacuoles with the Golgi network.
- Cx43 accumulates in the Golgi region during infection, revealing a non-canonical intracellular localisation of this gap junction protein.
- Cx43-dependent maintenance of Golgi organisation during infection promotes intracellular replication of *Salmonella*.

## Abstract (web summary)

A non-canonical role of Cx43 in regulation of Golgi organisation promotes establishment of the *Salmonella* replicative niche.

## Graphical abstract

**Figure:**
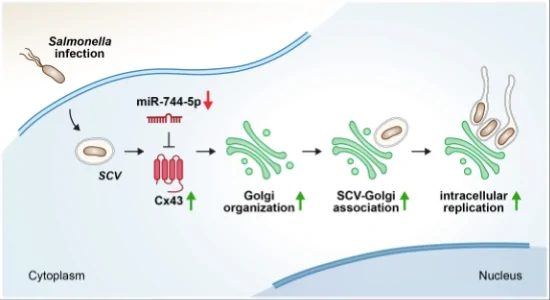

## Introduction

*Salmonella enterica* serovar Typhimurium (hereafter *Salmonella*) is a major pathogen responsible for substantial global morbidity and mortality (Collaborators GBDAR, 2022; Collaborators GBDN-TSID, 2019). In humans, salmonellosis presents with a broad spectrum of clinical outcomes, ranging from self-limiting gastroenteritis to severe and potentially fatal extraintestinal infections, depending on host susceptibility and bacterial strain.

*Salmonella* is a facultative intracellular pathogen that invades multiple cell types, including intestinal epithelial cells and immune cells (Castanheira and Garcia-Del Portillo, 2017; Han et al, 2024). Following invasion, *Salmonella* resides within a membrane-bound compartment known as the *Salmonella*-containing vacuole (SCV) (Steele-Mortimer, 2008; Tuli and Sharma, 2019). The SCV undergoes extensive remodelling during infection, towards the establishment of a fully functional replicative niche. A hallmark of this mature niche is its intimate interaction with host cellular structures, including the endolysosomal compartment, the cytoskeleton and the Golgi network (Han et al, 2024). In epithelial cells, *Salmonella* preferentially adopts an intravacuolar lifestyle, but cytosolic hyper-replication also occurs (Knodler et al, 2010; Malik-Kale et al, 2012). Invasion and intracellular vacuolar replication of *Salmonella* rely on two type III secretion systems (T3SSs) encoded within *Salmonella* pathogenicity islands 1 and 2 (SPI-1 and SPI-2), which deliver virulence effectors directly into host cells (Han et al, 2024; Jennings et al, 2017). SPI-1 effectors orchestrate bacterial entry and the early steps of vacuolar biogenesis, whereas SPI-2 effectors modulate diverse host pathways required for the maturation, maintenance, and stability of the replicative niche.

Among the host pathways altered during infection, regulation by microRNAs (miRNAs) has emerged as a key layer of control (Aguilar et al, 2019; Groeger et al, 2025). MiRNAs are small, non-coding RNAs that act as central post-transcriptional regulators of gene expression across diverse biological processes (Bartel, 2018; Quah et al, 2025; Shang et al, 2023). Their capacity to rapidly and coordinately modulate multiple components of a pathway or multiple pathways simultaneously makes miRNAs particularly relevant in contexts where cells must respond to complex environmental cues or stressors, such as in the framework of host–pathogen interactions. During bacterial infection, miRNAs can function as part of the host innate immune response but can also be subverted by pathogens to modulate the host environment in ways to favour bacterial pathogenesis (Aguilar et al, 2019). Multiple pathways, such as endosomal trafficking, actin polymerisation, autophagy, cell cycle and apoptosis, have been shown to be reprogrammed upon infection by bacterial pathogens, including *Listeria monocytogenes*, *Mycobacterium tuberculosis*, *Shigella flexneri*, and *Salmonella*, through manipulation of miRNA expression. Despite growing recognition of the broad importance of miRNAs in this context, the functional contributions of individual miRNAs to the establishment and maintenance of intracellular infection niches remain incompletely understood.

Our group has previously applied microscopy-based high-throughput screenings using comprehensive libraries of miRNA mimics to identify those involved in regulating infection by different bacterial pathogens, including *Salmonella* (Aguilar et al, 2020; Maudet et al, 2014). These studies revealed several miRNAs capable of modulating bacterial binding to host cells, and early and/or late stages of intracellular replication, with miR-744-5p emerging as a potent inhibitor of *Salmonella* infection.

Here, we characterise the role of miR-744-5p in *Salmonella* intracellular replication and identify the protein connexin 43 (Cx43, aka gap junction alpha-1 protein; encoded by the *GJA1* gene) as a biologically relevant target of this miRNA. Our work shows that the downregulation of miR-744-5p during infection leads to de-repression of Cx43, preserving stability of the Golgi network and its interaction with the SCV. This, in turn, supports the establishment of a robust vacuolar niche that favours *Salmonella* replication.

## Results

### MiR-744-5p inhibits early and late steps of *Salmonella* infection

**Figure 1:**
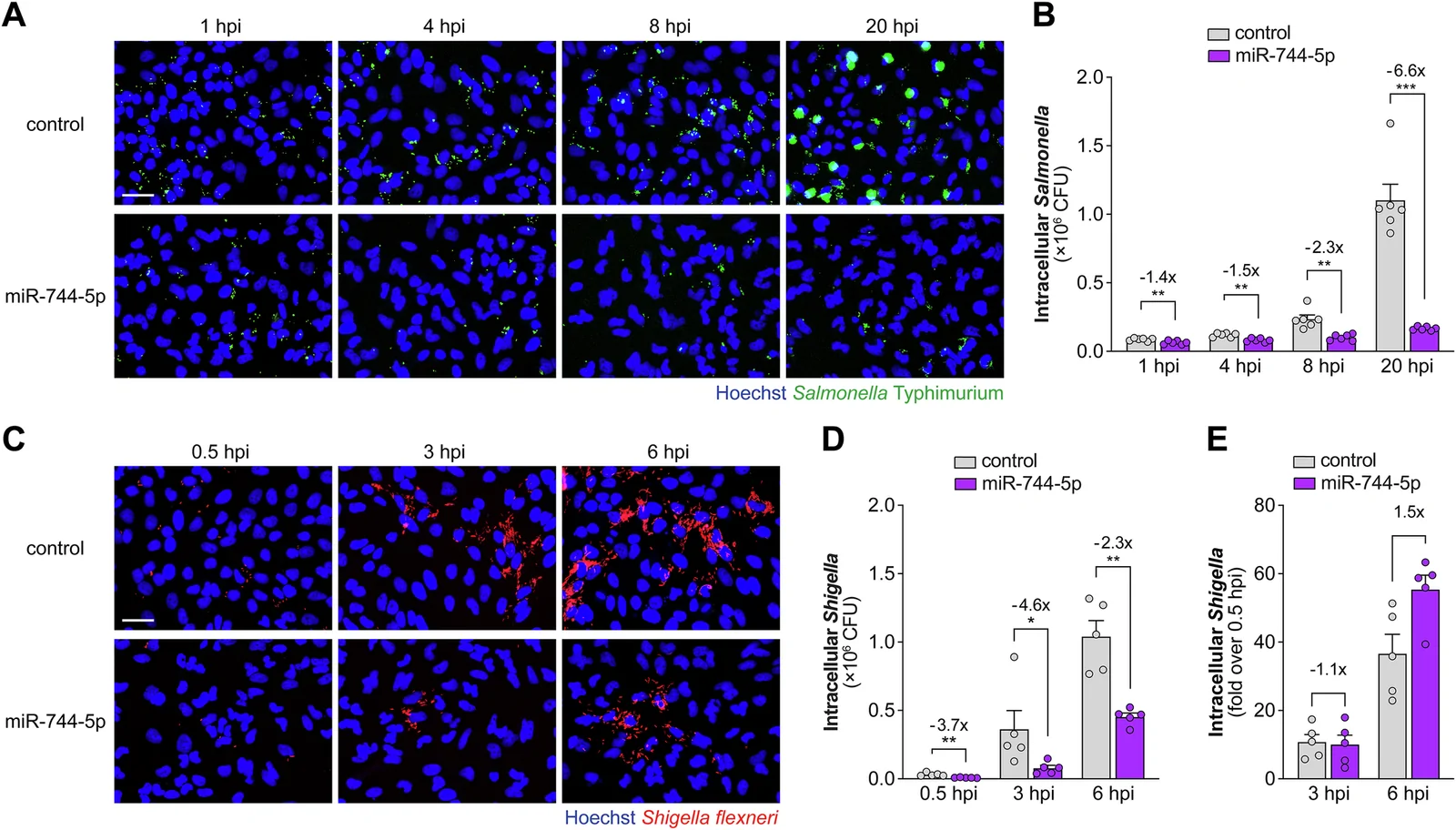
MiR-744-5p inhibits intracellular replication of *Salmonella*, but not that of *Shigella.* (**A**, **B**) Representative fluorescence microscopy images (**A**) and quantification of intracellular *Salmonella* by CFU (**B**) of HeLa cells transfected with control or miR-744-5p mimics, and infected with *Salmonella*, analysed at 1, 4, 8 and 20 hpi. (**C**–**E**) Representative fluorescence microscopy images (**C**) and quantification of intracellular *Shigella flexneri* by CFU (**D**, **E**) of HeLa cells transfected with control or miR-744-5p mimics, and infected with *Shigella*, analysed at 0.5, 3, and 6 hpi. CFU results in (**E**) are shown as fold-change normalised to 0.5 hpi. Scale bar, 50 µm. Results are shown as mean ± s.e.m. of *n* = 6 (**B**) or *n* = 5 (**D**, **E**) biologically independent experiments; microscopy images are representative of *n* = 3 (**A**, **C**) biologically independent experiments; *P < 0.05, **P < 0.01, and ***P < 0.001 (statistical analysis is detailed in Table EV2). Source data are available online for this figure.

**Figure EV1:**
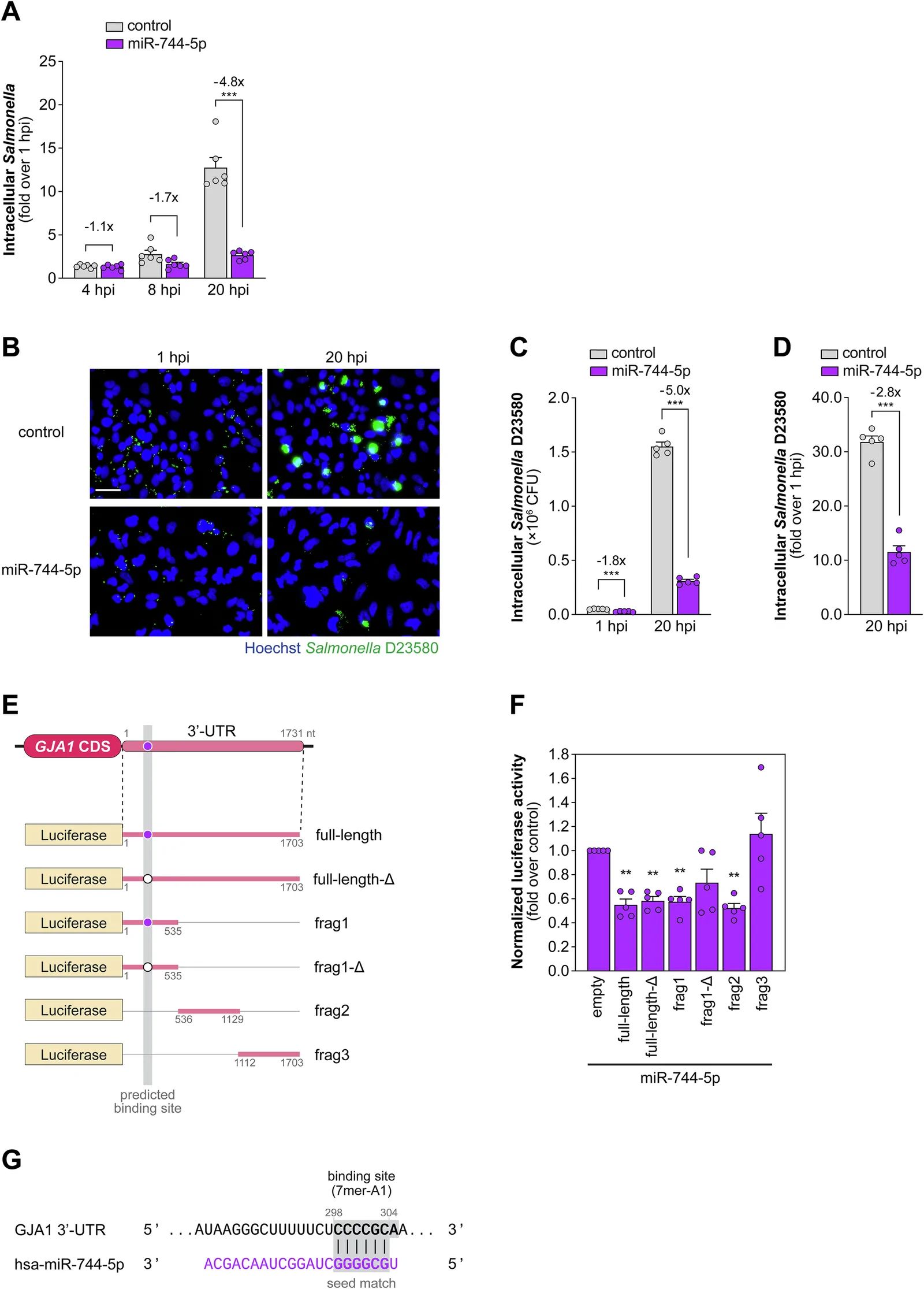
Cx43 is a direct target of miR-744-5p. (**A**) Quantification of intracellular *Salmonella* by CFU of HeLa cells transfected with control or miR-744-5p mimics, and infected with *Salmonella* and collected at 1, 4, 8 and 20 hpi. Results are shown as fold-change normalised to 1 hpi. (**B**–**D**) Representative fluorescence microscopy images (**B**) and quantification of intracellular *Salmonella* D23580 by CFU (**C**, **D**) of HeLa cells transfected with control or miR-744-5p mimics, and infected with *Salmonella* D23580, analysed at 1 and 20 hpi. CFU results in panel (**D**) are shown as fold-change normalised to 1 hpi. (**E**) Schematic representation of Cx43 3′-UTR and 3′-UTR fragments and mutant constructs used for the miRNA binding site reporter assays. The Cx43 3’-UTR was cloned downstream of Renilla luciferase in a dual-luciferase reporter plasmid (psiCHECK-2). (**F**) Results of the reporter assays using the Cx43 3’-UTR, fragments and mutant constructs. Results in cells treated with the miR-744-5p mimic are shown compared to the control miRNA. (**G**) Sequence alignment between miR-744-5p and the binding site in the 3’-UTR of human Cx43 sequence. Scale bar, 50 µm. Results are shown as mean ± s.e.m. of *n* = 6 (**A**) or *n* = 5 (**C**, **D**, **F**) biologically independent experiments; microscopy images are representative of *n* = 3 (**B**) biologically independent experiments; **P < 0.01, and ***P < 0.001 (statistical analysis is detailed in Table EV2).

In two microscopy-based screenings we have performed to identify miRNAs that regulate *Salmonella* infection, hsa-miR-744-5p consistently emerged as one of the top hits and among the strongest inhibitors of infection (Aguilar et al, 2020; Maudet et al, 2014). Time-course infection assays in epithelial cells, in which the *Salmonella* infection was analysed by fluorescence microscopy and intracellular load was quantified by CFUs at 4 times post-infection (1, 4, 8 and 20 hpi), confirmed the potent inhibitory activity of miR-744-5p on *Salmonella* infection (Fig. 1A,B). Notably, the reduction of intracellular bacterial load was already evident at 1 hpi, the earliest time post-infection tested (1.4-fold reduction), and became even more pronounced at later time points (2.3- and 6.6-fold reduction at 8 and 20 hpi, respectively) (Fig. 1A,B). Normalisation of CFUs to the 1 hpi values, thereby removing the effect of miR-744-5p in the early steps of infection, revealed that miR-744-5p robustly impairs intracellular *Salmonella* replication (4.8-fold reduction at 20 hpi) (Fig. EV1A).

The experiments described above were performed with *Salmonella* strain SL1344, a sequence type (ST) 19 strain, commonly used for studies on host–pathogen interaction (Kroger et al, 2012). Parallel assays using the *Salmonella* ST313 strain D23580, associated with invasive disease and high human mortality (Feasey et al, 2012; Kingsley et al, 2009), showed a similarly strong inhibitory effect of miR-744-5p (Fig. EV1B–D). As observed for *Salmonella* SL1344, the strongest inhibition was observed for intracellular replication (5.0-fold reduction at 20 hpi), with a more modest effect on early infection events (1.8-fold reduction at 1 hpi).

To assess whether the anti-infective activity of miR-744-5p is specific to *Salmonella*, we tested its effect on infection by the closely related enteropathogen *Shigella flexneri* (Fig. 1C–E). MiR-744-5p strongly inhibited early host–pathogen interaction (3.7-fold reduction at 0.5 hpi), but this effect diminished at later stages (2.3-fold reduction at 6 hpi). Indeed, normalisation of the CFU data to 0.5 hpi values indicates that miR-744-5p does not impair *Shigella* intracellular replication, but rather its inhibitory effect is restricted to early events in *Shigella* infection (Fig. 1E).

Overall, these results highlight that the effect of miR-744-5p in impairing late stages of intracellular replication is specific to *Salmonella*.

### Connexin 43 is a functionally relevant target of miR-744-5p during *Salmonella* infection

We previously showed that miR-744-5p expression is strongly downregulated upon *Salmonella* infection both in infected and bystander cells through a mechanism dependent on the decreased expression of the transcription factor E2F1 (Aguilar et al, 2021). Given the inhibitory activity of this miRNA towards *Salmonella* infection, particularly on intracellular replication, we argue that its downregulation represents a bacterial mechanism to promote infection.

**Figure 2:**
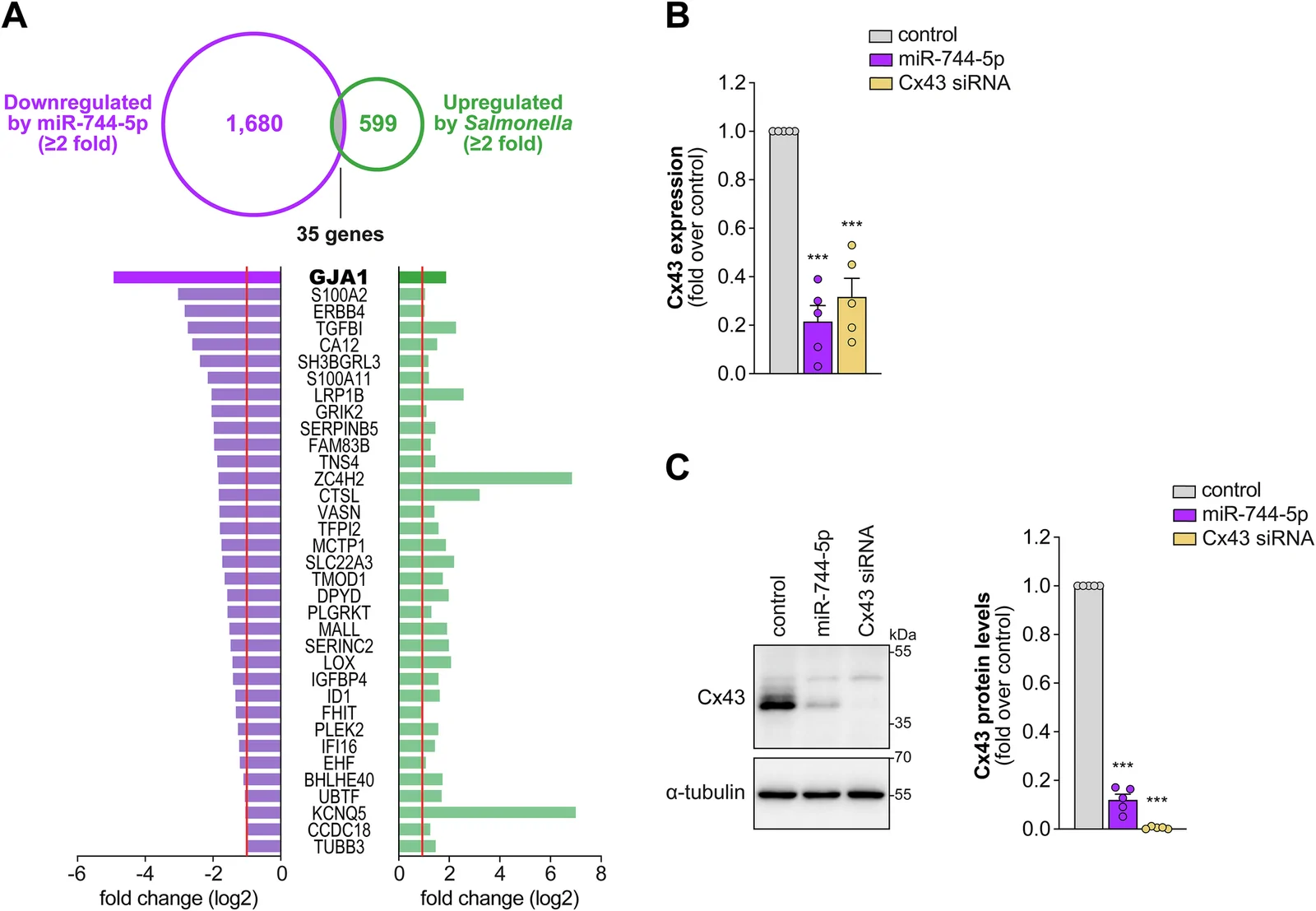
Comparative transcriptomics identifies Cx43 as a miR-744-5p target important during *Salmonella* infection. (**A**) Overlap between genes downregulated upon transfection of miR-744-5p mimics (≥ 2-fold compared to control miRNA, 1680 genes) and those upregulated upon *Salmonella* infection (≥ 2-fold compared to mock-treated cells; MOI 100, 20 hpi, 599 genes). The graph shows the expression changes of the 35 overlapping genes; red lines indicate the 2-fold thresholds. (**B**, **C**) Expression of Cx43, determined by RT-qPCR (**B**) or western blot (**C**) in HeLa cells transfected with control, miR-744-5p mimics or Cx43 siRNA. Results are shown as mean ± s.e.m. of *n* = 5 biologically independent experiments; ***P < 0.001 (statistical analysis is detailed in Table EV2). Source data are available online for this figure.

To obtain a comprehensive overview of miR-744-5p targets relevant during *Salmonella* infection, we performed a comparative transcriptomic analysis between the genes downregulated upon miR-744-5p overexpression and the genes upregulated upon *Salmonella* infection. Considering that miR-744-5p is downregulated during infection, we reasoned that a subset of genes upregulated in infected cells would represent de-repressed miR-744-5p targets. This approach identified 1680 genes downregulated by miR-744-5p (≥2-fold), and 599 genes upregulated during *Salmonella* infection (≥ 2-fold; 20 hpi, MOI 100), with an overlap of 35 genes constituting putative miR-744-5p targets potentially relevant during infection (Fig. 2A).

Among these, we focused on the most strongly downregulated gene, GJA1, which encodes for the protein connexin 43 (Cx43). Although best known for its canonical function in gap junction intercellular communication (GJIC), Cx43 also performs multiple gap junction-independent functions (Lucaciu et al, 2023; Martins-Marques et al, 2019; Zhu, 2022). However, the role of Cx43 in *Salmonella* intracellular replication has not been previously explored.

Consistent with Cx43 being a miR-744-5p target, its expression was strongly reduced in cells transfected with miR-744-5p mimics at both mRNA and protein levels (Fig. 2B,C). Direct targeting of Cx43 by the miRNA was confirmed by 3′-untranslated region (3’-UTR) luciferase reporter assays (Fig. EV1E,F). Adding to this, we validated one miR-744-5p predicted binding site in the Cx43 3’-UTR, identified by the bioinformatic tool TargetScan (Agarwal et al, 2015) (Fig. EV1G). This is a type 7mer-A1 site, characterised by a 6-nucleotide perfect base pairing of the target mRNA to the miRNA seed region (miRNA positions 2–7) and an adenine in the target mRNA opposite to the first nucleotide of the miRNA (Bartel, 2018; Lewis et al, 2005). Nonetheless, the observation that two non-overlapping Cx43 3′-UTR regions are regulated by miR-744-5p (fragments 1 and 2) suggests the presence of at least one additional functional binding site (Fig. EV1E,F).

**Figure 3:**
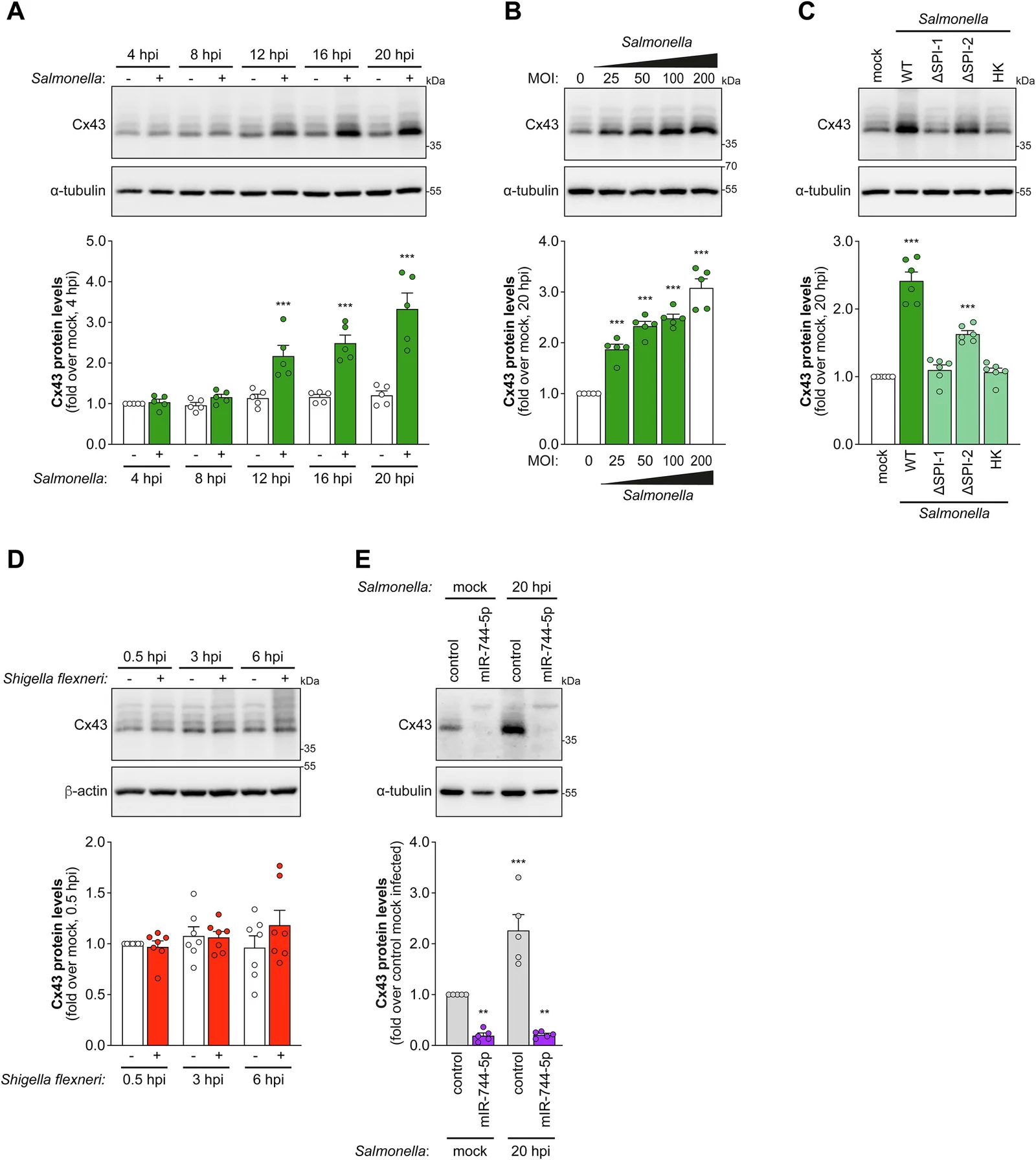
Cx43 upregulation during *Salmonella* infection requires bacterial internalisation. (**A**, **B**) Cx43 protein levels, determined by western blot, in HeLa cells infected with wild-type *Salmonella* (MOI 100) and analysed at 4, 8, 12, 16 and 20 hpi (**A**), or infected with increasing MOIs (25–200) and analysed at 20 hpi (**B**). (**C**) Cx43 protein levels, determined by western blot, in HeLa cells infected with wild-type *Salmonella* (WT), ΔSPI-1 or ΔSPI-2 mutant strains (MOI 100), or incubated with heat-killed (HK) *Salmonella*, and analysed at 20 hpi. (**D**) Cx43 protein levels, determined by western blot, in HeLa cells infected with *Shigella flexneri* (MOI 100) and analysed at 0.5, 3 and 6 hpi. (**E**) Cx43 protein levels, determined by western blot, in HeLa cells transfected with control or miR-744-5p mimics, and either mock-treated or infected with *Salmonella*, analysed at 20 hpi. Results are shown as mean ± s.e.m. of *n* = 5 (**A**, **B**, **E**), *n* = 6 (**C**) or *n* = 7 (**D**) biologically independent experiments; **P < 0.01, and ***P < 0.001 (statistical analysis is detailed in Table EV2). Source data are available online for this figure.

**Figure EV2:**
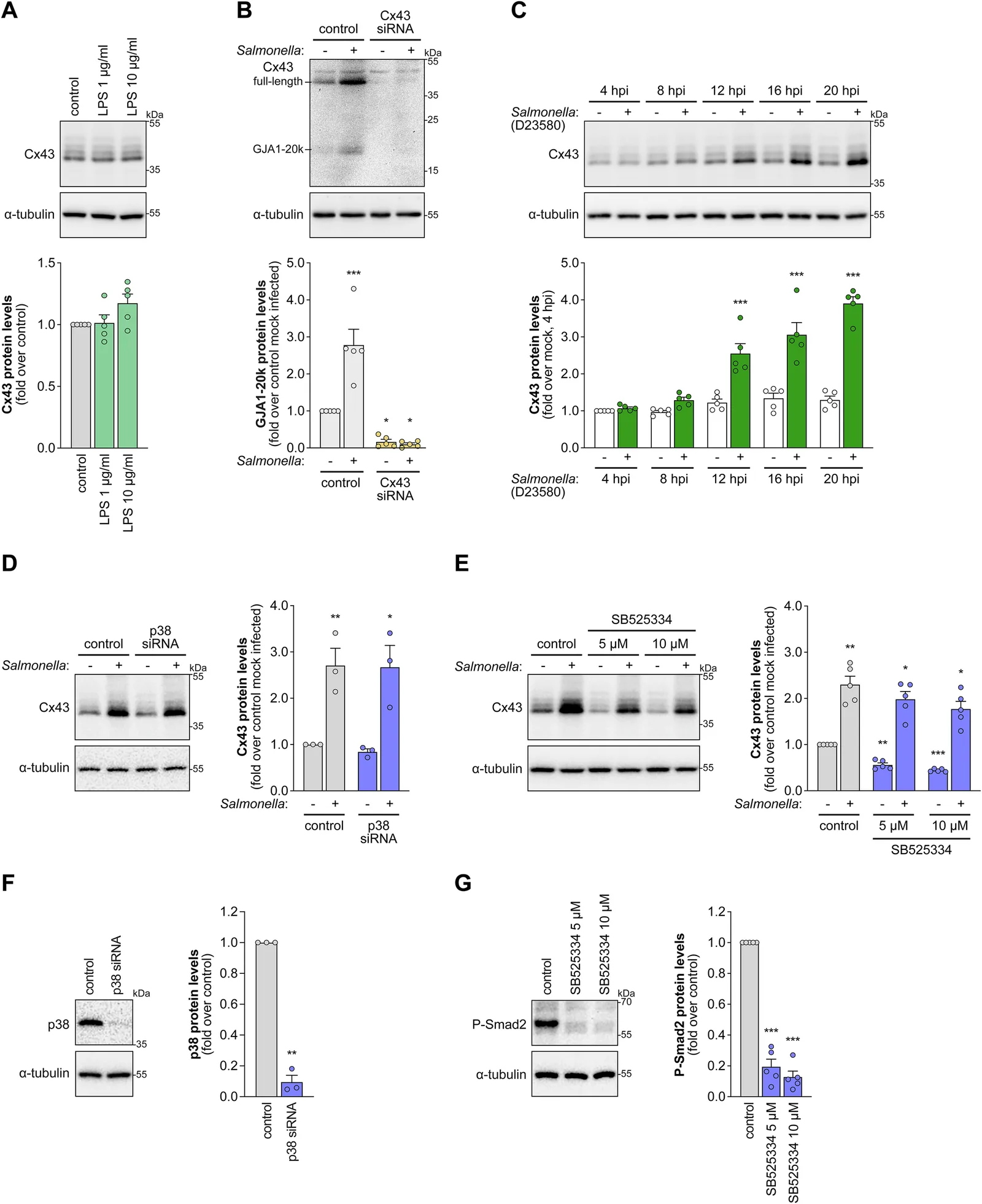
Cx43 is upregulated during *Salmonella* infection independently of p38 MAPK and TGF-β pathways. (**A**) Cx43 protein levels, determined by western blot, upon treatment with purified *Salmonella* LPS, for 24 h. (**B**) Cx43 protein levels, including the ~20 kDa Cx43 isoform (GJA1-20k), determined by western blot, in HeLa cells transfected with control or Cx43 siRNAs, and either mock-treated or infected with *Salmonella*, analysed at 20 hpi. (**C**) Cx43 protein levels, determined by western blot, in HeLa cells infected with *Salmonella* D23580 (MOI 100), analysed at 4, 8, 12, 16 and 20 hpi. (**D**) Cx43 protein levels, determined by western blot, in HeLa cells transfected with control or p38 siRNAs, and either mock-treated or infected with *Salmonella*, analysed at 20 hpi. (**E**) Cx43 protein levels, determined by western blot, in HeLa cells treated with the SB525334, a selective inhibitor of the TGF-β1 receptor, or vehicle, and either infected with *Salmonella* (20 hpi) or mock-treated. (**F**) p38 protein levels, determined by western blot, in HeLa cells transfected with control or p38 siRNAs. (**G**) Smad2 phosphorylation, determined by western blot, in HeLa cells treated with vehicle or SB525334. Results are shown as mean ± s.e.m. of *n* = 3 (**D**, **F**) or *n* = 5 (**A**, **B**, **C**, **E**, **G**) biologically independent experiments; *P < 0.05, **P < 0.01, and ***P < 0.001 (statistical analysis is detailed in Table EV2).

Consistent with the transcriptomics data, Cx43 protein levels were markedly increased upon *Salmonella* infection in a time- and MOI-dependent manner, becoming evident from 12 hpi onwards (Fig. 3A,B). Infection with the *Salmonella* ΔSPI-2 mutant strain, which is defective in intracellular replication, also increased Cx43 levels, whereas infection with the ΔSPI-1 mutant, defective in invasion, had no measurable effect on Cx43 levels (Fig. 3C). Heat-killed *Salmonella* and purified LPS also failed to increase Cx43 expression (Figs. 3C and EV2A). We also observed an infection-induced increase of a ~20 kDa Cx43 isoform (Fig. EV2B), mirroring the upregulation of full-length Cx43. Moreover, *Shigella* infection did not significantly alter Cx43 levels (Fig. 3D), whereas infection with *Salmonella* D23580 strongly increased Cx43 expression, similarly to *Salmonella* SL1344 (Figs. 3A and EV2C).

Importantly, miR-744-5p overexpression not only reduced basal Cx43 expression in non-infected cells but also prevented the increase in Cx43 protein expression observed following *Salmonella* infection (Fig. 3E). These findings support a model in which the *Salmonella*-induced downregulation of miR-744-5p is critical to Cx43 upregulation during infection.

We next assessed whether p38 MAPK or TGF-β pathways, both previously implicated in context-dependent regulation of Cx43 expression and known to be activated during *Salmonella* infection (Bruno et al, 2009; Chang et al, 2013; Larson et al, 1997; Tacheau et al, 2008; Zhang et al, 2018), contribute to the observed increase of Cx43 expression during *Salmonella* infection. Neither p38 knockdown nor treatment with SB525334, a selective inhibitor of the TGF-β1 receptor, prevented the Cx43 upregulation observed upon infection (Fig. EV2D,E). As previously reported, TGF-β pathway inhibition reduced basal Cx43 levels (Fig. EV2E). Efficient p38 knockdown and inhibition of TGF-β signalling (leading to decreased Smad2 phosphorylation) were confirmed by western blot (Fig. EV2F,G). These results suggest that, although p38 MAPK and TGF-β signalling can regulate Cx43 expression under specific cellular contexts, neither pathway appears to be a major driver of the infection-induced Cx43 upregulation observed in our experimental system. However, we cannot exclude the contribution of additional infection-induced signalling pathways to Cx43 regulation.

Together, these results demonstrate that Cx43 is a direct miR-744-5p target, and that its strong upregulation following *Salmonella* internalisation does not reflect a general response to infection by bacteria or bacterial products, but rather it is primarily driven by the reduction of miR-744-5p levels specifically triggered by *Salmonella* infection.

### Cx43 modulates *Salmonella* intracellular replication independently of its canonical function in gap junctions

**Figure 4:**
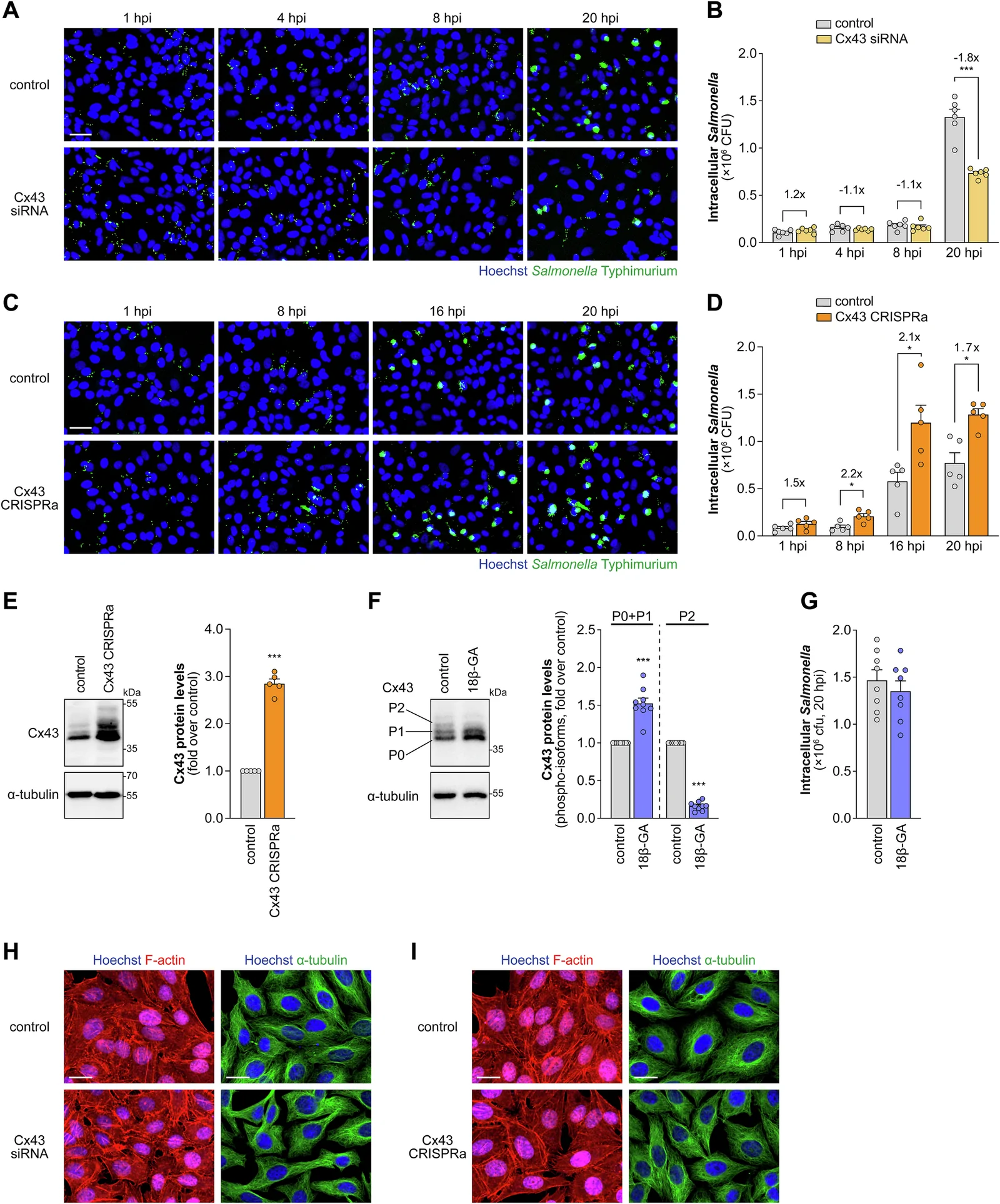
Modulation of *Salmonella* intracellular replication by Cx43 is independent of its function in intercellular communication through gap junctions. (**A**, **B**) Representative fluorescence microscopy images (**A**) and quantification of intracellular *Salmonella* by CFU (**B**) of HeLa cells transfected with control or Cx43 siRNAs, and infected with *Salmonella*, analysed at 1, 4, 8 and 20 hpi. (**C**, **D**) Representative fluorescence microscopy images (**C**) and quantification of intracellular *Salmonella* by CFU (**D**) of HeLa cells transfected with dCas9-VPR, and control or Cx43-targeting CRISPRa sgRNA, and infected with *Salmonella*, analysed at 1, 8, 16 and 20 hpi. (**E**) Expression of Cx43, determined by western blot, in HeLa cells transfected with dCas9-VPR, and control or Cx43-targeting CRISPRa sgRNAs. (**F**) Analysis of Cx43 phospho-isoforms, determined by western blot, in HeLa cells treated with 18β-GA or control (ethanol). (**G**) Quantification of intracellular *Salmonella* by CFU of HeLa cells treated with 18β-GA or control, and infected with *Salmonella*, analysed at 20 hpi. (**H**, **I**) Representative confocal fluorescence microscopy images of HeLa cells transfected with control and Cx43 siRNA (**H**), or transfected with dCas9-VPR and control or Cx43-targeting CRISPRa sgRNA (**I**) and stained for F-actin (phalloidin) or microtubules (α-tubulin). Scale bar, 50 µm (**A**, **C**) or 25 µm (**H**, **I**). Results are shown as mean ± s.e.m. of *n* = 5 (**D**, **E**), *n* = 6 (**B**), *n* = 8 (**G**) or *n* = 9 (**F**) biologically independent experiments; microscopy images are representative of *n* = 3 (**A**, **C**) or *n* = 2 (**H**, **I**) biologically independent experiments; *P < 0.05, and ***P < 0.001 (statistical analysis is detailed in Table EV2). Source data are available online for this figure.

**Figure EV3:**
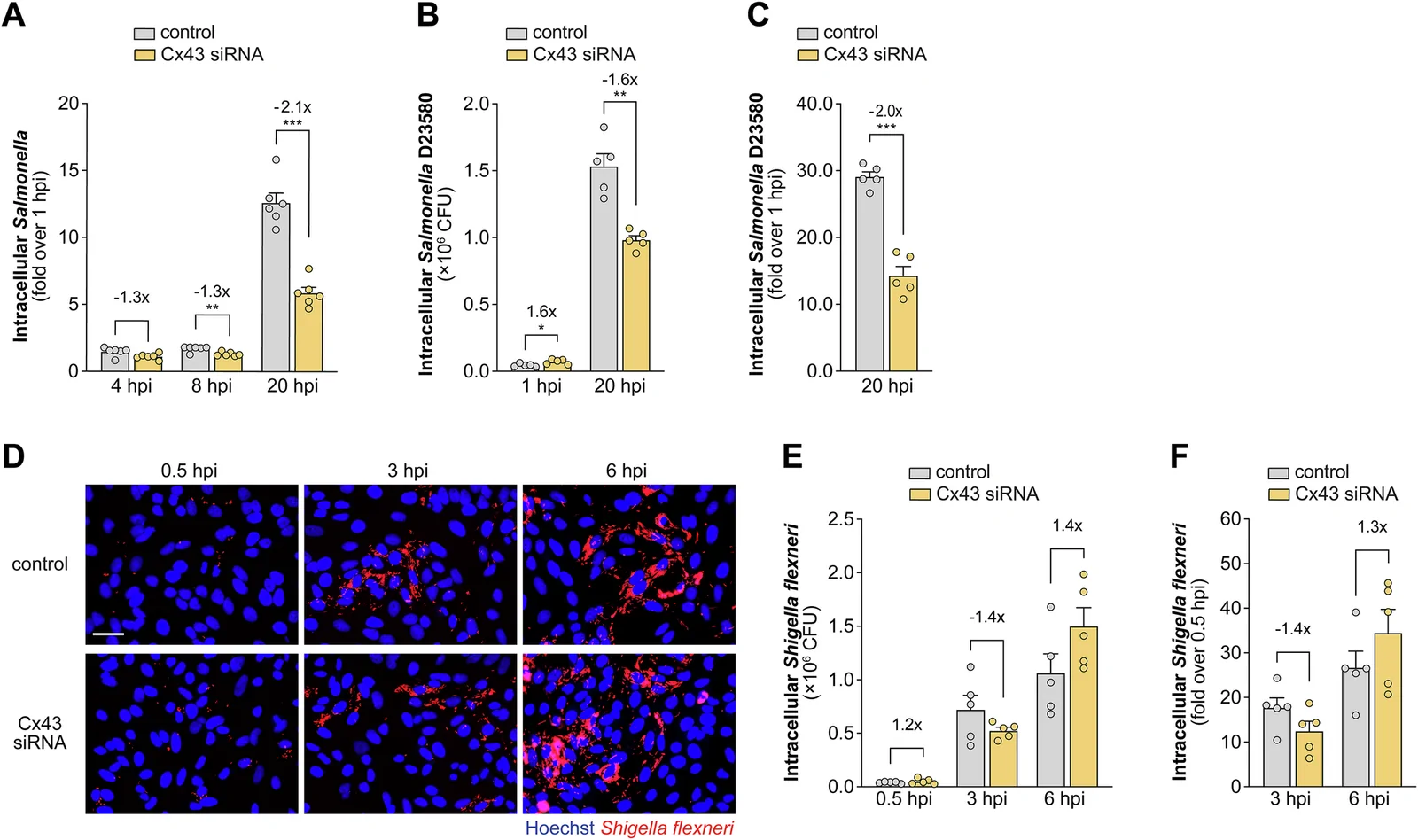
Cx43 knockdown does not inhibit *Shigella* infection. (**A**) Quantification of intracellular *Salmonella* by CFU of HeLa cells transfected with control or Cx43 siRNAs, and infected with *Salmonella*, analysed at 1, 4, 8 and 20 hpi. Results are shown as fold-change compared to 1 hpi. (**B**, **C**) Quantification of intracellular *Salmonella* D23580 by CFU of HeLa cells transfected with control or Cx43 siRNAs, and infected with *Salmonella*, analysed at 1 and 20 hpi. CFU results in (**C**) are shown as fold-change normalised to 1 hpi. (**D**–**F**) Representative fluorescence microscopy images (**D**) and quantification of intracellular *Shigella flexneri* by CFU (**E**, **F**) of HeLa cells transfected with control or Cx43 siRNAs, and infected with *Shigella*, analysed at 0.5, 3, and 6 hpi. CFU results in (**F**) are shown as fold-change normalised to 0.5 hpi. Scale bar, 50 µm. Results are shown as mean ± s.e.m. of *n* = 6 (**A**) or *n* = 5 (**B**, **C**, **E**, **F**) biologically independent experiments; microscopy images are representative of *n* = 3 (**D**) biologically independent experiments; **P < 0.01, and ***P < 0.001 (statistical analysis is detailed in Table EV2).

Given the strong upregulation of Cx43 during infection, we hypothesised that it plays an important functional role in *Salmonella* intracellular replication. Time-course infection assays revealed that Cx43 knockdown had no effect on *Salmonella* infection at early or intermediate times post-infection (1, 4 and 8 hpi), but markedly impaired intracellular replication at late stages (20 hpi) (Figs. 4A,B and EV3A). A similar result was observed during infection with the *Salmonella* D23580 (Fig. EV3B,C), whereas Cx43 knockdown resulted in a modest increase in *Shigella* burden at late time points (6 hpi) (Fig. EV3D–F). Conversely, increasing Cx43 expression using CRISPRa, enhanced *Salmonella* intracellular replication, with effects evident at 8, 16 and 20 hpi (Fig. 4C,D). Efficient Cx43 knockdown and overexpression were confirmed by western blot (Figs. 2B,C and 4E).

Given the well characterised role of Cx43 in gap junctions, we next tested whether GJIC contributes to *Salmonella* intracellular replication. To inhibit GJIC, we used 18β-glycyrrhetinic acid (18β-GA), which promotes Cx43 dephosphorylation and gap junction disassembly (Guan et al, 1996). As expected, 18β-GA treatment led to a strong reduction in the Cx43 P2 phospho-isoform (Fig. 4F), the phospho-isoform present in gap junctions characterised by phosphorylations at S325, S328, S330 and/or S364/365 (Solan and Lampe, 2018), alongside a corresponding increase of the P0 and P1 isoforms (Fig. 4F). P0 corresponds to the non-phosphorylated form of Cx43, and P1 to the isoform phosphorylated at S364 and/or S365 involved in trafficking to or within the plasma membrane. Importantly, inhibition of GJIC did not affect *Salmonella* intracellular replication (Fig. 4G).

Considering that Cx43 also performs several gap junction-independent functions, including regulation of cytoskeletal organisation (Basheer et al, 2017; Baum et al, 2025; Bazzoun et al, 2019; Francis et al, 2011; Rhee et al, 2009; Strauss and Gourdie, 2020), we investigated whether changes in actin or microtubule organisation could contribute to the effects of Cx43 on *Salmonella* replication. Knockdown and overexpression of Cx43 did not result in marked alterations in the overall organisation of F-actin or α-tubulin, as assessed by confocal fluorescence microscopy (Fig. 4H,I).

Overall, these results demonstrate that the modulation of Cx43 levels influences *Salmonella* intracellular replication through a mechanism that is independent of its canonical role in gap junction-mediated intercellular communication.

### Effect of Cx43 on *Salmonella* replication is independent of SPI-2 T3SS activation

*Salmonella* predominantly replicates within the *Salmonella*-containing vacuole (SCV), whereas *Shigella* rapidly escapes into the cytosol following invasion and replicates therein (Mellouk and Enninga, 2016). Considering that Cx43 knockdown impairs *Salmonella* replication but not that of *Shigella*, we hypothesised that the role of Cx43 during *Salmonella* infection might be linked to its vacuolar lifestyle. A critical step for SCV maturation and subsequent vacuolar replication is the activation of the *Salmonella* SPI-2 T3SS (Jennings et al, 2017).

**Figure 5:**
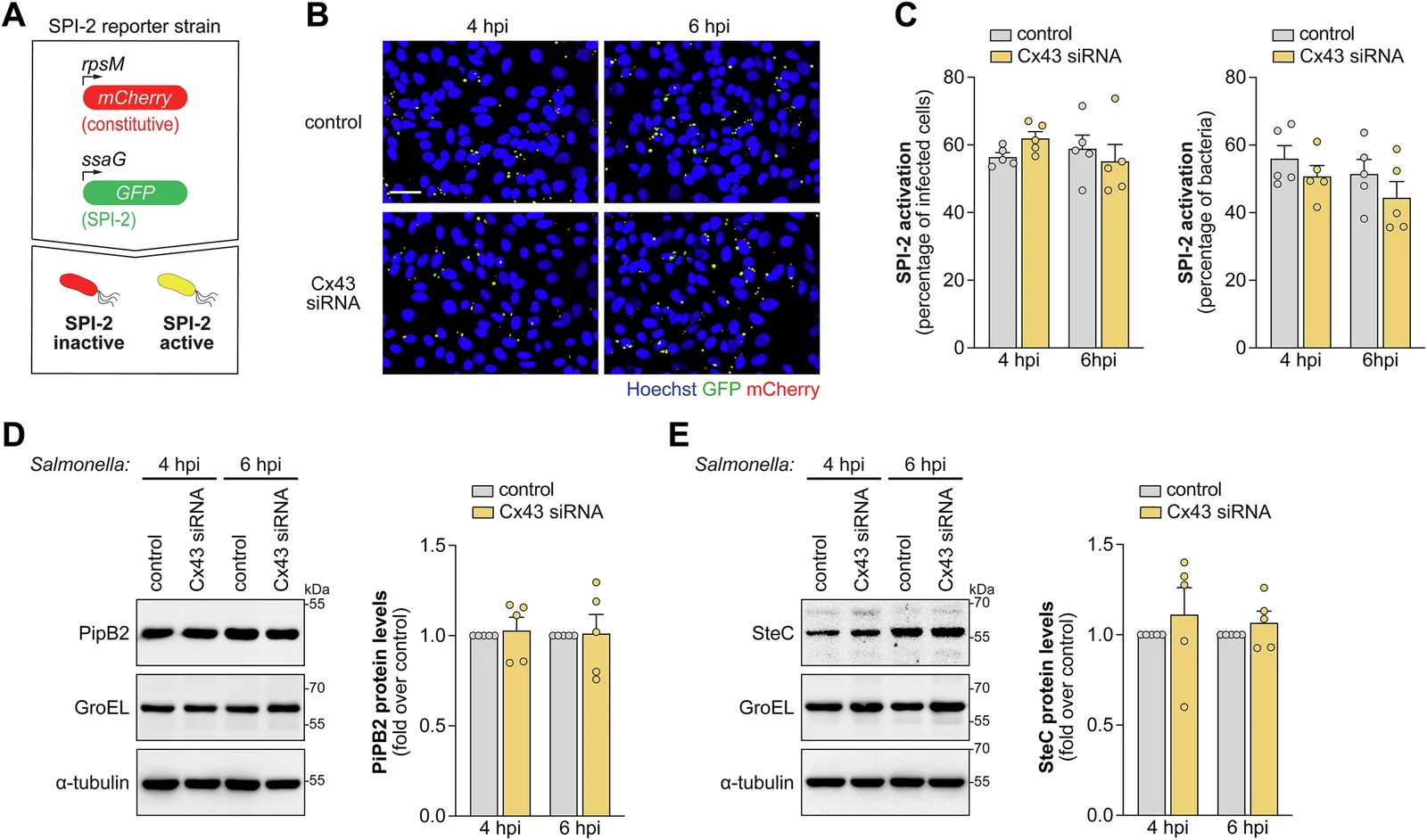
Cx43 depletion does not impair *Salmonella* SPI-2 T3SS activation. (**A**) Schematic representation of the SPI-2 T3SS activation reporter *Salmonella* strain. The strain harbours a plasmid that expresses GFP under the control of a SPI-2 T3SS gene promoter (*ssaG*), and a second plasmid that expresses mCherry from a constitutive promoter (*rpsM*). (**B**, **C**) Representative images of HeLa cells transfected with control or Cx43 siRNAs, and infected with *Salmonella* harbouring the SPI-2 T3SS reporter (**B**) and quantification of the percentage of *Salmonella*-infected cells with activated SPI-2 T3SS (**C**, left panel) or percentage of bacteria exhibiting SPI-2 activation (**C**, right panel). Infection was performed at MOI 50 and cells were analysed at 4 and 6 hpi. (**D**, **E**) Protein levels of the *Salmonella* SPI-2 T3SS effectors PipB2 (**D**) and SteC (**E**), determined by western blot, in HeLa cells transfected with control or Cx43 siRNAs, and infected with *Salmonella*. Infection was performed at MOI 100 and cells were collected at 4 and 6 hpi. Scale bar, 50 µm. Results are shown as mean ± s.e.m. of *n* = 5 biologically independent experiments; microscopy images are representative of *n* = 5 biologically independent experiments; statistical analysis is detailed in Table EV2. Source data are available online for this figure.

To evaluate whether Cx43 knockdown affects SPI-2 activation, we used two complementary approaches, specifically fluorescence microscopy using a *Salmonella* strain carrying a SPI-2 reporter plasmid, and analysis of SPI-2 effectors protein levels by western blot. The SPI-2 reporter expresses green fluorescent protein (GFP) under the control of the promoter of *ssaG*, a SPI-2 T3SS gene promoter, and constitutively expresses mCherry (Lisowski et al, 2022; Lober et al, 2006; Schlumberger et al, 2007) (Fig. 5A). Thus, GFP expression is only induced upon SCV maturation, whereas mCherry marks all the bacteria. Microscopy analysis indicates that SPI-2 activation occurred to a similar extent in control and Cx43 knockdown cells (Fig. 5B,C), both in terms of the percentage of infected cells containing SPI-2 activated bacteria and the percentage of bacteria exhibiting SPI-2 activation. Consistently, the protein levels of the SPI-2 effectors SteC and PipB2 were comparable between conditions (Fig. 5D,E).

Together, these results demonstrate that Cx43 depletion does not impair activation of *Salmonella* SPI-2 T3SS, a pre-requisite for vacuolar bacterial replication.

### Cx43 is required to preserve Golgi network organisation and SCV positioning during *Salmonella* infection, favouring bacterial replication

During *Salmonella* infection, SCVs migrate to the vicinity of the Golgi network (Abrahams et al, 2006; Deiwick et al, 2006; Ramsden et al, 2007; Salcedo and Holden, 2003). Key SPI-2 effectors, specifically SseF and SseG, mediate the SCV-Golgi association by binding to the Golgi-resident protein ACBD3 (Yu et al, 2016). Disruption of the Golgi-SCV tethering impairs *Salmonella* intracellular replication, highlighting its functional importance. We therefore hypothesised that Cx43 may influence Golgi organisation in a manner that determines the ability of *Salmonella* to establish its replicative niche.

**Figure 6:**
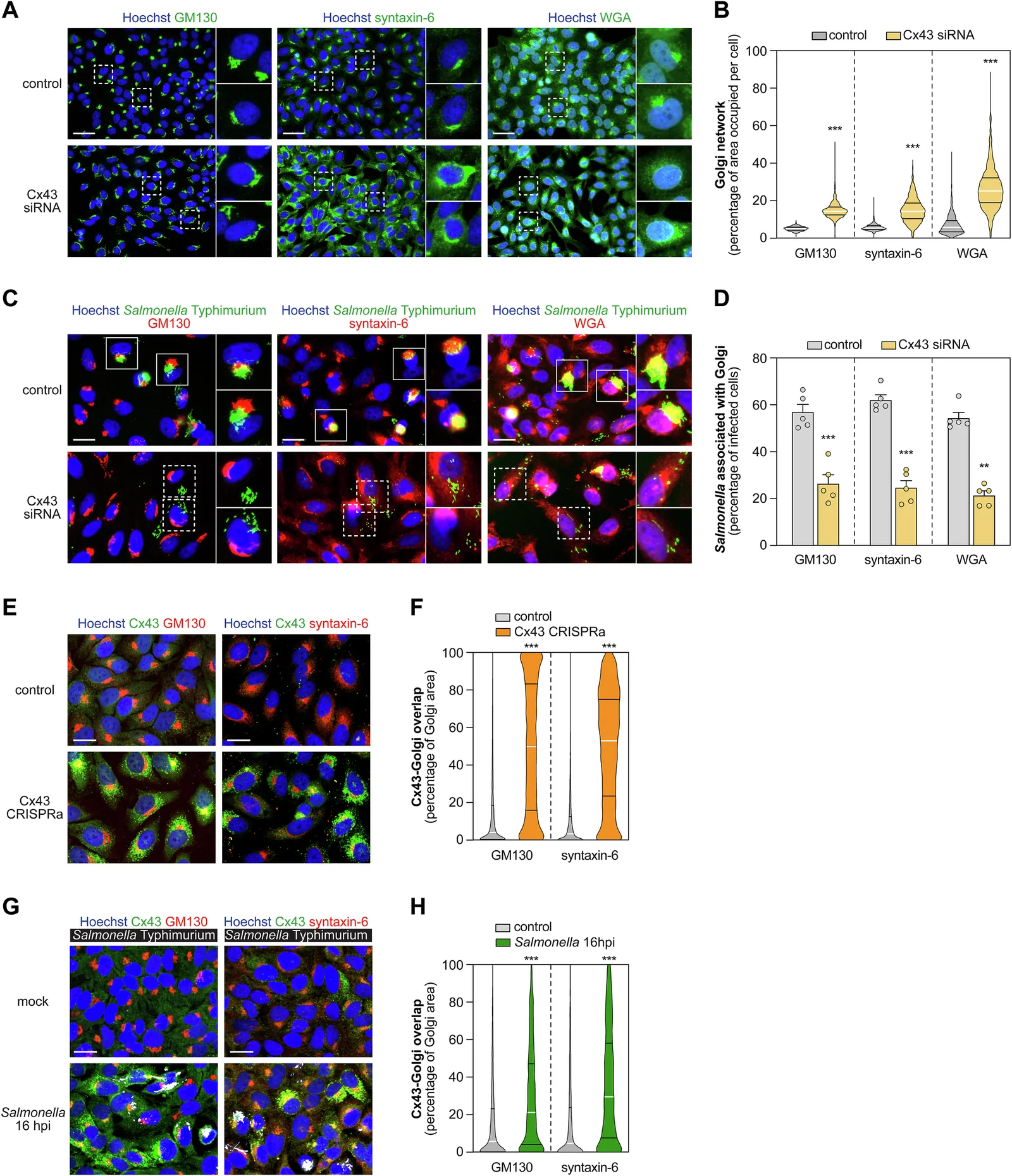
Cx43 knockdown disrupts Golgi network architecture and reduces SCV-Golgi association. (**A**, **B**) Representative images (**A**) and quantification of the area per cell occupied by the Golgi network (**B**) in HeLa cells transfected with control or Cx43 siRNAs and stained for various Golgi markers, specifically GM130, syntaxin-6 or WGA. Scale bar, 50 µm. Results are shown from >450 cells per condition and *n* = 5 independent experiments; white lines show the medians, black lines indicate the 25th and 75th percentiles, polygons extend to extreme values. ***P < 0.001 (statistical analysis is detailed in Table EV2). (**C**, **D**) Representative images (**C**) and quantification of *Salmonella*-infected cells in which the SCV is associated with Golgi (**D**) in HeLa cells transfected with control or Cx43 siRNAs, and infected with *Salmonella*, analysed at 16 hpi. Cells were stained for GM130, syntaxin-6 or WGA. Examples of cells classified as positive or negative regarding SCV-Golgi association are highlighted in solid and dashed lines, respectively. Scale bar, 25 µm. Results are shown from >70 cells per condition and *n* = 5 independent experiments. **P < 0.01, and ***P < 0.001 (statistical analysis is detailed in Table EV2). (**E**–**H**) Representative confocal fluorescence microscopy images (**E**, **G**) and quantification of the Cx43-Golgi overlap (shown as percentage of Golgi area occupied by Cx43; (**F**, **H**) in HeLa cells transfected with dCas9-VPR, and control or Cx43-targeting CRISPRa sgRNA (**E**, **F**), or in HeLa cells mock-treated or infected with *Salmonella* and analysed at 16 hpi (**G**, **H**). Cells were stained for Cx43 and either GM130 or syntaxin-6, as indicated. Scale bar, 25 µm. Results are shown from >1000 cells per condition and *n* = 2 (**G**, **H**) or *n* = 3 (**E**, **F**) biologically independent experiments; white lines show the medians, black lines indicate the 25th and 75th percentiles, polygons extend to extreme values. ***P < 0.001 (statistical analysis is detailed in Table EV2). Source data are available online for this figure.

**Figure EV4:**
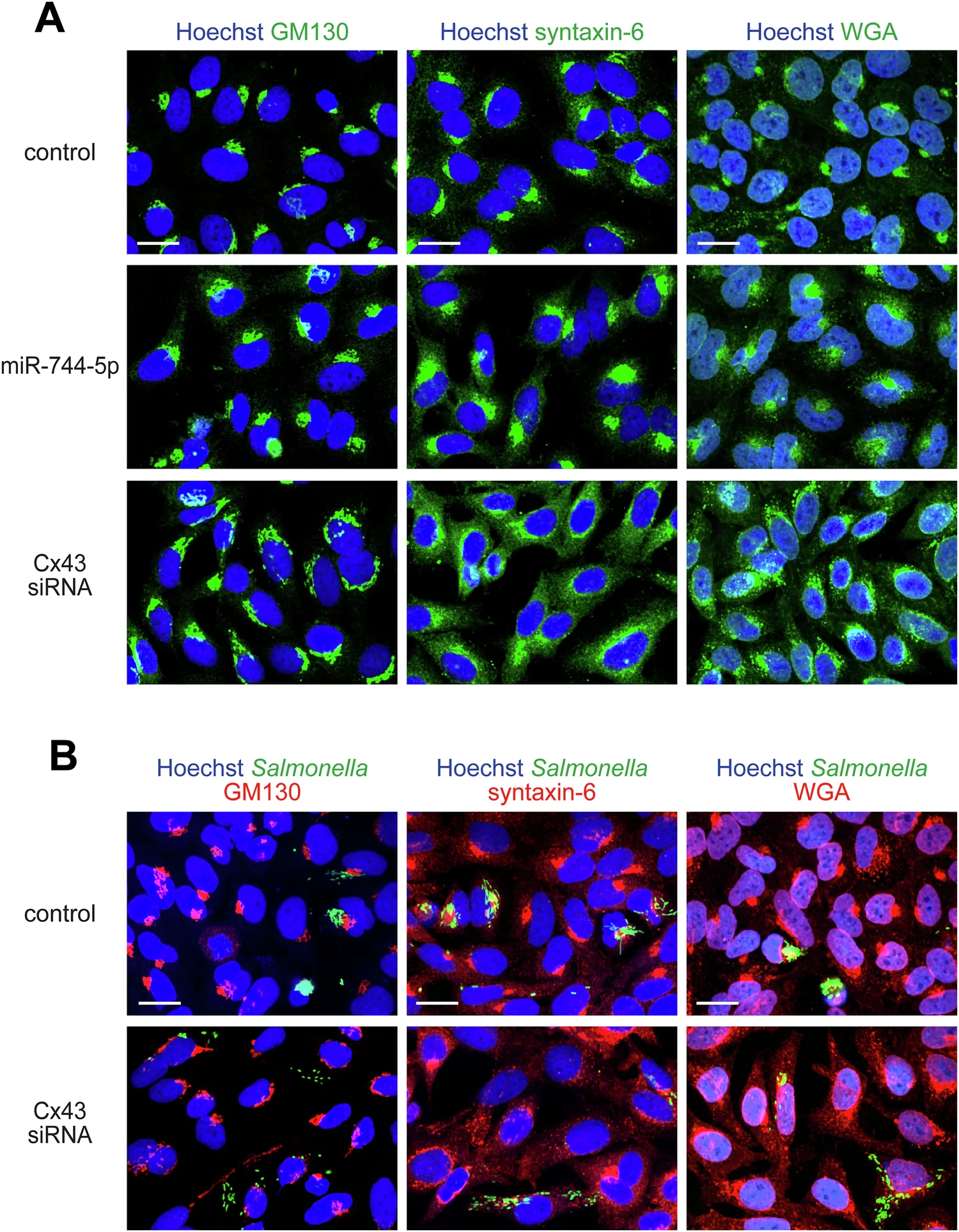
Cx43 depletion disturbs Golgi network organisation and SCV positioning during Salmonella infection. (**A**) Representative confocal fluorescence microscopy images of HeLa cells transfected with control, miR-744-5p mimics or Cx43 siRNA and stained for GM130, syntaxin-6 or WGA. (**B**) Representative confocal fluorescence microscopy images of HeLa cells transfected with control or Cx43 siRNAs, and infected with *Salmonella*, analysed at 16 hpi. Cells were stained for GM130, syntaxin-6 or WGA. Scale bar, 25 µm. Microscopy images are representative of *n* = 2 biologically independent experiments.

**Figure EV5:**
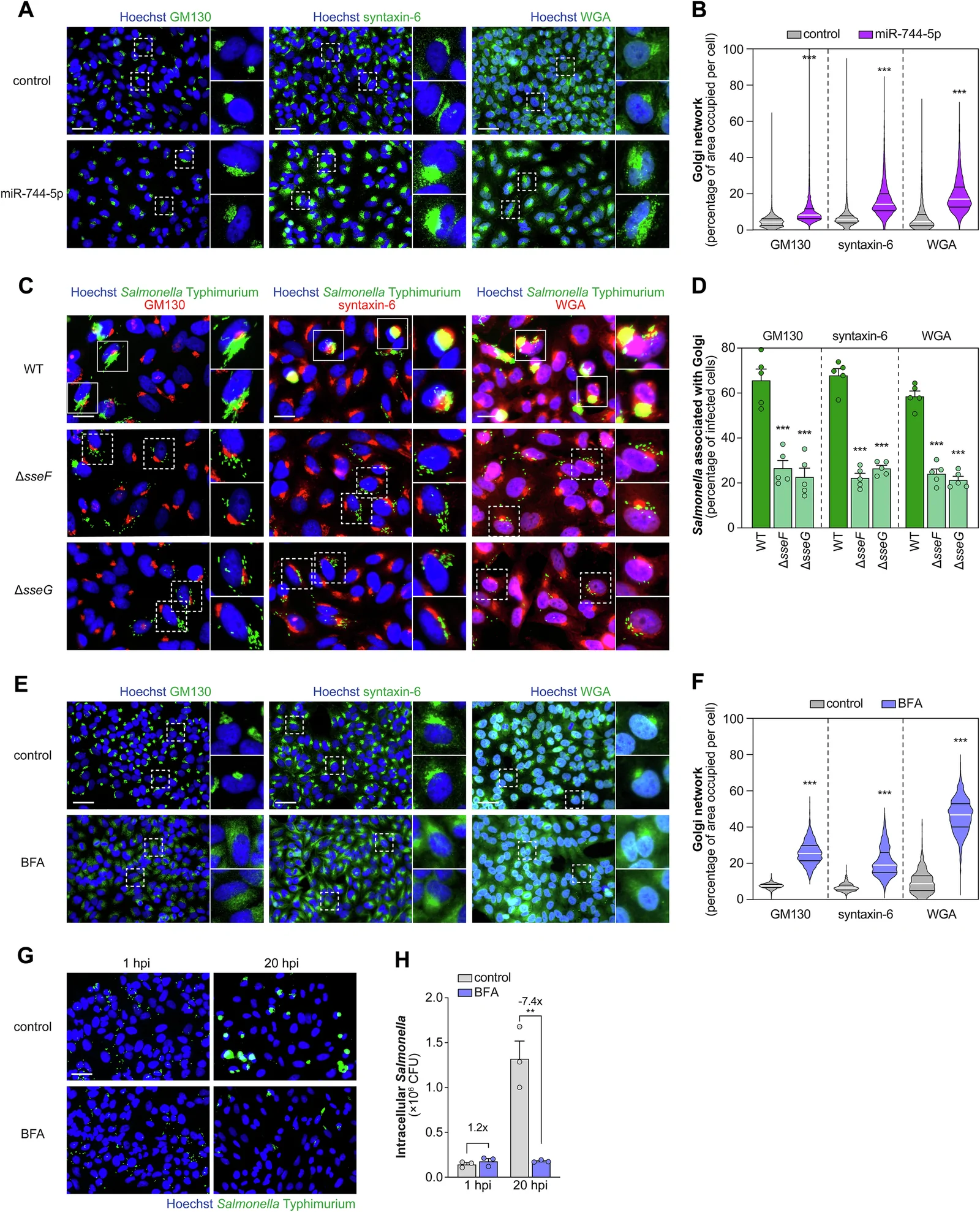
*Salmonella* Δ*sseF* and Δ*sseG* mutants present reduced SCV-Golgi association. (**A**, **B**) Representative images (**A**) and quantification of the area per cell occupied by the Golgi network (**B**) in HeLa cells transfected with control or miR-744-5p mimics and stained for various Golgi markers, specifically GM130, syntaxin-6 or WGA. Results are shown from >400 cells per condition and *n* = 7 independent experiments; white lines show the medians, black lines indicate the 25th and 75th percentiles, polygons extend to extreme values. (**C**, **D**) Representative images (**C**) and quantification of *Salmonella*-infected cells in which the SCV is associated with the Golgi network (**D**) of HeLa cells infected with wild-type (WT) *Salmonella*, or Δ*sseF* and Δ*sseG* mutants (MOI 50, analysed at 16 hpi). Cells were stained for GM130, syntaxin-6 or WGA. Examples of cells classified as positive or negative regarding SCV-Golgi association are highlighted in solid and dashed lines, respectively. (**E**, **F**) Representative images (**E**) and quantification of the area per cell occupied by the Golgi network (**F**) of HeLa cells treated with BFA or vehicle, and stained for the Golgi markers GM130, syntaxin-6 or WGA. Results are shown from >500 cells per condition and independent experiment (*n* = 5); white lines show the medians, black lines indicate the 25th and 75th percentiles, polygons extend to extreme values. (**G**, **H**) Representative fluorescence microscopy images (**G**) and quantification of intracellular bacteria by CFU (**H**) of HeLa cells infected with *Salmonella*, and treated with BFA or control, analysed at 1 and 20 hpi. Scale bar, 50 µm (**A**, **E**, **G**) or 25 µm (**C**). Results are shown as mean ± s.e.m. of *n* = 3 (**H**) or *n* = 5 (**D**) biologically independent experiments; microscopy images are representative of *n* = 3 (**G**), *n* = 5 (**C**, **E**) or *n* = 7 (**A**) biologically independent experiments; **P < 0.01, and ***P < 0.001 (statistical analysis is detailed in Table EV2).

To test this, we first assessed the impact of Cx43 knockdown on Golgi network architecture using three Golgi markers: the cis-Golgi protein GM130, the trans-Golgi protein syntaxin-6, and wheat germ agglutinin (WGA), a lectin that binds to N-acetylglucosamine residues enriched in the trans-Golgi network (TGN). In control cells, all markers exhibited the expected Golgi network compact perinuclear localisation (Figs. 6A and EV4A). In contrast, Cx43-depleted cells displayed a pronounced dispersal of the Golgi markers throughout the cytoplasm, reflected by a marked increase in the cell area occupied by the Golgi network (Figs. 6A,B and EV4A). Moreover, overexpression of miR-744-5p also resulted in partial Golgi dispersal, although this effect was less pronounced than that observed following Cx43 depletion by siRNA (Figs. EV4A and EV5A,B), likely reflecting the stronger reduction of Cx43 protein levels achieved by siRNA treatment (Fig. 2C).

We next examined whether this disrupted Golgi architecture affects the interaction between SCV and the Golgi network. Cx43 knockdown significantly reduced SCV-Golgi association (Figs. 6C,D and EV4B). This reduction was comparable to that observed for the *Salmonella* Δ*sseF* and Δ*sseG* mutants (Fig. EV5C,D), as previously reported (Abrahams et al, 2006; Deiwick et al, 2006; Ramsden et al, 2007; Salcedo and Holden, 2003; Yu et al, 2016), and therefore consistent with a functional defect in SCV-Golgi tethering. Of note, we used as a control for the requirement of an intact Golgi network for *Salmonella* intracellular growth cells treated with Brefeldin A (BFA), a drug that acutely disrupts Golgi structure. As previously reported (Salcedo and Holden, 2003), BFA treatment led to Golgi collapse and resulted in reduced bacterial replication (Fig. EV5E–H).

To further investigate the relationship between Cx43 and the Golgi apparatus, we examined the intracellular localisation of Cx43 by confocal microscopy during *Salmonella* infection or following CRISPRa-mediated Cx43 overexpression. Under both conditions, Cx43 showed increased accumulation in the perinuclear Golgi region, where it partially overlapped with the Golgi markers GM130 and syntaxin-6 (Fig. 6E–H). This redistribution is consistent with a role for Cx43 in regulating Golgi homeostasis independently of its canonical localisation at plasma membrane gap junctions.

Overall, these results demonstrate that Cx43 plays a previously unrecognised role in maintaining Golgi network architecture, which in turn is required for SCV positioning and the establishment of a productive intracellular replicative niche for *Salmonella*.

## Discussion

**Figure 7:**
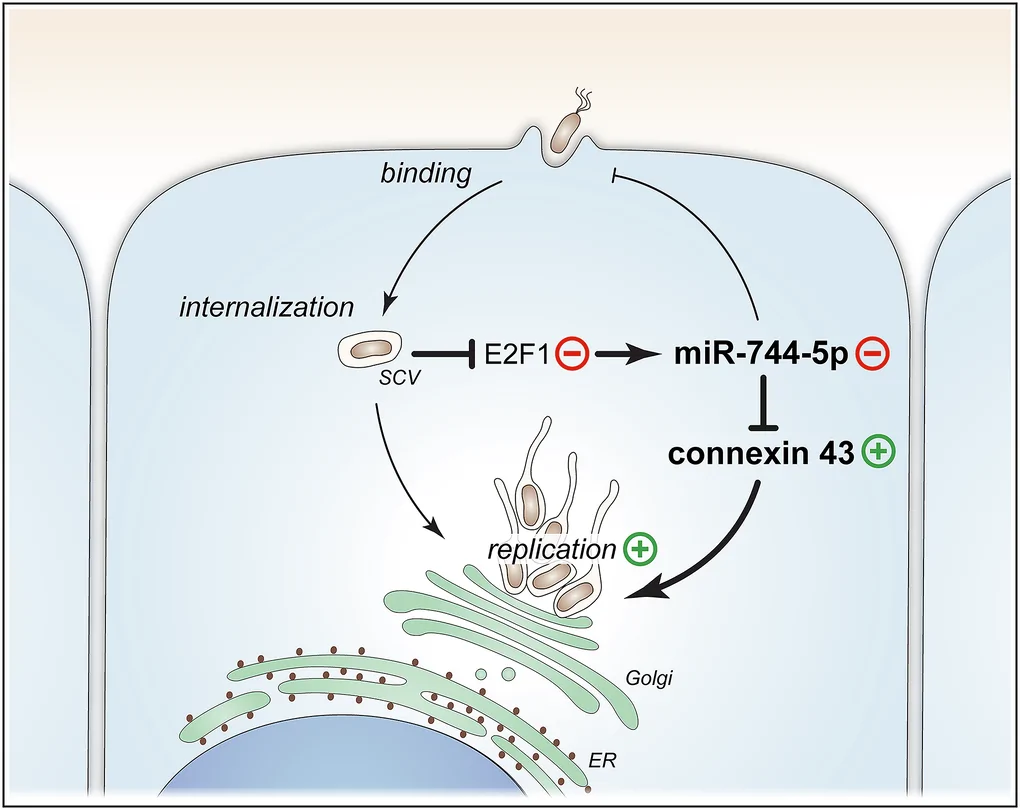
Model of miR-744-5p function during *Salmonella* infection. MiR-744-5p represses Cx43, leading to Golgi network disruption and reduced Golgi–SCV association, thereby limiting *Salmonella* intracellular replication. Infection-induced downregulation of miR-744-5p, through a mechanism dependent on the decreased expression of the transcription factor E2F1 (Aguilar et al, 2021), relieves Cx43 repression, promoting efficient vacuolar replication.

This study identifies the miR‑744‑5p/Cx43 axis as a central host pathway that shapes *Salmonella* intracellular niche by controlling Golgi architecture and SCV positioning. Functionally, miR‑744‑5p acts as a potent inhibitor of *Salmonella* infection, with moderate effects on early infection steps and a much stronger impact on late intracellular replication across distinct *Salmonella* sequence types, while its effect on *Shigella flexneri* is restricted to early infection events. Coupled with the infection‑driven downregulation of miR‑744‑5p via E2F1 in both infected and bystander cells (Aguilar et al, 2021), these findings support a model in which *Salmonella* actively suppresses miRNA expression to promote the establishment of its vacuolar replicative niche (Fig. 7). Within this framework, downregulation of miR‑744‑5p during *Salmonella* infection leads to de‑repression of host transcripts that are beneficial for bacterial intracellular replication, rather than a non‑specific consequence of infection.

Our integrated transcriptomic and functional analyses identified Cx43 as a functionally relevant miR‑744‑5p target. While Cx43 is well known for its canonical role in gap junction intercellular communication, extensive evidence supports multiple gap junction‑independent functions, including transcriptional regulation (Kotini et al, 2018; Martins-Marques et al, 2023), cytoskeletal organisation and cell polarity (Bazzoun et al, 2019; Francis et al, 2011; Rhee et al, 2009; Strauss and Gourdie, 2020), mitochondrial homeostasis and intercellular transfer (Islam et al, 2012; Zhang et al, 2022), long range communication (Ribeiro-Rodrigues et al, 2017), and autophagy (Bejarano et al, 2014). Recent work has also shown that Cx43 is recruited to damaged lysosomes, where it scaffolds actin nucleation complexes and promotes exocytosis of membrane‑injured, *Salmonella*‑containing lysosomes at early stages of infection, suggesting a host protective role (Domingues et al, 2024). In contrast, our study reveals a temporally distinct function for Cx43 in promoting *Salmonella* infection. We show that Cx43 expression is strongly and selectively upregulated at later times of *Salmonella* infection, and that manipulation of Cx43 levels bidirectionally modulates intracellular bacterial replication. Along this line, it was previously shown that *Salmonella* increases Cx43 expression and gap junction intercellular communication in tumour cells, enhancing chemotherapeutic sensitivity and facilitating cross-presentation of antigens in experimental cancer models (Chang et al, 2013; Saccheri et al, 2010). The observation that Cx43 knockdown impairs *Salmonella* but not *Shigella* replication supports the interpretation that this effect is linked to the vacuolar lifestyle of *Salmonella*, rather than to generic invasion or stress responses. Notably, our data indicate that the contribution of Cx43 to *Salmonella* replication is independent of its canonical role in gap junction intercellular communication, as pharmacological inhibition of gap junction function does not affect *Salmonella* replication.

The Golgi apparatus is increasingly recognised as a central target of bacterial pathogens, reflecting its essential role in membrane trafficking, protein secretion and lipid homeostasis. However, the strategies used to manipulate this organelle differ markedly among pathogens. In contrast to *Salmonella*, which requires maintenance of an intact Golgi network to support the establishment of its intracellular replicative niche, most bacterial pathogens described to date actively induce disruption of the Golgi architecture. For example, *Shigella flexneri* induces Golgi fragmentation through the activity of secreted effectors (Burnaevskiy et al, 2013; Mounier et al, 2012), including IpaB, which alters cholesterol distribution and disrupts Golgi-dependent trafficking (Mounier et al, 2012). Golgi perturbations have also been described for other bacterial pathogens, including *Chlamydia trachomatis*, *Legionella pneumophila*, *Rickettsia rickettsii*, *Streptococcus pyogenes*, *Streptococcus pneumoniae*, and *Anaplasma phagocytophilum* (Aistleitner et al, 2020; Heuer et al, 2009; Klabunde et al, 2023; Liu et al, 2021; Nozawa et al, 2021; Read et al, 2024). Collectively, these studies indicate that Golgi disruption is a common strategy employed by bacterial pathogens to remodel host cell physiology. Our findings therefore reveal a fundamentally different paradigm in which *Salmonella* exploits a host pathway to preserve Golgi integrity. By identifying Cx43 as a regulator of Golgi organisation required for efficient SCV positioning and intracellular replication, our study highlights maintenance, rather than disruption, of Golgi architecture as a key determinant of *Salmonella* pathogenesis.

Indeed, the importance of the Golgi apparatus for *Salmonella* infection is well established. After invasion, SCVs migrate towards the perinuclear region and establish close contact with the Golgi apparatus, a process critically dependent on the SPI‑2 effectors SseF and SseG (Abrahams et al, 2006; Deiwick et al, 2006; Ramsden et al, 2007; Salcedo and Holden, 2003; Yu et al, 2016). These effectors interact with the Golgi‑resident protein ACBD3, thereby tethering SCVs to Golgi‑derived membranes, a process essential for optimal bacterial replication (Yu et al, 2016). Mutations of SseF/SseG, ACBD3 depletion, or pharmacological Golgi disruption all reduce SCV-Golgi association and intracellular *Salmonella* growth. In this context, our work demonstrates that Cx43 knockdown leads to a pronounced dispersal of cis‑ and trans‑Golgi markers, significantly reduces SCV-Golgi association to levels comparable to Δ*sseF*/Δ*sseG* mutants, and impairs intracellular replication without affecting SPI‑2 T3SS activation. These findings identify Cx43 as a host determinant that preserves Golgi organisation, thereby enabling productive SCV positioning and supporting vacuolar replication. SCV association with the Golgi likely facilitates the delivery of lipids, proteins and/or fusion with Golgi network-derived endosomal tubules or vesicles, which would contribute to SCV maturation and sustain optimal bacterial replication (Yu et al, 2016).

Although Cx43 has not traditionally been recognised as a regulator of Golgi architecture, several lines of evidence are consistent with such a role. Cx43 interacts with cytoskeletal and scaffolding proteins that influence microtubule organisation and cell polarity, processes tightly coupled to Golgi architecture and positioning (Francis et al, 2011; Giepmans et al, 2001; Toyofuku et al, 1998). Cx43 also traffics through the secretory pathway, with Cx43 hexamers assembling in the trans‑Golgi network and being transported to the plasma membrane in Golgi‑derived vesicles, indicating that Cx43 transits through and can potentially influence this compartment (Epifantseva and Shaw, 2018). Moreover, it was recently reported that stable overexpression of Cx43 in HeLa cells causes disruption of trans-Golgi morphology (Sharma et al, 2023). The latter finding appears to contradict our results, in which Cx43 depletion disrupts Golgi organisation. This discrepancy likely reflects differences in cellular context and baseline Cx43 expression. Strong, non-physiological overexpression of Cx43 in cells that normally lack detectable Cx43 can induce cytoskeletal remodelling or aberrant channel clustering that alters Golgi morphology, whereas moderate, infection-induced upregulation of Cx43 may reinforce Golgi stability under the physiological demands of supporting an expanding SCV. The observation that baseline Cx43 expression varies substantially among HeLa clones further underscores the importance of cellular context in interpreting Cx43-dependent phenotypes (King et al, 2000). Interestingly, our analyses showed that full-length Cx43 and the ~20 kDa Cx43 isoform, GJA1-20k, were similarly upregulated during *Salmonella* infection. Although the possible contribution of GJA1-20k to the phenotypes described here remains to be determined, this isoform has been implicated in regulating cytoskeleton organisation and intracellular transport of full-length Cx43 (Basheer et al, 2017; Baum et al, 2025). However, we did not observe major changes in cytoskeletal organisation upon Cx43 depletion or overexpression in our experimental system, suggesting that alternative mechanisms concur to the effects of Cx43 on Golgi organisation during *Salmonella* infection. Determining the specific roles of the different Cx43 isoforms during *Salmonella* infection will be an important avenue for future investigation.

Beyond *Salmonella*, Cx43 has been implicated in infections by other bacteria. In enteric pathogens such as *Yersinia enterocolitica*, *Shigella flexneri*, and *Citrobacter rodentium*, Cx43 hemichannel phosphorylation or upregulation has been linked to bacterial invasion, dissemination and fluid secretion (Guttman et al, 2010; Tran Van Nhieu et al, 2003; Velasquez Almonacid et al, 2009). *Helicobacter pylori* relocalises Cx43 into autophagic vesicles via its VacA toxin, contributing to host cell death (Yahiro et al, 2015), while *Pseudomonas aeruginosa* drives JNK-dependent Cx43 gap junction signalling, propagating apoptotic signals from infected to neighbouring cells and thus amplifying barrier disruption (Losa et al, 2014). *Staphylococcus aureus* challenge reduces Cx43 expression and astrocyte gap junction communication (Esen et al, 2007), while *Streptococcus pneumoniae* infection involves Cx43-dependent astrocyte damage triggered by pneumolysin, contributing to blood-brain barrier disruption and inflammation (Bello et al, 2020). Cx43 is also required for efficient phagocytosis by macrophages, facilitating internalisation of opsonised particles and enhancing bacterial clearance (Anand et al, 2008). Importantly, our work reveals a distinct, pro-bacterial function for Cx43 during *Salmonella* infection, where it preserves Golgi organisation and SCV positioning to support vacuolar replication (Fig. 7).

In summary, this work identifies the miR-744-5p/Cx43 axis as a host pathway that *Salmonella* exploits to remodel organelle architecture and establish a permissive intracellular niche. By linking miRNA-dependent gene regulation to Golgi architecture and SCV positioning, our findings uncover a previously unrecognised layer of host control over vacuolar pathogen replication and expand current understanding of how intracellular bacteria co-opt cellular organisation to support their lifestyle. More broadly, the identification of Cx43 as a regulator of Golgi architecture reveals an unexpected gap junction-independent function for this protein, suggesting that Cx43-dependent control of Golgi organisation may be relevant to other physiological processes and disease contexts in which Golgi dynamics and intracellular trafficking are critical.

## Methods

**Table:** Reagents and tools table

Reagent/Resource	Reference or Source	Identifier or Catalog Number
**Experimental models**		
HeLa-229	ATCC	CCL-2.1
*Salmonella enterica* serovar Typhimurium strain SL1344 expressing GFP	Papenfort et al, 2009	
*Salmonella enterica* serovar Typhimurium strain D23580	Singletary et al, 2016	
*Salmonella* SL1344 strain used to monitor SPI-2 activation	Lisowski et al, 2022	
*Salmonella* SL1344 ΔSPI-1	Eulalio et al, 2011	
*Salmonella* SL1344 ΔSPI-2	Eulalio et al, 2011	
*Salmonella* SL1344 strains with chromosomal epitope-tagged (3xFLAG) SteC	Westermann et al, 2016	
*Salmonella* SL1344 strains with chromosomal epitope-tagged (3xFLAG) PipB2	Westermann et al, 2016	
*Shigella flexneri* serotype 5 strain M90T expressing mCherry	Tawk et al, 2018	
**Recombinant DNA**		
psiCHECK-2 dual luciferase reporter vector	Promega	C8021
psiCHECK-2 full-length GJA1 3’-UTR	This study	
psiCHECK-2 full-length GJA1 3’-UTR Δ	This study	
psiCHECK-2 frag1 GJA1 3’-UTR	This study	
psiCHECK-2 frag1 GJA1 3’-UTR Δ	This study	
psiCHECK-2 frag2 GJA1 3’-UTR	This study	
psiCHECK-2 frag3 GJA1 3’-UTR	This study	
**Antibodies**		
GM130	BD Biosciences	610822 (RRID:AB_398141)
syntaxin-6	Proteintech	10841-1-AP (RRID:AB_2196506)
β-actin	Sigma	A2228 (RRID:AB_476697)
α-tubulin	Sigma	T6074 (RRID:AB_477582)
FLAG	Sigma	F1804 (RRID:AB_262044)
GroEL	Sigma	G6532 (RRID:AB_259939)
GJA1	Origene	AB0016 (RRID:AB_3718694)
GJA1	Sigma	C6219 (RRID: AB_476857)
p38/MAPK14	Cell Signaling Technology	9212 (RRID:AB_330713)
Smad2 phospho S467	Abcam	ab280888 (RRID:AB_3099532
Chicken anti-Mouse IgG (H + L) Cross-Adsorbed Secondary Antibody, Alexa Fluor™ 488	ThermoFisher Scientific	A21200
Chicken anti-Mouse IgG (H + L) Cross-Adsorbed Secondary Antibody, Alexa Fluor™ 594	ThermoFisher Scientific	A21201
Chicken anti-Mouse IgG (H + L) Cross-Adsorbed Secondary Antibody, Alexa Fluor™ 647	ThermoFisher Scientific	A21463
Chicken anti-Rabbit IgG (H + L) Cross-Adsorbed Secondary Antibody, Alexa Fluor™ 488	ThermoFisher Scientific	A21441
Chicken anti-Rabbit IgG (H + L) Cross-Adsorbed Secondary Antibody, Alexa Fluor™ 594	ThermoFisher Scientific	A21442
Chicken anti-Rabbit IgG (H + L) Cross-Adsorbed Secondary Antibody, Alexa Fluor™ 647	ThermoFisher Scientific	A21443
Chicken anti-Goat IgG (H + L) Cross-Adsorbed Secondary Antibody, Alexa Fluor™ 488	ThermoFisher Scientific	A21647
Mouse IgG HRP Linked Whole Ab	GE Healthcare	NA931
Rabbit IgG HRP Linked Whole Ab	GE Healthcare	NA934
**Oligonucleotides and other sequence-based reagents**		
microRNA mimic negative control #4 (cel-miR-231)	Dharmacon/Horizon Discovery	CN-004000-01
microRNA hsa-miR-744-5p mimic	Dharmacon/Horizon Discovery	C-301242-01
SMARTpool siGENOME Human GJA1 siRNA	Dharmacon/Horizon Discovery	M-011042-01
SMARTpool siGENOME Human MAPK14	Dharmacon/Horizon Discovery	M-003512-06
SMARTpool CRISPRmod CRISPRa Human GJA1 Synthetic sgRNA	Dharmacon/Horizon Discovery	PG-011042-01
PCR primers	This study	Table EV1
**Chemicals, Enzymes and other reagents**		
DMEM containing 1 g/l glucose	HyClone	HyClone
Fetal bovine serum (FBS)	Gibco	10270-106
Gentamicin	Sigma	G1272
Lipofectamine RNAiMAX	ThermoFisher Scientific	13778030
CRISPRmod CRISPRa dCas9-VPR mRNA	Dharmacon/Horizon Discovery	CAS12211
Lipofectamine MessengerMAX	ThermoFisher Scientific	LMRNA015
FuGENE HD	Promega	E2311
Lipopolysaccharides (LPS) from *Salmonella enterica* serotype typhimurium	Sigma	L6143
18β-GA	Sigma	G10105
SB525334	TargetMol	T1763
Brefeldin A	ThermoFisher Scientific/ eBioscience	00-4506-51
Triton X-100	Roth	3051.4
Paraformaldehyde	Roth	0335.3
Bovine serum albumin (BSA)	Roth	8076.3
WGA conjugated with Alexa Fluor 555	ThermoFisher Scientific	W32464
WGA conjugated with Alexa Fluor 488	ThermoFisher Scientific	W11261
Alexa Fluor™ 568 Phalloidin	ThermoFisher Scientific	A12380
Hoechst 33342	ThermoFisher Scientific	H3570
Glo Lysis Buffer	Promega	E266A
Dual-Glo Luciferase Assay System	Promega	E2940
ECL Western Blotting Substrate	Pierce	32106
Sso Advanced Universal SYBR Green Supermix	Bio-Rad	172-5274
**Software**		
Harmony/Columbus image analysis software	PerkinElmer	
ImageJ	Schneider et al, 2012	
READemption pipeline version 0.3.0	Forstner et al, 2014	
Segemehl version 0.1.7	Otto et al, 2014	
DESeq version 1.18.0	Anders and Huber, 2010	
Microsoft Excel	Microsoft 365	
Prism software version 10.2.1	GraphPad	
Adobe Photoshop version 27.2	Adobe	
Adobe Illustrator version 30.1	Adobe	
**Other**		
24-well plates	Corning/Costar	3526
96-well plates	Corning/Costar	3799
384-well plates - black, clear-bottom	Greiner	781090
384-well plates - white	Corning	3570
Operetta automated high-content screening fluorescence microscope	Revvity/PerkinElmer	
Opera Phenix high-content screening fluorescence microscope	Revvity/PerkinElmer	
EnSpire Multimode plate reader	PerkinElmer	
Imager Chemi 5QE CCD camera	VWR	
Azure 600 Western Blot CCD Imaging System	Azure Biosystems	
CFX96 TouchTM Real-Time PCR detection system	Bio-Rad	

### Mammalian cell culture

Human epithelial HeLa-229 (ATCC, CCL-2.1) cells were cultured in DMEM containing 1 g/l glucose (HyClone, SH30021.01), supplemented with 10% fetal bovine serum (Gibco, 10270-106). Cells were maintained at 37 °C in a humidified atmosphere with 5% CO_2_. Cells tested negative for mycoplasma contamination and were not authenticated.

### Bacterial strains

The following bacterial strains were used in this study: *Salmonella enterica* serovar Typhimurium strain SL1344 expressing GFP constitutively from a chromosomal locus (Papenfort et al, 2009) or mCherry from pFPV-mCherry plasmid; a multidrug-sensitive derivative of *Salmonella enterica* serovar Typhimurium strain D23580 (kindly provided by F. Fang, University of Washington, USA) (Singletary et al, 2016), expressing mCherry from pFPV-mCherry plasmid; *Salmonella* SL1344 strain used to monitor SPI-2 activation (Lisowski et al, 2022), expressing GFP under the control of the SPI-2 T3SS gene *ssaG* promoter from pYC104 plasmid, and constitutively expressing mCherry under the control of the *rpsM* promoter from pFPV-mCherry plasmid; *Salmonella* SL1344 ΔSPI-1 and ΔSPI-2 mutant strains (Eulalio et al, 2011); Salmonella SL1344 Δ*sseF* and Δ*sseG* mutant strains (Eulalio et al, 2011); *Salmonella* SL1344 strains with chromosomal epitope-tagged (3xFLAG) SteC or PipB2 (kindly provided by J. Vogel, University of Würzburg, Germany) (Westermann et al, 2016); *Shigella flexneri* serotype 5 strain M90T expressing mCherry from pFPV-mCherry plasmid (Tawk et al, 2018). The pFPV-mCherry plasmid was kindly provided by Olivia Steele-Mortimer (Addgene #20956), and the pYC104 plasmid (Westermann et al, 2016) was kindly provided by J. Vogel, University of Würzburg, Germany.

*Salmonella* and *Shigella* were grown aerobically in LB-Miller broth. When appropriate, the liquid and solid media were supplemented with the following antibiotics: ampicillin 100 µg/ml, chloramphenicol 20 µg/ml, and kanamycin 25 µg/ml.

### miRNA/siRNA/sgRNA transfection

MiRNA mimics and siRNAs were transfected using a standard reverse transfection protocol (final miRNA/siRNA concentration of 50 nM) using Lipofectamine RNAiMAX (ThermoFisher Scientific, 13778030), as previously described (Aguilar et al, 2020). For RNA isolation, whole cell protein extracts, and CFU assays, cells were seeded in 24-well plates (Corning/Costar, 3526) at a cell density of 5.0 × 10^4^ per well; for microscopy, cells were seeded in black, clear-bottom 384-well plates (Greiner, 781090) at a density of 1.5 × 10^3^ cells per well; for dual-luciferase reporter assays, cells were seeded in 96-well plates (Corning/Costar, 3799) at a cell density of 1.2 × 10^4^ cells per well. Predesigned sgRNAs (final concentration of 75 nM) were co-transfected with 100 ng CRISPRmod CRISPRa dCas9-VPR mRNA (Dharmacon/Horizon Discovery, CAS12211) using a standard reverse transfection protocol with the transfection reagent Lipofectamine MessengerMAX (ThermoFisher Scientific, LMRNA015). For CFU assays and whole cell protein extracts, cells were seeded in 96- and 24-well plates at a cell density of 8.5 × 10^3^ and 5.0 × 10^4^ per well, respectively; for microscopy, cells were seeded in black, clear-bottom 384-well plates at a density of 1.5 × 10^3^ cells per well.

MicroRNA mimic negative control #4 (cel-miR-231; CN-004000-01), microRNA hsa-miR-744-5p mimic (C-301242-01), SMARTpool siGENOME Human GJA1 siRNA (M-011042-01), SMARTpool siGENOME Human MAPK14 (M-003512-06), SMARTpool CRISPRmod CRISPRa Human GJA1 Synthetic sgRNA (PG-011042-01) were purchased from Dharmacon/Horizon Discovery.

### Bacterial infection

Overnight *Salmonella* or *Shigella* cultures were diluted 1:100 in LB and grown at 37 °C until OD_600_ 2 for *Salmonella* and OD_600_ 0.4 for *Shigella*. Bacteria were then collected by centrifugation for 2 min at 12,000 × *g* and resuspended in complete medium. Cells were infected at MOI 50 (*Salmonella*) or 25 (*Shigella*) for microscopy, MOI 100 for western blot, and MOI 25 for CFUs. Following bacterial addition, the cells were centrifuged at room temperature for 10 min at 250 × *g* (*Salmonella*) or 15 min at 2000 × *g* (*Shigella*), and incubated at 37 °C in a 5% CO_2_ humidified atmosphere until a total of 30 min. Extracellular bacteria were killed by replacing the medium with fresh medium containing 50 µg/ml gentamicin for 30 min (time point 0 hpi), and then exchanged to medium containing 10 µg/ml gentamicin until analysis.

For LPS stimulation, HeLa cells were treated for 24 h with 1 µg/ml and 10 µg/ml LPS (Sigma, L6143). For stimulation with heat-killed *Salmonella*, bacteria were prepared as described above, except that before infection, bacteria were washed twice in PBS, incubated for 2 h at 80 °C, and then resuspended in complete medium. Heat-killing efficiency was verified by plating in LB agar plates.

For inhibition of gap junction intercellular communication, cells were treated with 75 µM 18β-GA (Sigma, G10105 during infection (added with 10 µg/ml gentamicin); to inhibit the TGF-β1 receptor, cells were treated with 5 µM and 10 µM SB525334 (TargetMol, T1763) for 24 h prior to infection and during infection (added with 10 µg/ml gentamicin); to disrupt Golgi structure, cells were treated with 10 µg/mL Brefeldin A (BFA, ThermoFisher Scientific/eBioscience, 00-4506-51) during infection (added with 10 µg/ml gentamicin).

### Colony forming unit (CFU) assays

HeLa cells were seeded in 24-well plates (Corning, 3526) at a density of 5 × 10^4^ cells per well 48 h before infection. Infections were performed as described above in the ‘Bacterial infection’ section. At the indicated times post-infection, cells were washed with PBS and lysed with PBS containing 0.1% Triton X-100 (Roth, 3051.4). Cell lysates were serially diluted in PBS and plated in LB plates.

### Staining and immunofluorescence

For immunofluorescence staining, at the indicated times post-infection, cells were rinsed with PBS and subsequently fixed with 4% paraformaldehyde (PFA) for 15 min at room temperature. Cells were permeabilised with 0.5% Triton X-100 in PBS for 10 min, followed by blocking in 1% bovine serum albumin (BSA; Roth, 8076.3) in PBS for 30 min. Cells were then stained with the following primary antibodies diluted in blocking solution overnight at 4 °C: GM130 (1:1000; BD Biosciences, 610822, RRID:AB_398141), syntaxin-6 (1:700; Proteintech, 10841-1-AP, RRID:AB_2196506), GJA1 (1:200; OriGene, AB0016, RRID:AB_3718694), GJA1 (1:250; Sigma, C6219, RRID:AB_476857), and α-tubulin (1:500; Sigma, T6074, RRID:AB_477582). Cells were then washed and incubated with the corresponding secondary antibodies conjugated with Alexa Fluor 488, 594, or 647 for 2 h at room temperature (1:250; ThermoFisher Scientific, A21200, A21201, A21463, A21441, A21442, A21443, A21647).

For the wheat germ agglutinin (WGA) staining, cells were fixed with 4% PFA for 30 min at room temperature and permeabilised 0.1% Triton X-100 in PBS for 10 min. Cells were then stained with WGA conjugated with Alexa Fluor 488 or 555 for 30 min at room temperature (3 µg/ml in PBS; ThermoFisher Scientific, W11261, W32464). For F-actin staining, following the permeabilisation step, cells were incubated for 30 min with Alexa Fluor 568 Phalloidin (1:400 in PBS; ThermoFisher Scientific, A12380).

Cell nuclei were counterstained with Hoechst 33342 (1:5000; ThermoFisher Scientific, H3570).

### Image acquisition and analysis

Widefield fluorescence image acquisition was performed using an Operetta automated high-content screening fluorescence microscope (Revvity/PerkinElmer), at 20× (for infection) or 40× magnification (for Golgi area, Golgi-Cx43 association and Golgi-SCV association); a total of 9 (20× magnification) or 15 images (40× magnification) were acquired per well and experiment. Confocal fluorescence imaging was performed using an Opera Phenix high-content screening fluorescence microscope (Revvity/PerkinElmer), equipped with a microlens-enhanced spinning disk confocal unit, at 40× magnification (water-immersion objective, NA 1.1); a minimum of 25 images were acquired per well and experiment. For phalloidin/F-actin, a 5-µm z-stack was acquired; the images shown represent maximum-intensity projections.

Automated image analysis was performed using custom workflows implemented in Harmony/Columbus image analysis software (PerkinElmer). Broadly, the building blocks ‘Find nuclei’, ‘Find cytoplasm’ and ‘Find spots’ were used to segment the different cellular compartments, Cx43 and bacteria, following filtering and pre-processing of the original images. Intensity and morphology properties of the regions of interest were extracted using ‘Calculate intensity properties’ and ‘Calculate morphology properties’ and used for classification.

For quantification of SPI-2 T3SS activation, two strategies were implemented: (i) to assess the percentage of infected host cells with SPI-2 T3SS-activated bacteria; and (ii) to determine the percentage of bacteria showing SPI-2 T3SS activation. To determine the percentage of infected cells with SPI-2 T3SS-activated bacteria/, the image analysis workflow was designed to identify and segment host cells (based on Hoechst stain that also generates a faint, low-intensity signal in the cytoplasm) and intracellular bacteria (based on the mCherry constitutive fluorescence signal), and then determine the total population of infected cells considering the quantified mCherry signal per host cell. The GFP fluorescence was then measured for the segmented bacteria to identify the population of host cells with bacteria expressing GFP. Data were expressed as the percentage of host cells with bacteria expressing both mCherry and GFP (infected cells with activated SPI-2 T3SS), relative to the total population of infected cells. To determine the percentage of bacteria showing SPI-2 T3SS activation, individual intracellular bacteria were identified and segmented based on the constitutive mCherry fluorescence signal. The GFP fluorescence was then measured for the segmented bacteria to determine those expressing GFP. Data are expressed as the percentage of bacteria expressing both mCherry and GFP (activated SPI-2 T3SS), relative to the total number of segmented bacteria.

For the quantification of the area occupied by the Golgi network, a workflow was designed to identify and segment the host cells (as described above) and the different regions of the Golgi compartment (based on staining with specific markers: WGA and syntaxin-6 for trans-Golgi, GM130 for cis-Golgi). After segmentation, the total area of each cell and the corresponding area of the Golgi compartment were determined. The area occupied by the Golgi compartment was expressed as percentage of the total area of the corresponding cell, on a per cell basis. For the quantification of Golgi/Cx43 overlap, the region occupied by Cx43 was segmented and the area of Cx43 present within the Golgi compartment (segmented as described above) was determined. Data are expressed as percentage of the Golgi area occupied by Cx43, on a per cell basis.

The analysis of Golgi-SCV association was performed manually using the ImageJ manual classifier (Schneider et al, 2012), from at least 70–130 infected cells per condition and independent experiment. For the classification, the overall intracellular infection pattern was evaluated for each infected cell. Cells were classified as positive regarding SCV-Golgi association when the majority of SCVs were either superimposed on or in proximity to the Golgi apparatus, as visualised by the Golgi marker. In contrast, cells were classified as negative regarding SCV-Golgi association when SCVs were distributed heterogeneously throughout the cytoplasm, even if a subset of bacteria was located near the Golgi region. Thus, the classification reflected the predominant spatial distribution of the SCVs within each infected cell rather than the position of individual bacteria. Examples of cells classified as positive or negative regarding SCV-Golgi association are highlighted in Figs. 6C and EV5C.

For all the analyses, cells touching the edge of the images or exhibiting abnormal nuclear morphology were excluded from the analysis. For consistency across figures, microscopy images were pseudo-coloured, with *Salmonella* infection shown in green and *Shigella flexneri* infection shown in red.

### Dual-luciferase reporter assay

The full-length GJA1 3’-UTR was amplified by PCR from a HeLa cDNA library and cloned into the psiCHECK-2 dual luciferase reporter vector (Promega, C8021) using XhoI and NotI restriction sites. Fragments of GJA1 3’-UTR were amplified by PCR from the psiCHECK-2 full-length GJA1 3’-UTR construct. The deletion mutant of miR-744-5p predicted binding site in the 3’-UTR of GJA1 was generated by site-directed mutagenesis. All primers used for cloning and mutagenesis are shown in Table EV1.

HeLa-229 cells were transfected with control miRNA or miR-744-5p using a standard reverse transfection protocol (final concentration of 50 nM) as described in the ‘miRNA/siRNA transfection’ section. Twenty-four hours later, cells were transfected with 50 ng of the reporter plasmids containing the full-length (wild-type or mutated sequences) or fragments of GJA1 3’-UTR using FuGENE HD transfection reagent (Promega, E2311), according to the manufacturer’s instructions. Forty-eight hours after plasmid transfection, cells were lysed using Glo Lysis Buffer (Promega, E266A). Cell lysates were transferred to white 384-well plates (Corning, 3570), and Firefly and Renilla luciferase activities were measured on an EnSpire Multimode plate reader (PerkinElmer), using the Dual-Glo Luciferase Assay System (Promega, E2940), according to the manufacturer’s instructions. For cells transfected with miR-744-5p, the ratio of Renilla/Firefly luciferase activity was normalised to that of cells transfected with control miRNA and then normalised to the results obtained with the control plasmid (empty psiCHECK-2 plasmid).

### Protein extracts and western blot

For protein extracts, cells were washed with PBS and directly lysed in Laemmli’s sample buffer. Samples were sonicated, heat-denatured for 5 min at 95 °C, and separated in SDS-PAGE followed by western blot. The following primary antibodies were used: β-actin (1:5000; Sigma, A2228, RRID:AB_476697), α-tubulin (1:3000; Sigma, T6074, RRID:AB_477582), FLAG (1:1000; Sigma, F1804, RRID:AB_262044), GroEL (1:10,000; Sigma, G6532, RRID:AB_259939), GJA1 (1:1000; OriGene, AB0016, RRID:AB_3718694), p38/MAPK14 (1:2000; Cell Signaling Technology, 9212, RRID:AB_330713), Smad2 phospho S467 (1:1000; Abcam, ab280888, RRID:AB_3099532). The following secondary antibodies coupled to horseradish peroxidase were used: anti-mouse, anti-rabbit (1:10,000; GE Healthcare, NA931 and NA934, respectively), and anti-goat antibodies (1:5000; Invitrogen, 81-1620). Signals were detected with ECL Western Blotting Substrate (Pierce, 32106) using an Imager Chemi 5QE CCD camera (VWR) or an Azure 600 Western Blot CCD Imaging System (Azure Biosystems). Quantification was performed with ImageJ. β-actin/α-tubulin and GroEL were used as loading controls for human and bacterial cells, respectively. All uncropped immunoblots are provided in Source Data.

### RNA isolation, cDNA synthesis and quantitative real-time PCR

RNA extraction, cDNA synthesis, and qPCR were performed as described previously (Aguilar et al, 2021). qRT-PCR was performed using Sso Advanced Universal SYBR Green Supermix (Bio-Rad, 172-5274) according to the manufacturer’s instructions, using a CFX96 TouchTM Real-Time PCR detection system (Bio-Rad). The following primer pairs were used: GJA1, 5′-TGAAGCAGATTGAGATAA-3′ and 5′-ATAGACTTGAAGAGGATAC-3′; RPL37A, 5′-AAGACTGAAGGAGTTGAA-3′ and 5′-TTTGTTACATAAATTAACCCATT-3′. Expression was normalised to RPL37A, and the relative gene expression was calculated using the 2^−ΔΔCt^ method.

### RNA sequencing and computational RNA-seq analysis

Library preparation and deep-sequencing were performed by Vertis Biotechnology AG, as described previously (Maudet et al, 2014). Data analysis was performed as described previously (Maudet et al, 2014), using the READemption pipeline version 0.3.0 (Forstner et al, 2014), with Segemehl version 0.1.7 (Otto et al, 2014). Reads were mapped against the human genome (build GRCh37). Analysis of differential gene expression was performed with DESeq 1.18.0 (Anders and Huber, 2010). The demultiplexed FASTQ files and gene-wise quantifications have been deposited in NCBI’s Gene Expression Omnibus and are accessible through GEO Series accession numbers GSE53280 (transcriptomics of mock and *Salmonella*-infected cells) and GSE69442 (transcriptomics of HeLa cells transfected with control miRNA and miR-744-5p).

### Statistical/data analysis

Unless otherwise indicated, data are presented as mean or mean ± standard error of the mean (s.e.m.), with the exact number of independent experiments performed indicated in figure legends. Microsoft Excel (Microsoft 365) was used to compile the data, and Prism software (v10.2.1; GraphPad) was used to generate the graphs and perform statistical analysis. The normal distribution of the data was assessed by the Shapiro–Wilk test. The following tests were used for statistical comparison of datasets: for two conditions, two-tailed Student’s t-test or multiple t-test corrected for multiple comparison using the Holm-Sidak method (for parametric data), or Mann–Whitney U-test (for non-parametric data); for three or more conditions, one-way ANOVA with Dunnett’s post hoc tests (for parametric data). Values of *P* < 0.05 were considered significant. Statistical analyses are detailed in Table EV2. Violin plots were generated in Prism software; white lines show the medians, black lines indicate the 25th–75th percentiles, polygons extend to extreme values. Adobe Photoshop (v27.2; Adobe) and Adobe Illustrator (v30.1.; Adobe) were used for assembling microscopy images and figures, respectively.

## Supplementary information

Table EV1

Table EV2

Peer Review File

Source data Fig. 1

Source data Fig. 2

Source data Fig. 3

Source data Fig. 4

Source data Fig. 5

Source data Fig. 6

Expanded View Figures

## Data Availability

For the transcriptomics datasets, the demultiplexed FASTQ files and gene-wise quantifications have been deposited in NCBI’s Gene Expression Omnibus and are accessible through GEO Series accession numbers GSE53280 (transcriptomics of mock and *Salmonella*-infected cells) and GSE69442 (transcriptomics of HeLa cells transfected with control miRNA and miR-744-5p). The source data of this paper are collected in the following database record: biostudies:S-SCDT-10_1038-S44318-026-00929-3.
