# Supplementary material for: The miR-744-5p/connexin 43 axis modulates Salmonella-containing vacuole association with the Golgi network
Source: EMBO J. 2026 Oct 10;45(21):7515–40. doi: 10.1038/s44318-026-00929-3 (PMC13728154; doi:10.1038/s44318-026-00929-3)
Supplement: Supplementary file 1 [file 44318_2026_929_MOESM10_ESM.pdf]

Expanded View Figures

Figure EV1. Cx43 is a direct target of miR-744-5p.

(**A**) Quantification of intracellular *Salmonella* by CFU of HeLa cells transfected with control or miR-744-5p mimics, and infected with *Salmonella* and collected at 1, 4, 8 and 20 hpi. Results are shown as fold-change normalised to 1 hpi. (**B–D**) Representative fluorescence microscopy images (**B**) and quantification of intracellular *Salmonella* D23580 by CFU (**C**, **D**) of HeLa cells transfected with control or miR-744-5p mimics, and infected with *Salmonella* D23580, analysed at 1 and 20 hpi. CFU results in panel (**D**) are shown as fold-change normalised to 1 hpi. (**E**) Schematic representation of Cx43 3′-UTR and 3′-UTR fragments and mutant constructs used for the miRNA binding site reporter assays. The Cx43 3′-UTR was cloned downstream of Renilla luciferase in a dual-luciferase reporter plasmid (psiCHECK-2). (**F**) Results of the reporter assays using the Cx43 3′-UTR, fragments and mutant constructs. Results in cells treated with the miR-744-5p mimic are shown compared to the control miRNA. (**G**) Sequence alignment between miR-744-5p and the binding site in the 3′-UTR of human Cx43 sequence. Scale bar, 50 µm. Results are shown as mean ± s.e.m. of $n = 6$ (**A**) or $n = 5$ (**C**, **D**, **F**) biologically independent experiments; microscopy images are representative of $n = 3$ (**B**) biologically independent experiments; $^{**}P < 0.01$, and $^{***}P < 0.001$ (statistical analysis is detailed in Table EV2).

© The Author(s)

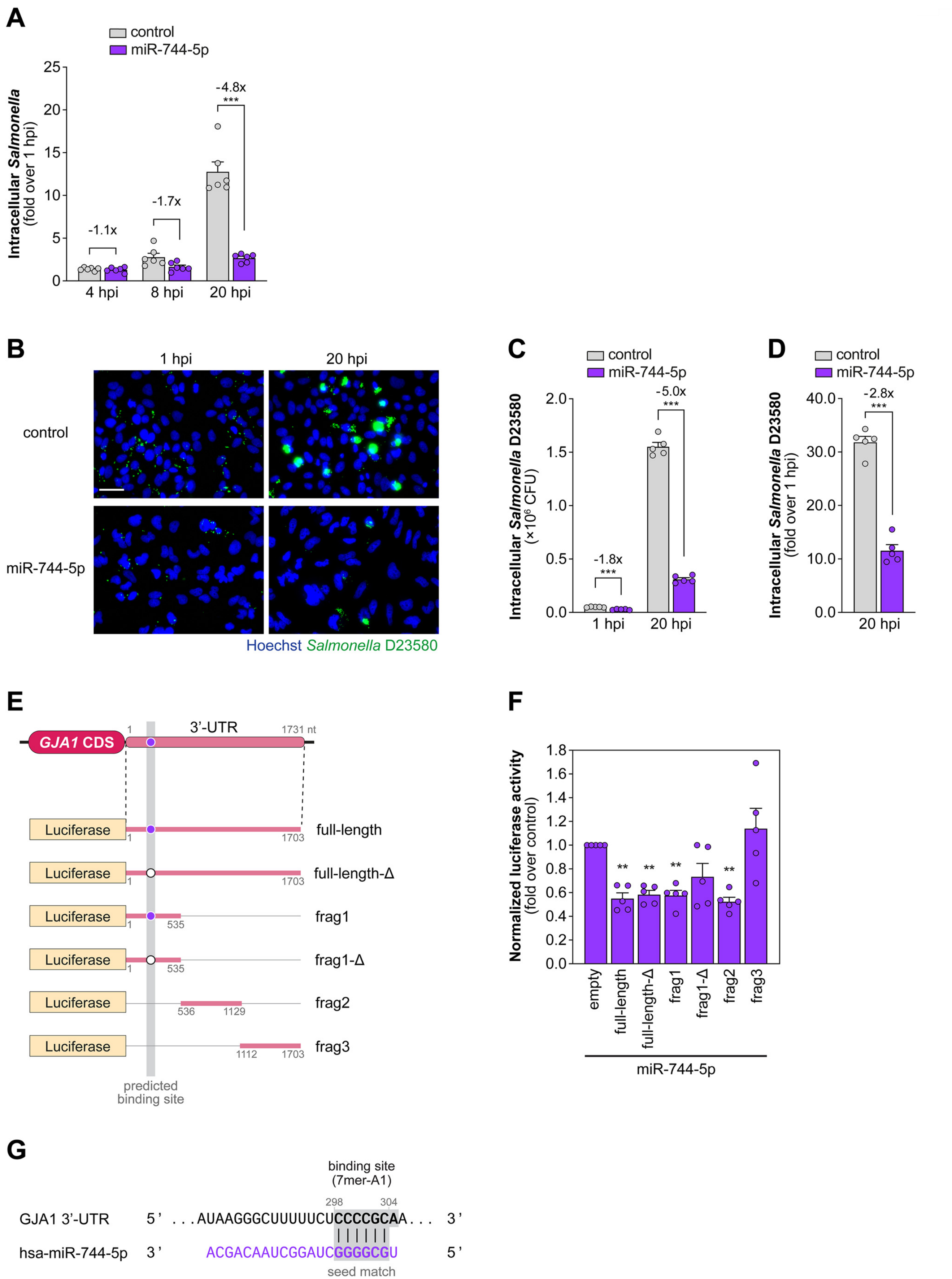
A
control
miR-744-5p
Intracellular Salmonella (fold over 1 hpi)
-1.1x
-1.7x
-4.8x
***
4 hpi
8 hpi
20 hpi
B
1 hpi
20 hpi
control
miR-744-5p
Hoechst Salmonella D23580
C
control
miR-744-5p
Intracellular Salmonella D23580 (×10⁶ CFU)
-1.8x
***
-5.0x
***
1 hpi
20 hpi
D
control
miR-744-5p
Intracellular Salmonella D23580 (fold over 1 hpi)
-2.8x
***
20 hpi
E
GJA1 CDS
3'-UTR
1
1731 nt
Luciferase
full-length
full-length-Δ
frag1
frag1-Δ
frag2
frag3
1703
535
536
1129
1112
predicted binding site
F
Normalized luciferase activity (fold over control)
empty
full-length
full-length-Δ
frag1
frag1-Δ
frag2
frag3
miR-744-5p
G
binding site (7mer-A1)
298
304
GJA1 3'-UTR 5' . . . AUAAGGGCUUUUUCUCCCCGCAA . . . 3'
hsa-miR-744-5p 3' ACGACAAUCGGAUCGGGGCGU 5'
seed match

 © The Author(s)

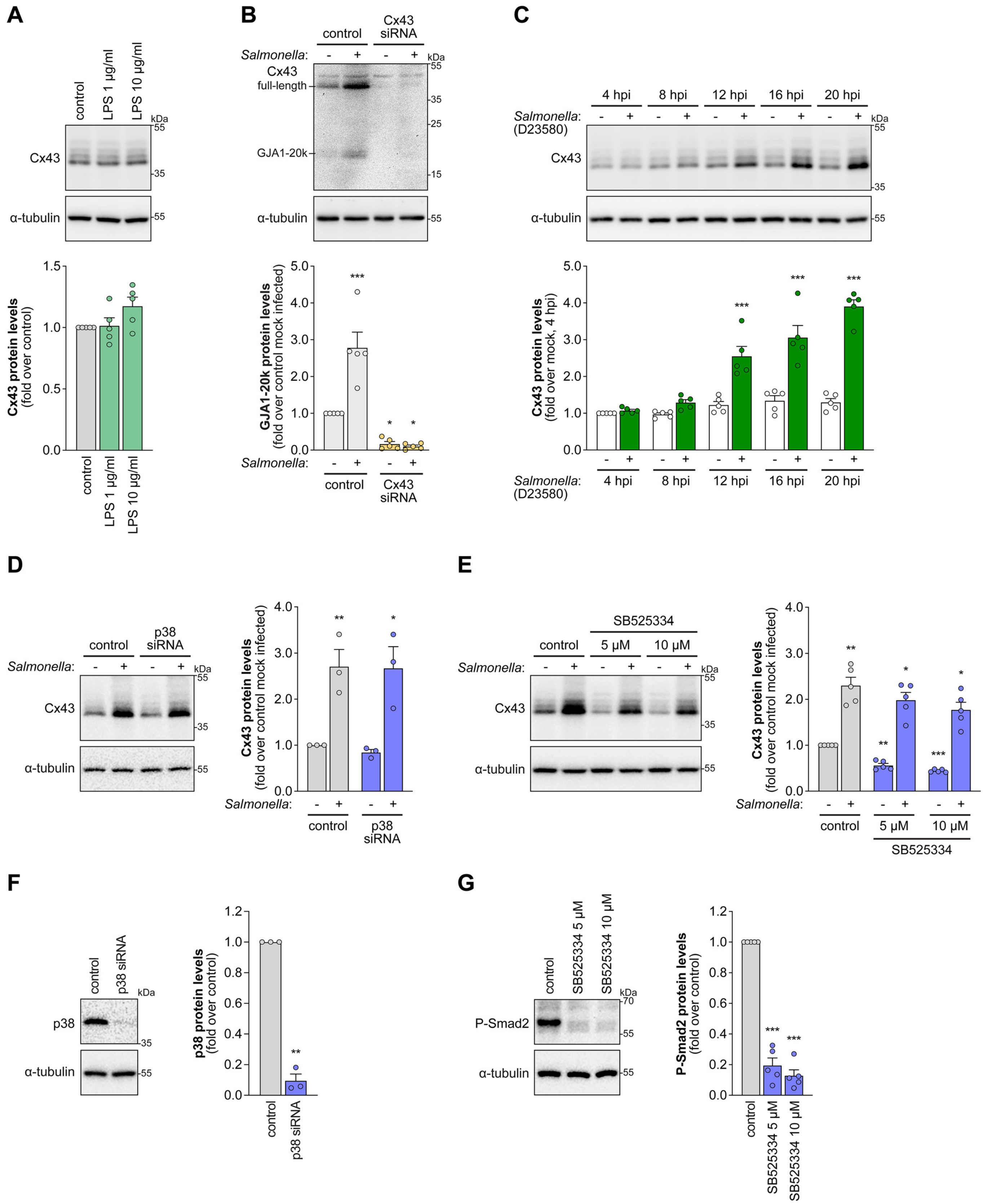
A
control
LPS 1 µg/ml
LPS 10 µg/ml
kDa
Cx43
α-tubulin
Cx43 protein levels
(fold over control)
B
control
Cx43 siRNA
Salmonella:
Cx43 full-length
GJA1-20k
α-tubulin
GJA1-20k protein levels
(fold over control mock infected)
C
4 hpi
8 hpi
12 hpi
16 hpi
20 hpi
Salmonella: (D23580)
Cx43
α-tubulin
Cx43 protein levels
(fold over mock, 4 hpi)
D
control
p38 siRNA
Salmonella:
Cx43
α-tubulin
Cx43 protein levels
(fold over control mock infected)
E
SB525334
control
5 µM
10 µM
Salmonella:
Cx43
α-tubulin
Cx43 protein levels
(fold over control mock infected)
F
control
p38 siRNA
p38
α-tubulin
p38 protein levels
(fold over control)
G
control
SB525334 5 µM
SB525334 10 µM
P-Smad2
α-tubulin
P-Smad2 protein levels
(fold over control)

© The Author(s)

Figure EV2. Cx43 is upregulated during *Salmonella* infection independently of p38 MAPK and TGF-β pathways.

(**A**) Cx43 protein levels, determined by western blot, upon treatment with purified *Salmonella* LPS, for 24 h. (**B**) Cx43 protein levels, including the ~20 kDa Cx43 isoform (GJA1-20k), determined by western blot, in HeLa cells transfected with control or Cx43 siRNAs, and either mock-treated or infected with *Salmonella*, analysed at 20 hpi. (**C**) Cx43 protein levels, determined by western blot, in HeLa cells infected with *Salmonella* D23580 (MOI 100), analysed at 4, 8, 12, 16 and 20 hpi. (**D**) Cx43 protein levels, determined by western blot, in HeLa cells transfected with control or p38 siRNAs, and either mock-treated or infected with *Salmonella*, analysed at 20 hpi. (**E**) Cx43 protein levels, determined by western blot, in HeLa cells treated with the SB525334, a selective inhibitor of the TGF-β1 receptor, or vehicle, and either infected with *Salmonella* (20 hpi) or mock-treated. (**F**) p38 protein levels, determined by western blot, in HeLa cells transfected with control or p38 siRNAs. (**G**) Smad2 phosphorylation, determined by western blot, in HeLa cells treated with vehicle or SB525334. Results are shown as mean ± s.e.m. of $n = 3$ (**D**, **F**) or $n = 5$ (**A**, **B**, **C**, **E**, **G**) biologically independent experiments; *$P < 0.05$, **$P < 0.01$, and ***$P < 0.001$ (statistical analysis is detailed in Table EV2).

 © The Author(s)

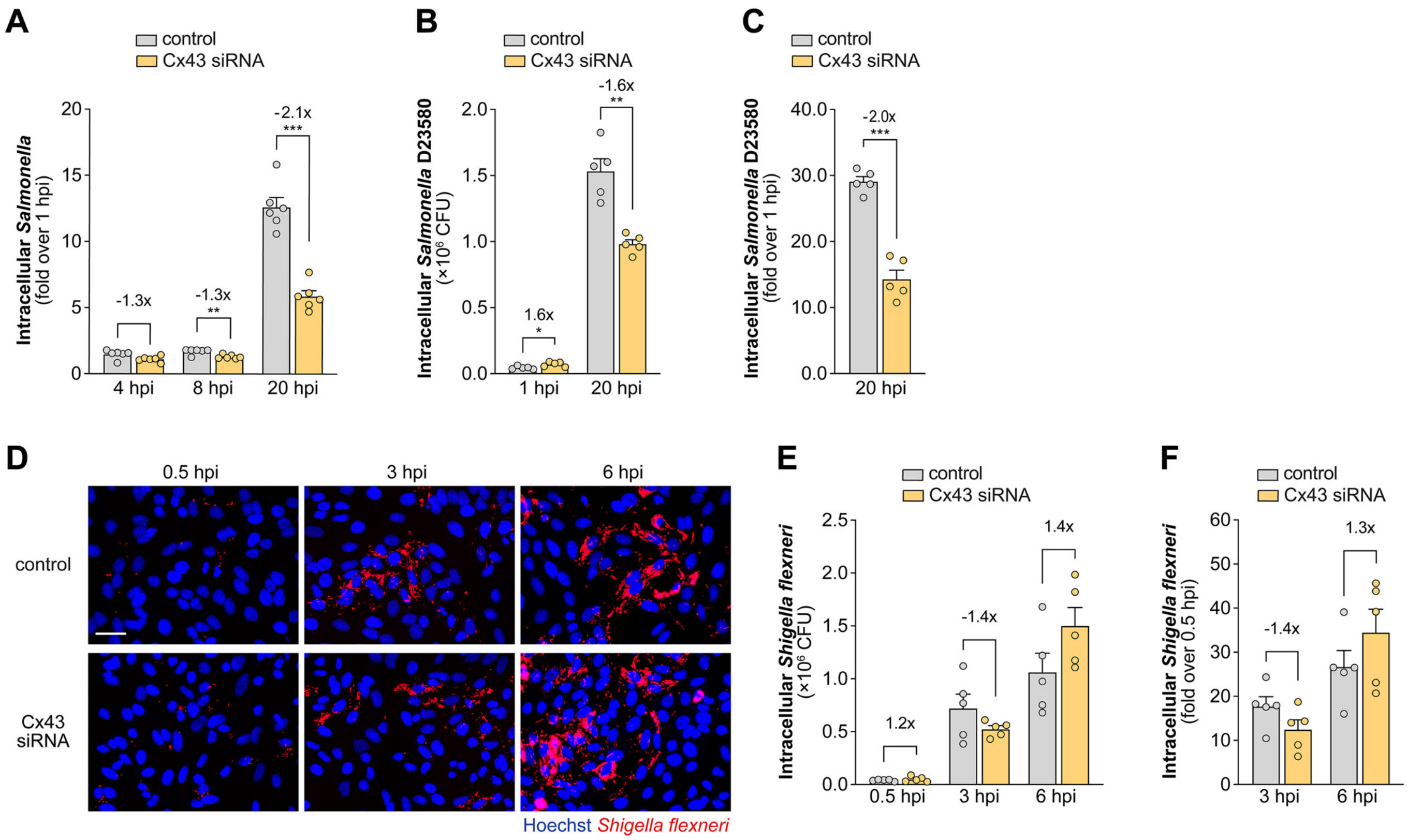

Figure EV3. Cx43 knockdown does not inhibit *Shigella* infection.

(**A**) Quantification of intracellular *Salmonella* by CFU of HeLa cells transfected with control or Cx43 siRNAs, and infected with *Salmonella*, analysed at 1, 4, 8 and 20 hpi. Results are shown as fold-change compared to 1 hpi. (**B**, **C**) Quantification of intracellular *Salmonella* D23580 by CFU of HeLa cells transfected with control or Cx43 siRNAs, and infected with *Salmonella*, analysed at 1 and 20 hpi. CFU results in (**C**) are shown as fold-change normalised to 1 hpi. (**D–F**) Representative fluorescence microscopy images (**D**) and quantification of intracellular *Shigella flexneri* by CFU (**E**, **F**) of HeLa cells transfected with control or Cx43 siRNAs, and infected with *Shigella*, analysed at 0.5, 3, and 6 hpi. CFU results in (**F**) are shown as fold-change normalised to 0.5 hpi. Scale bar, 50 µm. Results are shown as mean ± s.e.m. of $n = 6$ (**A**) or $n = 5$ (**B**, **C**, **E**, **F**) biologically independent experiments; microscopy images are representative of $n = 3$ (**D**) biologically independent experiments; $^{**}P < 0.01$, and $^{***}P < 0.001$ (statistical analysis is detailed in Table EV2).

© The Author(s)

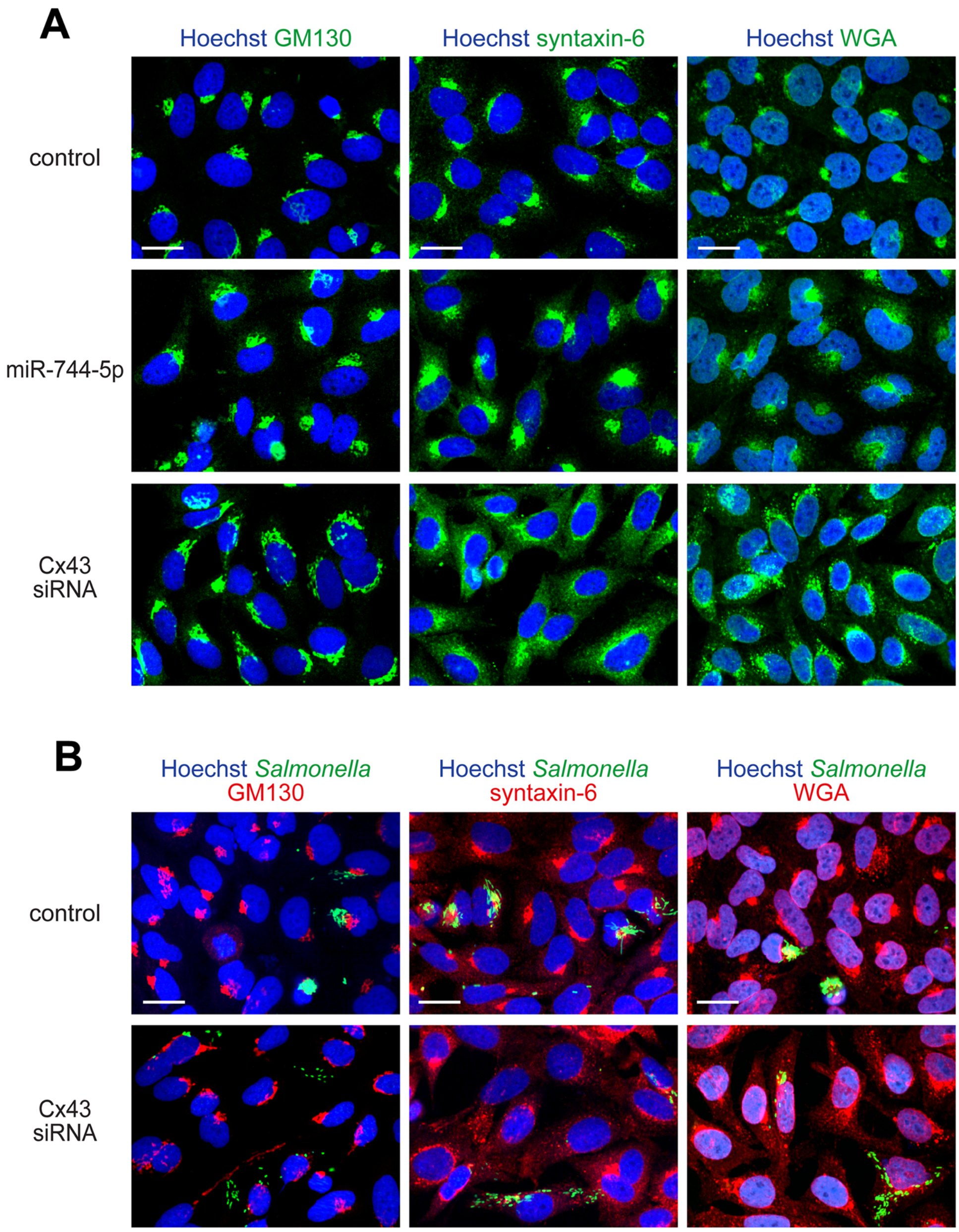

Figure EV4. Cx43 depletion disturbs Golgi network organisation and SCV positioning during Salmonella infection.

(**A**) Representative confocal fluorescence microscopy images of HeLa cells transfected with control, miR-744-5p mimics or Cx43 siRNA and stained for GM130, syntaxin-6 or WGA. (**B**) Representative confocal fluorescence microscopy images of HeLa cells transfected with control or Cx43 siRNAs, and infected with *Salmonella*, analysed at 16 hpi. Cells were stained for GM130, syntaxin-6 or WGA. Scale bar, 25 µm. Microscopy images are representative of $n = 2$ biologically independent experiments.

 © The Author(s)

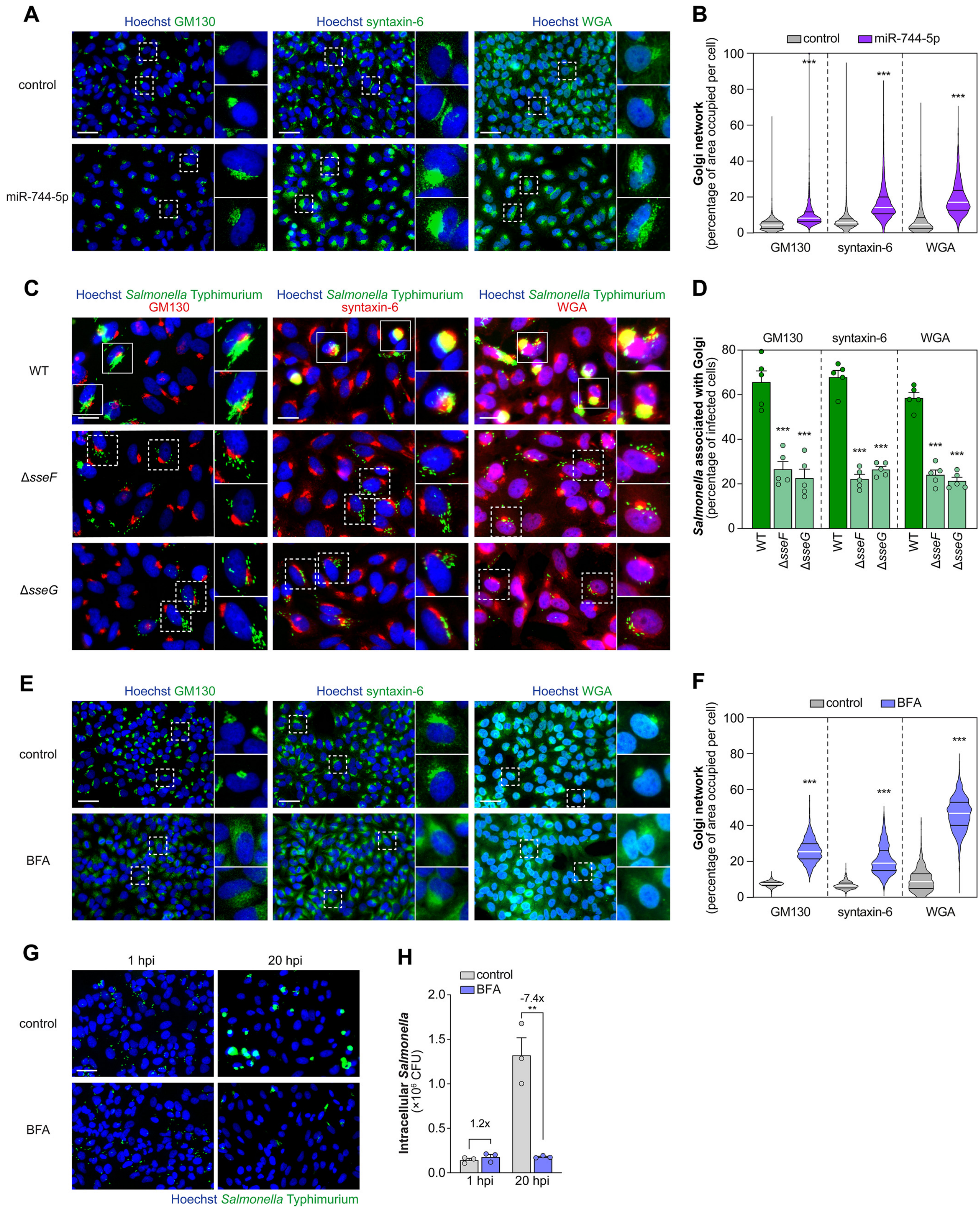
A
Hoechst GM130
Hoechst syntaxin-6
Hoechst WGA
control
miR-744-5p
B
control
miR-744-5p
Golgi network (percentage of area occupied per cell)
GM130
syntaxin-6
WGA
C
Hoechst Salmonella Typhimurium GM130
Hoechst Salmonella Typhimurium syntaxin-6
Hoechst Salmonella Typhimurium WGA
WT
ΔsseF
ΔsseG
D
Salmonella associated with Golgi (percentage of infected cells)
E
BFA
F
G
1 hpi
20 hpi
Hoechst Salmonella Typhimurium
H
Intracellular Salmonella (×10^6 CFU)
1.2x
-7.4x

Figure EV5. *Salmonella* Δ*sseF* and Δ*sseG* mutants present reduced SCV-Golgi association.

(**A**, **B**) Representative images (**A**) and quantification of the area per cell occupied by the Golgi network (**B**) in HeLa cells transfected with control or miR-744-5p mimics and stained for various Golgi markers, specifically GM130, syntaxin-6 or WGA. Results are shown from >400 cells per condition and $n = 7$ independent experiments; white lines show the medians, black lines indicate the 25th and 75th percentiles, polygons extend to extreme values. (**C**, **D**) Representative images (**C**) and quantification of *Salmonella*-infected cells in which the SCV is associated with the Golgi network (**D**) of HeLa cells infected with wild-type (WT) *Salmonella*, or Δ*sseF* and Δ*sseG* mutants (MOI 50, analysed at 16 hpi). Cells were stained for GM130, syntaxin-6 or WGA. Examples of cells classified as positive or negative regarding SCV-Golgi association are highlighted in solid and dashed lines, respectively. (**E**, **F**) Representative images (**E**) and quantification of the area per cell occupied by the Golgi network (**F**) of HeLa cells treated with BFA or vehicle, and stained for the Golgi markers GM130, syntaxin-6 or WGA. Results are shown from >500 cells per condition and independent experiment ($n = 5$); white lines show the medians, black lines indicate the 25th and 75th percentiles, polygons extend to extreme values. (**G**, **H**) Representative fluorescence microscopy images (**G**) and quantification of intracellular bacteria by CFU (**H**) of HeLa cells infected with *Salmonella*, and treated with BFA or control, analysed at 1 and 20 hpi. Scale bar, 50 µm (**A**, **E**, **G**) or 25 µm (**C**). Results are shown as mean ± s.e.m. of $n = 3$ (**H**) or $n = 5$ (**D**) biologically independent experiments; microscopy images are representative of $n = 3$ (**G**), $n = 5$ (**C**, **E**) or $n = 7$ (**A**) biologically independent experiments; $^{**}P < 0.01$, and $^{***}P < 0.001$ (statistical analysis is detailed in Table EV2).

© The Author(s)
