# Supplementary material for: The miR-744-5p/connexin 43 axis modulates Salmonella-containing vacuole association with the Golgi network
Source: EMBO J. 2026 Oct 10;45(21):7515–40. doi: 10.1038/s44318-026-00929-3 (PMC13728154; doi:10.1038/s44318-026-00929-3)
Supplement: Supplementary file 2 [file 44318_2026_929_MOESM3_ESM.pdf]

THE EMBO JOURNAL

The miR-744-5p/connexin 43 axis modulates *Salmonella*-containing vacuole association with the Golgi network

Jane Dias, Caio Franco, Laura Maria Alcantara, Susana Costa, Miguel Mano, and Ana Eulalio

Corresponding author(s): Ana Eulalio (a.eulalio@imperial.ac.uk)

Review Timeline:

Submission Date:	24th Jan 26
Editorial Decision:	11th Mar 26
Revision Received:	21st Jul 26
Editorial Decision:	18th Aug 26
Revision Received:	15th Sep 26
Accepted:	22nd Sep 26

Editor: Ieva Gailite

Transaction Report:

(Note: With the exception of the correction of typographical or spelling errors that could be a source of ambiguity, letters and reports are not edited. Depending on transfer agreements, referee reports obtained elsewhere may or may not be included in this compilation. Referee reports are anonymous unless the Referee chooses to sign their reports.)

Dear Ana,

Thank you for submitting your manuscript for consideration by the EMBO Journal. I sincerely apologise for the unusually extended assessment process of your manuscript due to delays in reviewer report submission. We have now received comments from three reviewers, which are included below for your information.

As you will see, all reviewers are generally positive in their evaluation and appreciate the contribution of the study to the research field. However, they also indicate a number of substantive concerns that would need to be addressed in order to consider publication here. In particular, they indicate that (1) the causal role of mir-744-5p-dependent Cx43 repression in regulation of Salmonella replication would need to be strengthened (reviewers #1 and #2); (2) the mechanistic link between of Cx43 and Golgi morphology regulation would need clarification (all reviewers); (3) the relevance of the identified Cx43-dependent regulation of Golgi for other Golgi-targeting pathogens would need to be tested (reviewer #1).

Based on the interest expressed in the reports, I would like to invite you to submit a revised manuscript in response to the comments by all reviewers and especially focusing on the first two main concerns outlined above. If some of the mechanistic concerns cannot be addressed, EMBO Reports could be a more suitable venue for the revised version. I would be happy to discuss the revision and the feasibility of specific experiments in more detail via email or phone/videoconferencing.

We generally allow three months as standard revision time, which can be extended to six months in the case of major revisions. Should you foresee a problem in meeting this deadline, please let us know in advance to discuss an extension.

As a matter of policy, competing manuscripts published during this period will not negatively impact on our assessment of the conceptual advance presented by your study. However, please contact me as soon as possible upon publication of any related work to discuss the appropriate course of action.

When preparing your letter of response to the referees' comments, please bear in mind that this will form part of the Review Process File and will therefore be available online to the community. For more details on our Transparent Editorial Process, please review our Editorial Policies: https://link.springer.com/partners/embo-press/editorial-policies. Please also see the attached instructions for further guidelines on preparation of the revised manuscript.

Please feel free to contact me if you have any further questions regarding the revision. Thank you for the opportunity to consider your work for publication. I look forward to discussing your revision.

With best wishes,

Ieva

Ieva Gailite, PhD
Senior Scientific Editor
The EMBO Journal
Meyerhofstrasse 1
D-69117 Heidelberg
Tel: +4962218891309
i.gailite@embojournal.org

Instructions for preparing your revised manuscript:

Read our guidance for manuscript revisions and related editorial policies: https://link.springer.com/journal/44318/submission-guidelines#cms-Revised-submissions

Please make sure you upload a letter of response to the referees' comments together with the revised manuscript.

Please also check that the title and abstract of the manuscript are brief, yet explicit, even to non-specialists.

When assembling figures, please refer to our figure preparation guideline in order to ensure proper formatting and readability in print as well as on screen:
https://media.springernature.com/original/springer-cms/rest/v1/content/27825798/data/v1

At EMBO Press we ask authors to provide source data for the main manuscript figures. You will receive a separate email with

instructions for providing source data with your revised manuscript, including how to upload and organize the files.

IMPORTANT: When you send the revision we will require
- a point-by-point response to the referees' comments, with a detailed description of the changes made (as a word file).
- a word file of the manuscript text.
- individual production quality figure files (one file per figure)
- a complete author checklist
- Expanded View files (replacing Supplementary Information)
- a Reagents and Tools Table as part of the Methods section

Please remember: Digital image enhancement is acceptable practice, as long as it accurately represents the original data and conforms to community standards. If a figure has been subjected to significant electronic manipulation, this must be noted in the figure legend or in the 'Materials and Methods' section. The editors reserve the right to request original versions of figures and the original images that were used to assemble the figure.

We realize that it is difficult to revise to a specific deadline. In the interest of protecting the conceptual advance provided by the work, we recommend a revision within 3 months (9th Jun 2026). Please discuss the revision progress ahead of this time with the editor if you require more time to complete the revisions. Use the link below to submit your revision:

https://emboj.msubmit.net/cgi-bin/main.plex

Referee #1:

In this manuscript, Dias and colleagues uncover a microRNA-mediated regulatory axis in mammalian cells that is controlled by the intracellular bacterial pathogen Salmonella enterica subsp. Typhimurium (STm) to promote its intracellular replication in vacuoles associated with the Golgi membrane network. Based on a previous study showing that STm infection downregulates the microRNA, mir-744-5p, the authors now show that mir-744-5p specifically impairs STm replication and identify Connexin 43 (Cx43) as a functional target of mir-744-5p, which is upregulated during STm infection of HeLa cells and required for STm replication. Interestingly, they further show that Cx43 promotes STm replication via an unexpected role in maintenance of Golgi organization, distinct from its role in gap junctions. Taken altogether, the authors propose a model in which STm downregulates mir-744-5p during infection, which in turns causes upregulation of Cx43, whose role in Golgi homeostasis ensures optimal vacuolar replication of STm.

This is an interesting study that provides a mechanistic understanding of how microRNAs contribute to host-pathogen interactions and uncover an unsuspected role of Connexin 43 in regulating Golgi morphology and functions that benefit STm. The study is well-designed and the authors' conclusions are supported by the data, which is clearly presented. While the role of Connexin 43 in supporting SCV association with the Golgi network and STm vacuolar replication is clearly established, the mir-744-5p/Cx43 axis is only implied by association but not directly demonstrated in the context of an STm infection. Additional data further supporting the new role of Connexin 43 in Golgi organization and whether it contributes to replication of other Golgi-interacting pathogens would also solidify the impact of this study.

Major comments:

1. The authors propose that the mir-744-5p/Cx43 axis contributes to STm replication based on converging evidence that mir-744-5p represses Cx43 expression while STm infection downregulates mir-744-5p expression and increases Cx43 expression. To better establish this functional relationship, the authors should consider testing whether overexpression of mir-744-5p in infected cells reverses Cx43 increased production, already knowing that it impairs replication. In these conditions, is the Golgi network disrupted as in Cx43-depleted cells?

2. The authors have used Shigella flexneri as a control pathogen in their study to establish the specificity of mir-744-5p towards modulating STm replication, which is an important aspect of the study. As a cytosolic pathogen, Shigella does not inhabit an intracellular niche comparable to Salmonella, nor does it interact with the Golgi network. This raises the question of whether Connexin 43's role in Golgi homeostasis also contributes to replication of other Golgi-targeting pathogens, such as Chlamydia for example.

3. The new role of Cx43 in Golgi homeostasis is intriguing but not documented in this study beyond the effect of Cx43 depletion on Golgi morphology, leaving the possibility that it may be indirect. Could the authors further substantiate this important finding by for example examining whether Cx43 is associated with Golgi membranes?

Minor Comments to address:

1. Fluorescence microscopy images presented in Fig. 6A, 6C, S6A and S6C are of poor resolution (and with saturated signals) with respect to clearly showing Golgi morphology changes and SCV association with the Golgi apparatus. I would recommend that the authors collect new images using a higher resolution microscope (deconvolution or confocal) to provide better quality data.

2. Could the authors clarify how they quantified SCV association with the Golgi apparatus? Was it performed manually or via automatic image analysis and what criteria were used for categorization? Previous studies have used MTOC-SCV distance as unbiased measurements, but it is unclear how the authors analyzed their images.

3. Line 176: "ssaG" should be italicized.

4. Line 192: "impair" should be replaced with "determine" for the sentence to reflect the meaning intended.

Minor Comments to consider:

In the model presented in Fig. 6, the use of arrows of various thickness may be confusing, as one could read that the inhibition of Connexin 43 expression by mir-744-5p promotes bacterial replication, while it is the opposite due to the upstream down regulation of mir-774-5p by Salmonella via E2F1. Perhaps emphasizing the Salmonella-driven regulatory events in bold would better highlight how modulation of the mir-744-5p/Cx43 axis contributes to bacterial replication?

Referee #2:

During infection the pathogenic bacteria Salmonella has many strategies to modify the host cell, enabling its intracellular replication. Key to this is the ability of Salmonella to modify the Salmonella-containing vacuole to create a permissive replicative niche through its association with the Golgi. The mechanism by which Salmonella does this is unclear. In this paper Dias et al. describe a new host-pathogen interaction that is involved in this essential element of Salmonella infection biology. They describe how an miRNA, previously shown to be downregulated during Salmonella infection, controls the expression of Connexin 43, which is important for Golgi organisation and therefore intracellular Salmonella replication. Although the authors do not attempt to determine the molecular mechanism by which Connexin 43 mediates this, their clear identification of its role is of general interest to cellular microbiologists. It also has interesting implications for how miRNAs regulate gene expression during host-pathogen interactions.

This paper is very well written and clear. The data is consistently excellently presented and convincing. It is well quantified and controlled, with plenty of repeats. I only have a few minor comments.

Minor Comments
1. The authors have previously demonstrated that Salmonella infection leads to a reduction in miR-744-5p. Here they show that Salmonella infection causes an increase in Cx43 expression. They clearly demonstrate that overexpression of miR-744-5p leads to a reduction in Cx43 expression. However, they have not actually demonstrated that the increase in Cx43 expression during infection is specifically due to the reduction in miR-744-5p levels. To prove a direct link, it would be necessary to infect cells that are transfected with miR-744-5p mimics.

2. The authors go on to suggest that the increase in Cx43 is not caused by p38 MAPK or TGF-beta pathways. The p38 siRNA has no effect on Cx43 levels in uninfected cells, so this is not an informative experiment. This set of data does not rule out that Cx43 upregulation could be due to another response during infection. Without the experiment suggested in comment 1 the statement on page 6 lines 137-140 should be toned down.

3. The authors do not attempt to explain the mechanism of Cx43 action. However, the paper would be strengthened with some attempt to characterise Cx43 during infection, for example does its localisation change during infection?

4. A better description of the image analysis pipelines would be useful. A better measure for the quantification of SPI-2 activation would be the percentage of bacteria with SPI-2 activation, rather than the percentage of cells infected with SPI-2 activated bacteria.

Referee #3:

In this manuscript the authors identified miR-744-5p as a miRNA that impairs Salmonella infection. A major target of miR-744-5p is the gap junction protein connexin43 (Cx43), which the authors found was upregulated by Salmonella. By directly manipulating Cx43 expression, using Cx43 siRNA and Cx43 cDNA, the authors confirmed the relationship between Cx43 expression and

Salmonella propagation. The authors show data suggesting that Cx43 has a non-cannonical role in regulating the architecture of the Golgi apparatus into a conformation that facilitates the formation of Salmonella-containing vacuoles (SCVs) that are Golgi associated. The evidence implicating Cx43 in formation of SCVs is robust although the the study stops short of identifying the precise mechanism by which Cx43 influences Golgi morphology to promote SCV formation.

Specific comments

A significant omission is that the intracellular distribution of Cx43 was not analyzed. This is important, since the data suggests that the role of Cx43 in promoting SCV formation is distinct from gap junctional communication which occurs through plasma membrane channels. Does Salmonella increase the Golgi localization of Cx43?

A role for Cx43 in organizing the cytoskeleton is proposed as a potential mechanism for Cx43 to alter Golgi morphology/SCV formation. This could be directly analyzed by examining the effect of Cx43 expression/repression on actin and microtubule organization.

The immunoblots are cropped at 35 kDa. Several lines of evidence support a role for a 20 kDa isoform of Cx43, GJA1-20k, in regulating cytoskeletal organization. Is that present in uncropped immunoblots for Cx43? If not, noting that GJA1-20k is not present should be included in the text.

The miR-744-5p/Connexin 43 Axis Modulates *Salmonella*-Containing Vacuole Association with the Golgi Network
EMBOJ-2026-123671R

We are very grateful to the Reviewers for their supportive evaluation of our work and constructive comments. We greatly appreciate the insightful suggestions, which have helped us to substantially improve the manuscript.

In response to the comments, we performed several additional experiments that considerably strengthen the mechanistic conclusions of the study. Specifically, we now demonstrate that the infection-induced upregulation of Cx43 is directly mediated by the downregulation of miR-744-5p, thereby establishing a causal link between the miR-744-5p/Cx43 regulatory axis and *Salmonella* infection. We further show that miR-744-5p overexpression disrupts Golgi organization, reinforcing the role of this regulatory axis in maintaining the Golgi architecture required for efficient intracellular bacterial replication. In addition, we demonstrate that Cx43 redistributes to the Golgi region during infection and overexpression, supporting a non-canonical intracellular role for Cx43 in regulating Golgi organization. We also show that modulation of Cx43 expression does not induce major alterations in the overall organization of the actin or microtubule cytoskeleton, suggesting that the observed effects on Golgi organization are unlikely to result from large-scale cytoskeletal remodelling. Furthermore, we identify the ~20 kDa Cx43 isoform, GJA1-20k, as being upregulated during *Salmonella* infection and discuss its potential contribution to the observed phenotypes. Finally, we demonstrate that Cx43 overexpression does not affect *Shigella flexneri* replication, highlighting the distinct mechanisms by which intracellular pathogens exploit Golgi biology. We have also acquired higher-resolution microscopy images, expanded the description of our image acquisition and analysis procedures, and revised the manuscript throughout to address all of the Reviewers comments.

We provide below a detailed description of how we have addressed the specific comments of the Reviewers.

Reviewers Comments: black
Our reply: blue

Referee #1:
In this manuscript, Dias and colleagues uncover a microRNA-mediated regulatory axis in mammalian cells that is controlled by the intracellular bacterial pathogen Salmonella enterica subsp. Typhimurium (STm) to promote its intracellular replication in vacuoles associated with the Golgi membrane network. Based on a previous study showing that STm infection downregulates the microRNA, mir-744-5p, the authors now show that mir-744-5p specifically impairs STm replication and identify Connexin 43 (Cx43) as a functional target of mir-744-5p, which is upregulated during STm infection of HeLa cells and required for STm replication. Interestingly, they further show that Cx43 promotes STm replication via an unexpected role in maintenance of Golgi organization, distinct from its role in gap junctions. Taken altogether, the authors propose a model in which

STm downregulates mir-744-5p during infection, which in turns causes upregulation of Cx43, whose role in Golgi homeostasis ensures optimal vacuolar replication of STm.

This is an interesting study that provides a mechanistic understanding of how microRNAs contribute to host-pathogen interactions and uncover an unsuspected role of Connexin 43 in regulating Golgi morphology and functions that benefit STm. The study is well-designed and the authors' conclusions are supported by the data, which is clearly presented. While the role of Connexin 43 in supporting SCV association with the Golgi network and STm vacuolar replication is clearly established, the mir-744-5p/Cx43 axis is only implied by association but not directly demonstrated in the context of an STm infection. Additional data further supporting the new role of Connexin 43 in Golgi organization and whether it contributes to replication of other Golgi-interacting pathogens would also solidify the impact of this study.

Major comments:

1. The authors propose that the mir-744-5p/Cx43 axis contributes to STm replication based on converging evidence that mir-744-5p represses Cx43 expression while STm infection downregulates mir-744-5p expression and increases Cx43 expression. To better establish this functional relationship, the authors should consider testing whether overexpression of mir-744-5p in infected cells reverses Cx43 increased production, already knowing that it impairs replication. In these conditions, is the Golgi network disrupted as in Cx43-depleted cells?

To address this point, we performed the suggested experiment by transfecting HeLa cells with miR-744-5p mimics prior to *Salmonella* infection followed by analysis of Cx43 expression. We found that overexpression of miR-744-5p prevented the infection-induced increase in Cx43 protein levels, demonstrating that Cx43 upregulation is dependent on the reduction of miR-744-5p observed during infection. Moreover, it reinforces a causal/mechanistic link between Cx43 upregulation and *Salmonella* replication. These data were included in **Figure 3e** of the revised manuscript.

Furthermore, we examined Golgi morphology and observed that overexpression of miR-744-5p disrupted Golgi organization, although this effect was not as strong as that observed upon Cx43 depletion by siRNA treatment. This is most likely related with the stronger reduction of Cx43 protein levels induced by siRNA treatment, compared to miR-744-5p (**Fig. 2c**). These data have been included in **Figure EV5a,b** of the revised manuscript.

Together, these results provide direct evidence that the miR-744-5p/Cx43 axis regulates Golgi homeostasis during infection and further support our proposed model linking this pathway to intracellular bacterial replication.

2. The authors have used Shigella flexneri as a control pathogen in their study to establish the specificity of mir-744-5p towards modulating STm replication, which is an important aspect of the study. As a cytosolic pathogen, Shigella does not inhabit an intracellular

niche comparable to Salmonella, nor does it interact with the Golgi network. This raises the question of whether Connexin 43's role in Golgi homeostasis also contributes to replication of other Golgi-targeting pathogens, such as Chlamydia for example.

As noted by the Reviewer, extending our findings to additional pathogens would be valuable. However, unlike *Salmonella*, many bacterial pathogens, including *Chlamydia trachomatis, Legionella pneumophila, Rickettsia rickettsii, Streptococcus pyogenes, Streptococcus pneumoniae, Anaplasma phagocytophilum* (Aistleitner *et al.*, 2020; Heuer *et al.*, 2009; Klabunde *et al.*, 2023; Liu *et al.*, 2021; Nozawa *et al.*, 2021; Read *et al.*, 2024), exploit Golgi fragmentation rather than Golgi integrity during infection.

To further explore the broader relevance of the Cx43–Golgi axis in bacterial infection, we revisited *Shigella flexneri*, which we initially used as a control pathogen. As pointed by the Reviewer, *Shigella* replicates in the cytosol, but it has a profound effect on the Golgi. In agreement with previous studies (Burnaevskiy *et al.*, 2013; Mounier *et al.*, 2012), we confirmed that *Shigella* infection induces marked disruption of the Golgi apparatus under our experimental conditions.
We then investigated whether increasing Cx43 expression could influence *Shigella* intracellular replication. In contrast to *Salmonella*, Cx43 overexpression did not significantly affect *Shigella* replication.

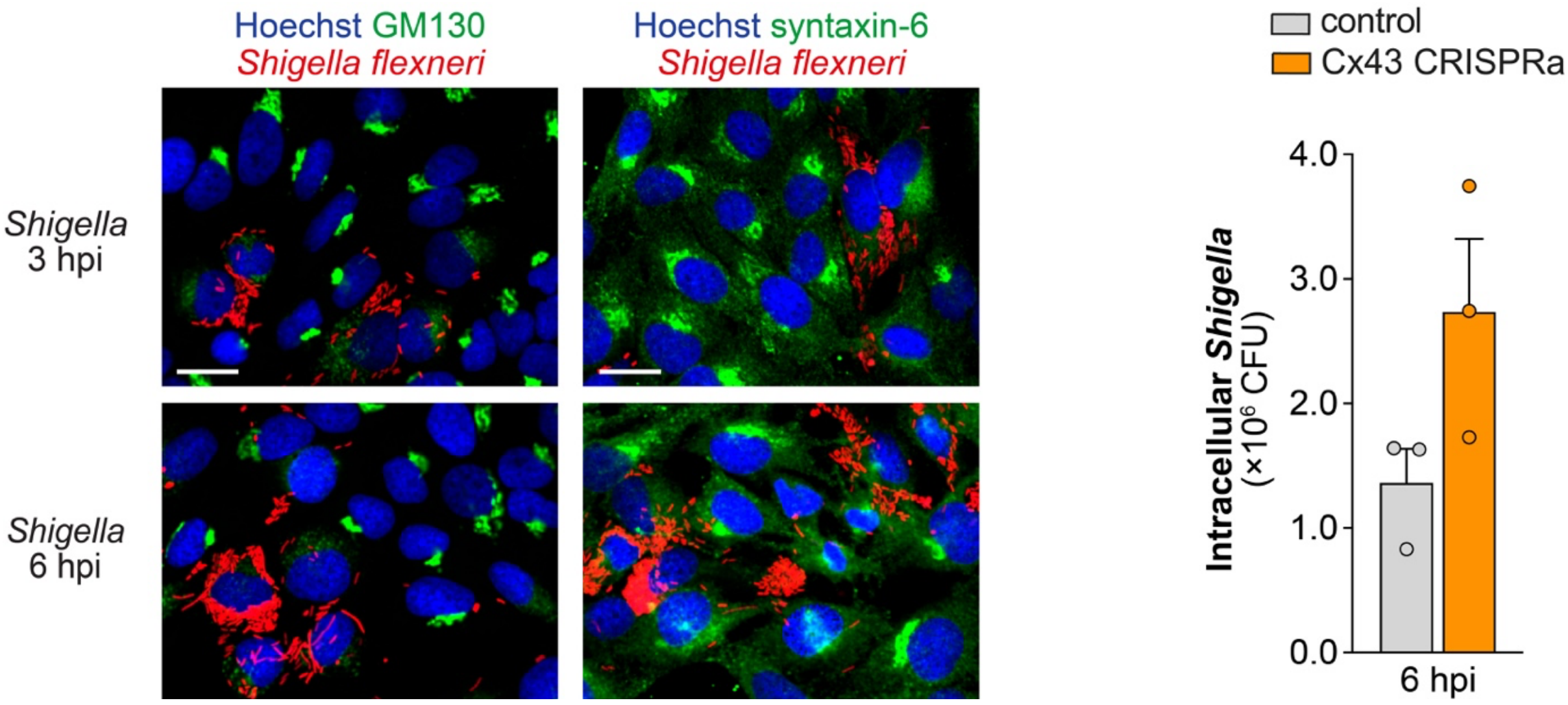

Although this result indicates that the Cx43-dependent effect on Golgi integrity is not sufficient to alter *Shigella* infection, it is consistent with the distinct mechanisms by which these pathogens manipulate the Golgi. Unlike *Salmonella*, which benefits from maintaining an intact Golgi-associated replicative niche, *Shigella* actively induces Golgi fragmentation through the action of the T3SSeffector IpaB, which promotes cholesterol redistribution and disrupts Golgi structure and the recycling network (Mounier *et al.*, 2012). This mechanism is likely dominant over any stabilizing effect derived from increased Cx43 expression, explaining why Cx43 overexpression does not rescue Golgi organization sufficiently to impair *Shigella* replication.

We have expanded the Discussion to better distinguish the fundamentally different strategies used by intracellular pathogens to manipulate Golgi homeostasis (**lines 270-287**), specifically adding that *Shigella* infection disrupts the Golgi. We also note that extending these studies to other Golgi-interacting pathogens, such as *Chlamydia* or *Rickettsia*, would be of considerable interest but lies beyond the scope of the present study.

3. The new role of Cx43 in Golgi homeostasis is intriguing but not documented in this study beyond the effect of Cx43 depletion on Golgi morphology, leaving the possibility that it may be indirect. Could the authors further substantiate this important finding by for example examining whether Cx43 is associated with Golgi membranes?

To address this point, we examined the intracellular localization of Cx43 during *Salmonella* infection and following CRISPRa-mediated Cx43 overexpression. These analyses revealed increased localization of Cx43 in proximity with the Golgi region during infection. This redistribution is consistent with a role for Cx43 in regulating Golgi homeostasis independently of its canonical localisation at plasma membrane gap junctions. These findings substantially strengthen the proposed mechanism linking Cx43 to maintenance of Golgi organization during infection.
These data are presented in **Figure 6e,f** and **lines 229-234** of the revised manuscript.

Minor Comments to address:

1. Fluorescence microscopy images presented in Fig. 6A, 6C, S6A and S6C are of poor resolution (and with saturated signals) with respect to clearly showing Golgi morphology changes and SCV association with the Golgi apparatus. I would recommend that the authors collect new images using a higher resolution microscope (deconvolution or confocal) to provide better quality data.

We have acquired confocal microscopy images for the Cx43 localization during *Salmonella* infection (**Fig. 6f**) and following CRISPRa-mediated Cx43 overexpression (**Fig. 6e**), as well as for the cytoskeleton organization upon modulation of Cx43 expression (**Fig. 4h,i**; see point 2 of Reviewer 3).

For the experiments presented in **Figures 6a and 6c**, we retained the original widefield microscopy images because these were used for the quantitative analyses presented in the manuscript (**Fig. 6b, 6d**), ensuring consistency between the representative images and the quantified dataset. However, to address the Reviewer's concern, we have performed additional independent experiments and acquired the images using confocal microscopy under the same experimental conditions. As shown below, these higher-resolution images recapitulate the original observations and clearly support our conclusion that Cx43 depletion disrupts Golgi organization and reduces the association of SCVs with the Golgi apparatus. These images were not included in the manuscript to avoid duplication, but we will do so if the Reviewer deems it necessary.

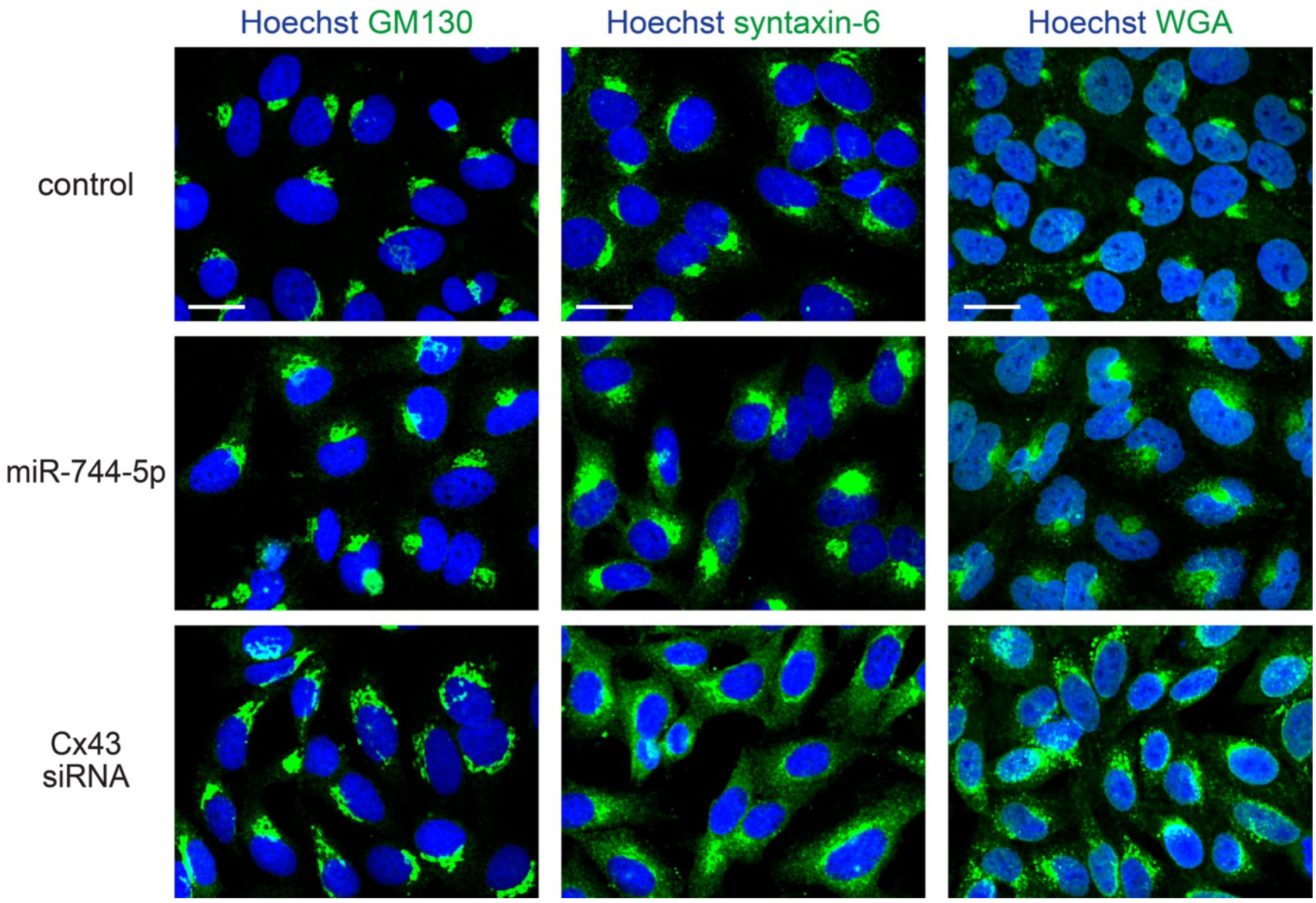

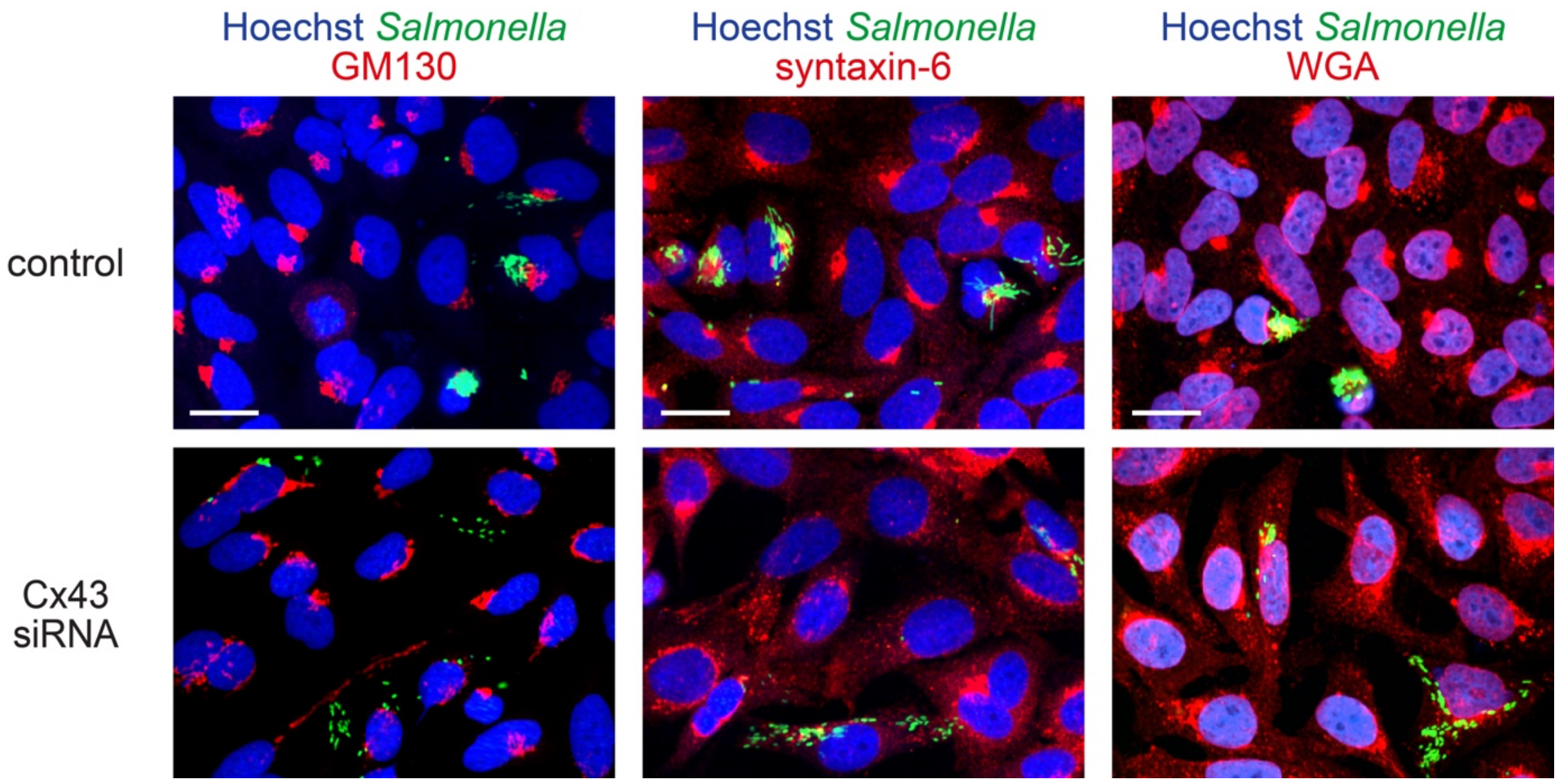

2. Could the authors clarify how they quantified SCV association with the Golgi apparatus? Was it performed manually or via automatic image analysis and what criteria were used for categorization? Previous studies have used MTOC-SCV distance as unbiased measurements, but it is unclear how the authors analyzed their images.

We have rewritten and substantially expanded the Image Acquisition and Analysis section of the Methods to clarify the concerns raised by the Reviewer and provide additional detail.
Specifically regarding the Golgi–SCV association quantification, we now describe that this analysis was performed manually using the ImageJ manual classifier, and we clarify the criteria used for classification. Briefly, infected cells were scored as positive when the majority of SCVs were either superimposed or in close proximity to the Golgi apparatus, whereas cells were scored as negative when SCVs were distributed throughout the cytoplasm, even if a subset of bacteria was located near the Golgi. We also clarify that the classification reflects the predominant intracellular distribution of SCVs on a per-cell basis, rather than the position of individual bacteria, and we specify the exclusion criteria applied to ensure robust and unbiased scoring. To illustrate this classification criteria, examples of cells classified as positive or negative regarding SCV-Golgi association are now highlighted (solid and dashed lines, respectively) in **Fig. 6c, EV5c**. We believe these additions clarify our analysis strategy and the rationale behind the categorization.
These details have been added to the Methods (**lines 478–487**).

3. Line 176: "ssaG" should be italicized.

Corrected.

4. Line 192: "impair" should be replaced with "determine" for the sentence to reflect the meaning intended.

Corrected.

Minor Comments to consider:

In the model presented in Fig. 6, the use of arrows of various thickness may be confusing, as one could read that the inhibition of Connexin 43 expression by mir-744-5p promotes bacterial replication, while it is the opposite due to the upstream down regulation of mir-774-5p by Salmonella via E2F1. Perhaps emphasizing the Salmonella-driven regulatory events in bold would better highlight how modulation of the mir-744-5p/Cx43 axis contributes to bacterial replication?

We appreciate this suggestion and have introduced changes to the schematic model to better emphasize the *Salmonella*-driven regulatory events. The revised figure now more clearly illustrates that *Salmonella*-mediated downregulation of miR-744-5p results in increased Cx43 expression, maintenance of Golgi integrity and enhanced intracellular replication.

Referee #2:

During infection the pathogenic bacteria Salmonella has many strategies to modify the host cell, enabling its intracellular replication. Key to this is the ability of Salmonella to modify the Salmonella-containing vacuole to create a permissive replicative niche through its association with the Golgi. The mechanism by which Salmonella does this is unclear. In this paper Dias et al. describe a new host-pathogen interaction that is involved in this essential element of Salmonella infection biology. They describe how an miRNA, previously shown to be downregulated during Salmonella infection, controls the expression of Connexin 43, which is important for Golgi organisation and therefore intracellular Salmonella replication. Although the authors do not attempt to determine the molecular mechanism by which Connexin 43 mediates this, their clear identification of its role is of general interest to cellular microbiologists. It also has interesting implications for how miRNAs regulate gene expression during host-pathogen interactions.

This paper is very well written and clear. The data is consistently excellently presented and convincing. It is well quantified and controlled, with plenty of repeats. I only have a few minor comments.

Minor Comments

1. The authors have previously demonstrated that Salmonella infection leads to a reduction in miR-744-5p. Here they show that Salmonella infection causes an increase in Cx43 expression. They clearly demonstrate that overexpression of miR-744-5p leads to a reduction in Cx43 expression. However, they have not actually demonstrated that the increase in Cx43 expression during infection is specifically due to the reduction in miR-744-5p levels. To prove a direct link, it would be necessary to infect cells that are transfected with miR-744-5p mimics.

We fully agree with the Reviewer's comment and have now performed these experiments (see also point 1 of Reviewer 1).
Overexpression of miR-744-5p prevented infection-induced Cx43 upregulation, providing evidence that the increase in Cx43 during infection results from reduced miR-744-5p levels. Moreover, it reinforces the causal link between the Cx43 increase and *Salmonella* replication. These data have been included in **Figure 3e** of the revised manuscript.

2. The authors go on to suggest that the increase in Cx43 is not caused by p38 MAPK or TGF-beta pathways. The p38 siRNA has no effect on Cx43 levels in uninfected cells, so this is not an informative experiment. This set of data does not rule out that Cx43 upregulation could be due to another response during infection. Without the experiment suggested in comment 1 the statement on page 6 lines 137-140 should be toned down.

We agree with the Reviewer that while our data do not support a major contribution of p38 MAPK or TGF-β signalling to Cx43 upregulation under our experimental conditions, they do not exclude the involvement of additional infection-induced pathways.

However, the experiment suggested in comment 1 (shown in **Figure 3e** of the revised manuscript), demonstrated that miR-744-5p overexpression prevented the infection-induced increase in Cx43 protein levels, supporting our model that downregulation of miR-744-5p is critical for Cx43 upregulation under our experimental conditions.

Considering these new data, we have revised the corresponding section of the manuscript to clarify that our results support a central role for the miR-744-5p/Cx43 regulatory axis during *Salmonella* infection (**lines 146-149**). At the same time, we acknowledge that we cannot exclude the possibility that additional infection-induced signalling pathways may also contribute to regulating Cx43 expression (**lines 141-145**).

3. The authors do not attempt to explain the mechanism of Cx43 action. However, the paper would be strengthened with some attempt to characterise Cx43 during infection, for example does its localisation change during infection?

We have analysed Cx43 intracellular localization during infection and following CRISPRa-mediated overexpression (see also point 3 of Reviewer 1). These experiments revealed increased localization of Cx43 within the Golgi region in both experimental conditions, supporting a non-canonical intracellular role for Cx43.
These results are presented in **Figure 6e,f** of the revised manuscript.

4. A better description of the image analysis pipelines would be useful. A better measure for the quantification of SPI-2 activation would be the percentage of bacteria with SPI-2 activation, rather than the percentage of cells infected with SPI-2 activated bacteria.

The Image Acquisition and Analysis section of the Methods has been rewritten and substantially expanded to provide a more detailed description of all image analysis procedures (**lines 441-490**).

Regarding SPI-2 activation, we appreciate the Reviewer's suggestion and have also included the quantification of the percentage of bacteria expressing the SPI-2 reporter in addition to the percentage of infected cells containing SPI-2-positive bacteria. The results were comparable, showing that also the percentage of bacteria expressing the SPI-2 reporter were similar in control and Cx43 knockdown cells.
These results are presented in **Figure 5c (right panel)** of the revised manuscript.

Referee #3:
In this manuscript the authors identified miR-744-5p as a miRNA that impairs Salmonella infection. A major target of miR-744-5p is the gap junction protein

connexin43 (Cx43), which the authors found was upregulated by Salmonella. By directly manipulating Cx43 expression, using Cx43 siRNA and Cx43 cDNA, the authors confirmed the relationship between Cx43 expression and Salmonella propagation. The authors show data suggesting that Cx43 has a non-cannonical role in regulating the architecture of the Golgi apparatus into a conformation that facilitates the formation of Salmonella-containing vacuoles (SCVs) that are Golgi associated. The evidence implicating Cx43 in formation of SCVs is robust although the the study stops short of identifying the precise mechanism by which Cx43 influences Golgi morphology to promote SCV formation.

Specific comments

A significant omission is that the intracellular distribution of Cx43 was not analyzed. This is important, since the data suggests that the role of Cx43 in promoting SCV formation is distinct from gap junctional communication which occurs through plasma membrane channels. Does Salmonella increase the Golgi localization of Cx43?

We agree that this is an important question (see also point 3 of Reviewer 1 and point 3 of Reviewer 2).
We therefore examined Cx43 localization during *Salmonella* infection and observed increased localization of Cx43 in the Golgi region, supporting the proposed intracellular role of Cx43 in regulating Golgi homeostasis independently of gap junction communication.
These data are presented in **Figure 6e,f** of the revised manuscript.

A role for Cx43 in organizing the cytoskeleton is proposed as a potential mechanism for Cx43 to alter Golgi morphology/SCV formation. This could be directly analyzed by examining the effect of Cx43 expression/repression on actin and microtubule organization.

Under our experimental conditions, we did not observe dramatic alterations in the overall organization of either the actin or microtubule cytoskeleton upon Cx43 knockdown using siRNAs or overexpression using CRISPRa. These findings suggest that the effects of Cx43 on Golgi morphology are unlikely to result from large-scale cytoskeletal remodelling. However, we cannot exclude the possibility that Cx43 influences more subtle or localized cytoskeletal dynamics that are not readily detected by conventional fluorescence microscopy but may nevertheless contribute to Golgi organization and SCV positioning.
We have included these new data in **Figure 4h,i** and **lines 171-176** of the revised manuscript.

The immunoblots are cropped at 35 kDa. Several lines of evidence support a role for a 20 kDa isoform of Cx43, GJA1-20k, in regulating cytoskeletal organization. Is that present in uncropped immunoblots for Cx43? If not, noting that GJA1-20k is not present should be included in the text.

In our original experiments, the immunoblot conditions were optimized to detect full-length Cx43, with the proteins being resolved on a 10% polyacrylamide gel.

In response to the Reviewer's suggestion, we performed new immunoblot analyses using gel conditions optimized to resolve lower molecular weight proteins (12.5% polyacrylamide gel). Under these conditions, we detected a band at approximately 20 kDa that is specific for Cx43, as its signal was abolished following Cx43 siRNA-mediated knockdown. Interestingly, we also found that this 20 kDa isoform is upregulated during *Salmonella* infection, similarly to full-length Cx43. These new data have been included in **Figure EV2b** of the revised manuscript. All uncropped immunoblots of the manuscript are provided in **Source Data** and **Appendix Figure S1**.

While the precise contribution of GJA1-20k to the phenotypes described in this study remains to be determined, this Cx43 isoform has been implicated in regulating cellular processes, including cytoskeletal organization (Basheer *et al.*, 2017; Baum *et al.*, 2025). However, we did not observe major changes in cytoskeletal organization upon Cx43 depletion or overexpression in our experimental system (**Figure 4h,i; see point above**), indicating that additional/alternative mechanisms contribute to the effects of Cx43 on Golgi organization during *Salmonella* infection.

We have introduced a section in the Discussion highlighting GJA1-20k as a potential contributor and noting that defining the specific functions of the different Cx43 isoforms during infection will be an important avenue for future investigation (**lines 317-325**).

Cited references:

Aistleitner K, Clark T, Dooley C, Hackstadt T (2020) Selective fragmentation of the trans-Golgi apparatus by Rickettsia rickettsii. *PLoS Pathog* 16: e1008582

Basheer WA, Xiao S, Epifantseva I, Fu Y, Kleber AG, Hong T, Shaw RM (2017) GJA1-20k Arranges Actin to Guide Cx43 Delivery to Cardiac Intercalated Discs. *Circ Res* 121: 1069–1080

Baum R, Nguyen VD, Maalouf M, Shimura D, Waghalter M, Srapyan S, Jin Q, Kuzmanovich L, Gaffney AT, Bell BR *et al* (2025) A truncated isoform of Connexin43 caps actin to organize forward delivery of full-length Connexin43. *J Cell Biol* 224

Burnaevskiy N, Fox TG, Plymire DA, Ertelt JM, Weigele BA, Selyunin AS, Way SS, Patrie SM, Alto NM (2013) Proteolytic elimination of N-myristoyl modifications by the Shigella virulence factor IpaJ. *Nature* 496: 106–109

Heuer D, Rejman Lipinski A, Machuy N, Karlas A, Wehrens A, Siedler F, Brinkmann V, Meyer TF (2009) Chlamydia causes fragmentation of the Golgi compartment to ensure reproduction. *Nature* 457: 731–735

Klabunde B, Wesener A, Bertrams W, Ringshandl S, Halder LD, Vollmeister E, Schmeck B, Benedikter BJ (2023) Streptococcus pneumoniae disrupts the structure of the golgi apparatus and subsequent epithelial cytokine response in an H(2)O(2)-dependent manner. *Cell Commun Signal* 21: 208

Liu Y, Mukherjee R, Bonn F, Colby T, Matic I, Glogger M, Heilemann M, Dikic I (2021) Serine-ubiquitination regulates Golgi morphology and the secretory pathway upon Legionella infection. *Cell Death Differ* 28: 2957–2969

Mounier J, Boncompain G, Senerovic L, Lagache T, Chretien F, Perez F, Kolbe M, Olivo-Marin JC, Sansonetti PJ, Sauvonnet N (2012) Shigella effector IpaB-induced cholesterol relocation disrupts the Golgi complex and recycling network to inhibit host cell secretion. *Cell Host Microbe* 12: 381–389

Nozawa T, Iibushi J, Toh H, Minowa-Nozawa A, Murase K, Aikawa C, Nakagawa I (2021) Intracellular Group A Streptococcus Induces Golgi Fragmentation To Impair Host Defenses through Streptolysin O and NAD-Glycohydrolase. *mBio* 12

Read CB, Ali AN, Stephenson DJ, Macknight HP, Maus KD, Cockburn CL, Kim M, Xie X, Carlyon JA, Chalfant CE (2024) Ceramide-1-phosphate is a regulator of Golgi structure and is co-opted by the obligate intracellular bacterial pathogen Anaplasma phagocytophilum. *mBio* 15: e0029924

1st Revision - Editorial Decision 18th Aug 2026

Dear Ana,

Thank you for submitting a revised version of your manuscript to The EMBO journal. Your study has now been seen by two of the original referees, who find that their previous concerns have been sufficiently addressed and now recommend publication of the manuscript after minor revisions.

Based on the remaining reviewer comments, please perform the quantification of Cx43 colocalisation with the perinuclear Golgi as requested by reviewer #2. In response to the comment by reviewer #1, please include the high-resolution images in the Expanded View figures.

In addition to the remaining minor reviewer requests, there are a few editorial points that need to be addressed before I can extend the official acceptance of the manuscript:

1. Please note that it is The EMBO Journal policy for the transcript of the editorial process (containing referee reports and your response letters) to be published as an online supplement to each paper. If you should prefer removal of any referee-only figures included in the point-by-point response(s), e.g. because they may still be used for future publication or because they have been reproduced from published work by others, please do let us know immediately via response email.
More information is available here: https://www.embopress.org/transparent-process#Review_Process
2. Please enter all funding information into the line-by-line list in our system. Please note that this funding information will be linked in PubMed and will not be included in the proofs. Any information entered into the Comments field cannot be linked, so please ensure that the line-by-line list is complete and accurate.
3. Please update the order and the headings of the manuscript sections to: Abstract / Introduction / Results / Discussion / Methods / Data Availability / Acknowledgements / Disclosure and Competing Interests Statement / References / Figure Legends / Expanded View Figure Legends.
4. CRediT has replaced the traditional author contributions section because it offers a systematic, machine-readable author contributions format that allows for more effective research assessment. Please remove the Authors Contributions from the manuscript and use the free text boxes beneath each contributing author's name in our online submission system to add specific details on the author's contribution. More information is available in our guide to authors.
5. Please add a "Disclosure and Competing Interests Statement" section after "Acknowledgements".
6. Please check the order of callouts: currently, Table EV2 is called out before Table EV1.
7. Please remove Appendix Figure S1, since source data are uploaded in the folders corresponding to the individual figures.
8. Papers published in The EMBO Journal are accompanied online by a 'Synopsis' to enhance discoverability of the manuscript. It consists of A) a short (1-2 sentences) summary of the findings and their significance, B) 3-4 bullet points highlighting key results (the highlights currently included in the manuscript can be used here) and C) a synopsis image that is 550x300 pixels large (width x height, jpeg or png format). You can either show a model or key data in the synopsis image. Please note that the image size is rather small and that text needs to be readable at the final size.

Please let me know if you have any questions regarding any of these points. You can use the link below to upload the revised files.

Thank you again for giving us the chance to consider your manuscript for The EMBO Journal. I look forward to receiving the final version.

With best wishes,

Ieva

Ieva Gailite, PhD
Senior Scientific Editor
The EMBO Journal
Meyerhofstrasse 1
D-69117 Heidelberg
Tel: +4962218891309
i.gailite@embojournal.org

We realize that it is difficult to revise to a specific deadline. In the interest of protecting the conceptual advance provided by the work, we recommend a revision within 3 months (16th Nov 2026). Please discuss the revision progress ahead of this time with the editor if you require more time to complete the revisions. Use the link below to submit your revision:

https://emboj.msubmit.net/cgi-bin/main.plex

Referee #1:

In this revised version of their manuscript, Dias and colleagues have thoroughly addressed all my original concerns with additional experimental work that has consolidated the study and strengthened their conclusions. In particular, they further establish the functional connection between miR-744-5p and Connexin 43 expression and its contribution to optimal SCV association with the Golgi apparatus during Salmonella infection. They also clarify the non-canonical function of Connexin 43 in maintaining Golgi structures.
My only remaining minor comment regards the inclusion of the confocal images presented in the authors' responses to the reviewers' comments. As expected, these images provide higher resolution of the intracellular structures (Golgi and SCVs) than their original images and increase the quality of the data presented and I would recommend that they are used to replace the original images of Figure 6C. since they reproduce the results generated originally. However, I appreciate the point of presenting images that were used for the quantitative analysis presented in Figure 6D, so I will leave it to the editors to make their recommendation on whether representative images need to come from experimental repeats used for quantitative analysis or not.

Referee #2:

The authors have engaged well with the reviewer comments. Additional experiments strengthen their conclusions. However, one additional experiment is not well analysed. All three reviewers asked the authors to assess whether Cx43 was relocalised to the Golgi during infection. The authors have addressed this in a new figure 6e and f. They state that "Cx43 showed increased accumulation in the perinuclear Golgi region". However, without single colour images or quantification of this phenotype this is impossible to confirm. The only thing that is clear is that there is a brighter Cx43 signal when overexpressed. This could be addressed by quantifying the proportion of the total cellular Cx43 signal at the Golgi region, and comparing between 1) control and CRISPRa, and 2) uninfected and infected cells.

The miR-744-5p/Connexin 43 Axis Modulates *Salmonella*-Containing Vacuole Association with the Golgi Network
EMBOJ-2026-123671R1

We provide below a detailed description of how we have addressed the remaining comments of the Reviewers.

Reviewers Comments: black
Our reply: blue

Referee #1:
In this revised version of their manuscript, Dias and colleagues have thoroughly addressed all my original concerns with additional experimental work that has consolidated the study and strengthened their conclusions. In particular, they further establish the functional connection between miR-744-5p and Connexin 43 expression and its contribution to optimal SCV association with the Golgi apparatus during Salmonella infection. They also clarify the non-canonical function of Connexin 43 in maintaining Golgi structures.

My only remaining minor comment regards the inclusion of the confocal images presented in the authors' responses to the reviewers' comments. As expected, these images provide higher resolution of the intracellular structures (Golgi and SCVs) than their original images and increase the quality of the data presented and I would recommend that they are used to replace the original images of Figure 6C. since they reproduce the results generated originally. However, I appreciate the point of presenting images that were used for the quantitative analysis presented in Figure 6D, so I will leave it to the editors to make their recommendation on whether representative images need to come from experimental repeats used for quantitative analysis or not.

We thank the Reviewer for the positive assessment of our revised manuscript and for this helpful suggestion.

Following the Editor's recommendation, we have now included the high-resolution confocal microscopy images as an Expanded View figure (**Fig. EV4A,B**). These images provide improved visualization of the Golgi network and SCVs and recapitulate the observations obtained with the original microscopy approach used for quantification (**Fig. 6A,C**).

To accommodate the journal's limit of five Expanded View figures, we have also consolidated the previous Figures EV2 and EV3 into a single figure (**Fig. EV2**), as these experiments address the related topic of Cx43 expression during *Salmonella* infection and the mechanisms contributing to its upregulation.

Referee #2:

The authors have engaged well with the reviewer comments. Additional experiments strengthen their conclusions. However, one additional experiment is not well analysed. All three reviewers asked the authors to assess whether Cx43 was relocalised to the Golgi during infection. The authors have addressed this in a new figure 6e and f. They state that "Cx43 showed increased accumulation in the perinuclear Golgi region". However, without single colour images or quantification of this phenotype this is impossible to confirm. The only thing that is clear is that there is a brighter Cx43 signal when overexpressed. This could be addressed by quantifying the proportion of the total cellular Cx43 signal at the Golgi region, and comparing between 1) control and CRISPRa, and 2) uninfected and infected cells.

We thank the Reviewer for this suggestion and agree that quantitative analysis was required to substantiate the increased overlap of Cx43 within the Golgi region.

We have now quantified Cx43-Golgi overlap, expressed as the percentage of the Golgi area positive for Cx43 signal. This analysis revealed a highly significant increase in Cx43-Golgi overlap both following CRISPRa-mediated Cx43 overexpression and during *Salmonella* infection. These quantitative data are now included in **Figure 6F,H** of the revised manuscript and support our conclusion that Cx43 shows increased accumulation within the Golgi region during infection and following Cx43 overexpression.

As **Figure 6** has consequently increased in size, we have moved the proposed model to a separate figure (**Fig. 7**).

Dear Ana,

Thank you for incorporating the final editorial and formatting requests in the manuscript. I am now pleased to inform you that your manuscript has been accepted for publication. Congratulations on a nice study!

Before we forward your manuscript to our publishers, we would like to propose some edits in the manuscript title and synopsis. We have also selected a short blurb that will accompany the title of your manuscript in our online table of contents. Please take a look at the proposed textual changes in the attached text file - any corrections or edits from your side are very welcome.

Your manuscript will be processed for publication by EMBO Press. It will be copy edited and you will receive page proofs prior to publication. Please note that you will be contacted by Springer Nature Author Services to complete licensing and payment information.

You may qualify for financial assistance for your publication charges - either via a Springer Nature fully open access agreement or an EMBO initiative. Check your eligibility: https://link.springer.com/journal/44318/how-to-publish-with-us

Should you be planning a Press Release on your article, please notify the production team via this form in order to coordinate publication and release dates: https://forms.cloud.microsoft/e/eH4A8JVP6g

Once I have received your input on the final textual edits, I will be happy to forward your manuscript to our publishers for typesetting.

Thank you for this interesting contribution to The EMBO Journal!

Best wishes,

Ieva

Ieva Gailite, PhD
Senior Scientific Editor
The EMBO Journal
Meyerhofstrasse 1
69117 Heidelberg
i.gailite@embojournal.org

** Click here to be directed to your login page: https://emboj.msubmit.net

EMBO Press Author Checklist

Corresponding Author Name: Ana Eulalio	
Journal Submitted to: The EMBO Journal	
Manuscript Number: EMBOJ-2026-123671R1	

USEFUL LINKS FOR COMPLETING THIS FORM
The EMBO Journal - Author Guidelines
EMBO Reports - Author Guidelines
Molecular Systems Biology - Author Guidelines
EMBO Molecular Medicine - Author Guidelines

Reporting Checklist for Life Science Articles (updated January
This checklist is adapted from Materials Design Analysis Reporting (MDAR) Checklist for Authors. MDAR establishes a minimum set of requirements in transparent reporting in the life sciences (see Statement of Task: 10.31222/osf.io/9sm4x). Please follow the journal's guidelines in preparing your
Please note that a copy of this checklist will be published alongside your article.

Abridged guidelines for figures
1. Data
The data shown in figures should satisfy the following conditions:
- the data were obtained and processed according to the field's best practice and are presented to reflect the results of the experiments in an accurate and unbiased manner.
- ideally, figure panels should include only measurements that are directly comparable to each other and obtained with the same assay.
- plots include clearly labeled error bars for independent experiments and sample sizes. Unless justified, error bars should not be shown for technical
- if n<5, the individual data points from each experiment should be plotted. Any statistical test employed should be justified.
- Source Data should be included to report the data underlying figures according to the guidelines set out in the authorship guidelines on Data

2. Captions
Each figure caption should contain the following information, for each panel where they are relevant:
- a specification of the experimental system investigated (eg cell line, species name).
- the assay(s) and method(s) used to carry out the reported observations and measurements.
- an explicit mention of the biological and chemical entity(ies) that are being measured.
- an explicit mention of the biological and chemical entity(ies) that are altered/varied/perturbed in a controlled manner.
- the exact sample size (n) for each experimental group/condition, given as a number, not a range;
- a description of the sample collection allowing the reader to understand whether the samples represent technical or biological replicates (including how many animals, litters, cultures, etc.).
- a statement of how many times the experiment shown was independently replicated in the laboratory.
- definitions of statistical methods and measures:
  - common tests, such as t-test (please specify whether paired vs. unpaired), simple χ2 tests, Wilcoxon and Mann-Whitney tests, can be unambiguously identified by name only, but more complex techniques should be described in the methods section;
  - are tests one-sided or two-sided?
  - are there adjustments for multiple comparisons?
  - exact statistical test results, e.g., P values = x but not P values < x;
  - definition of 'center values' as median or average;
  - definition of error bars as s.d. or s.e.m.

Please complete ALL of the questions below.
Select "Not Applicable" only when the requested information is not relevant for your study.

Materials

Newly Created Materials	Information included in the manuscript?	In which section is the information available? (Reagents and Tools Table, Materials and Methods, Figures, Data Availability Section)
New materials and reagents need to be available; do any restrictions apply?	Not Applicable	

Antibodies	Information included in the manuscript?	In which section is the information available? (Reagents and Tools Table, Materials and Methods, Figures, Data Availability Section)
For **antibodies** provide the following information:<br>- Commercial antibodies: RRID (if possible) or supplier name, catalogue number and or/clone number<br>- Non-commercial: RRID or citation	Yes	Reagents and Tools Table, Materials and Methods

DNA and RNA sequences	Information included in the manuscript?	In which section is the information available? (Reagents and Tools Table, Materials and Methods, Figures, Data Availability Section)
**Short novel DNA or RNA including primers, probes**: provide the sequences.	Yes	Reagents and Tools Table, Materials and Methods, Table EV1

Cell materials	Information included in the manuscript?	In which section is the information available? (Reagents and Tools Table, Materials and Methods, Figures, Data Availability Section)
**Cell lines:** Provide species information, strain. Provide accession number in repository **OR** supplier name, catalog number, clone number, and/**OR** RRID.	Yes	Reagents and Tools Table, Materials and Methods
**Primary cultures:** Provide species, strain, sex of origin, genetic modification status.	Not Applicable	
Report if the cell lines were recently **authenticated** (e.g., by STR profiling) and tested for mycoplasma contamination.	Yes	Materials and Methods

Experimental animals	Information included in the manuscript?	In which section is the information available? (Reagents and Tools Table, Materials and Methods, Figures, Data Availability Section)
**Laboratory animals or Model organisms:** Provide species, strain, sex, age, genetic modification status. Provide accession number in repository **OR** supplier name, catalog number, clone number, **OR** RRID.	Not Applicable	
**Animal observed in or captured from the field:** Provide species, sex, and age where possible.	Not Applicable	
Please detail **housing and husbandry conditions**.	Not Applicable	

Plants and microbes	Information included in the manuscript?	In which section is the information available? (Reagents and Tools Table, Materials and Methods, Figures, Data Availability Section)
**Plants:** provide species and strain, ecotype and cultivar where relevant, unique accession number if available, and source (including location for collected wild specimens).	Not Applicable	
**Microbes:** provide species and strain, unique accession number if available, and source.	Yes	Reagents and Tools Table, Materials and Methods

Human research participants	Information included in the manuscript?	In which section is the information available? (Reagents and Tools Table, Materials and Methods, Figures, Data Availability Section)
If collected and within the bounds of privacy constraints report on age, sex and gender or ethnicity for all study participants.	Not Applicable	

Core facilities	Information included in the manuscript?	In which section is the information available? (Reagents and Tools Table, Materials and Methods, Figures, Data Availability Section)
If your work benefited from core facilities, was their service mentioned in the acknowledgments section?	Not Applicable	

Design

Study protocol	Information included in the manuscript?	In which section is the information available? (Reagents and Tools Table, Materials and Methods, Figures, Data Availability Section)
If study protocol has been **pre-registered, provide DOI in the manuscript**. For clinical trials, provide the trial registration number **OR** cite DOI.	Not Applicable	
Report the **clinical trial registration number** (at ClinicalTrials.gov or equivalent), where applicable.	Not Applicable	

Laboratory protocol	Information included in the manuscript?	In which section is the information available? (Reagents and Tools Table, Materials and Methods, Figures, Data Availability Section)
Provide DOI OR other citation details if **external detailed step-by-step protocols** are available.	Not Applicable	

Experimental study design and statistics	Information included in the manuscript?	In which section is the information available? (Reagents and Tools Table, Materials and Methods, Figures, Data Availability Section)
Include a statement about **sample size** estimate even if no statistical methods were used.	Not Applicable	
Were any steps taken to minimize the effects of subjective bias when allocating animals/samples to treatment (e.g. **randomization procedure**)? If yes, have they been described?	Not Applicable	
Include a statement about **blinding** even if no blinding was done.	Yes	Blinding was not performed in this study. In most cases image acquisition and analysis is automated.
Describe **inclusion/exclusion criteria** if samples or animals were excluded from the analysis. Were the criteria pre-established?<br><br>If sample or data points were omitted from analysis, report if this was due to attrition or intentional exclusion and provide justification.	Not Applicable	
For every figure, are **statistical tests** justified as appropriate? Do the data meet the assumptions of the tests (e.g., normal distribution)? Describe any methods used to assess it. Is there an estimate of variation within each group of data? Is the variance similar between the groups that are being statistically compared?	Yes	Materials and Methods, Table EV2

Sample definition and in-laboratory replication	Information included in the manuscript?	In which section is the information available? (Reagents and Tools Table, Materials and Methods, Figures, Data Availability Section)
In the figure legends: state number of times the experiment was **replicated** in laboratory.	Yes	Figure legends
In the figure legends: define whether data describe **technical or biological replicates**.	Yes	Figure legends

Ethics

Ethics	Information included in the manuscript?	In which section is the information available? (Reagents and Tools Table, Materials and Methods, Figures, Data Availability Section)
Studies involving **human participants**: State details of **authority granting ethics approval** (IRB or equivalent committee(s), provide reference number for approval.	Not Applicable	
Studies involving **human participants**: Include a statement confirming that **informed consent** was obtained from all subjects and that the experiments conformed to the principles set out in the WMA Declaration of Helsinki and the Department of Health and Human Services Belmont Report.	Not Applicable	
Studies involving **human participants:** For publication of **patient photos**, include a statement confirming that consent to publish was obtained.	Not Applicable	
Studies involving experimental **animals**: State details of **authority granting ethics approval** (IRB or equivalent committee(s), provide reference number for approval. Include a statement of compliance with ethical regulations.	Not Applicable	
Studies involving **specimen and field samples:** State if relevant **permits** obtained, provide details of authority approving study; if none were required, explain why.	Not Applicable	

Dual Use Research of Concern (DURC)	Information included in the manuscript?	In which section is the information available? (Reagents and Tools Table, Materials and Methods, Figures, Data Availability Section)
Could your study fall under dual use research restrictions? Please check biosecurity documents and list of **select agents and toxins** (CDC): https://www.selectagents.gov/sat/list.htm	Not Applicable	
If you used a select agent, is the security level of the lab appropriate and reported in the manuscript?	Not Applicable	
If a study is subject to dual use research of concern regulations, is the name of the **authority granting approval and reference number** for the regulatory approval provided in the manuscript?	Not Applicable	

Reporting

The MDAR framework recommends adoption of discipline-specific guidelines, established and endorsed through community initiatives. Journals have their own policy about requiring specific guidelines and recommendations to complement MDAR.

Adherence to community standards	Information included in the manuscript?	In which section is the information available? (Reagents and Tools Table, Materials and Methods, Figures, Data Availability Section)
State if relevant guidelines or checklists (e.g., **ICMJE, MIBBI, ARRIVE, PRISMA**) have been followed or provided.	Not Applicable	
For **tumor marker prognostic studies,** we recommend that you follow the **REMARK** reporting guidelines (see link list at top right). See author guidelines, under 'Reporting Guidelines'. Please confirm you have followed these guidelines.	Not Applicable	
For **phase II and III randomized controlled trials**, please refer to the **CONSORT** flow diagram (see link list at top right) and submit the CONSORT checklist (see link list at top right) with your submission. See author guidelines, under 'Reporting Guidelines'. Please confirm you have submitted this list.	Not Applicable	

Data Availability

Data availability	Information included in the manuscript?	In which section is the information available? (Reagents and Tools Table, Materials and Methods, Figures, Data Availability Section)
Have **primary datasets** been deposited according to the journal's guidelines (see 'Data Deposition' section) and the respective accession numbers provided in the Data Availability Section?	Yes	Materials and Methods, Data Availability section
Were **human clinical and genomic datasets** deposited in a public access-controlled repository in accordance to ethical obligations to the patients and to the applicable consent agreement?	Not Applicable	
Are **computational models** that are central and integral to a study available without restrictions in a machine-readable form? Were the relevant accession numbers or links provided?	Not Applicable	
If publicly available data were reused, provide the respective **data citations in the reference list**.	Not Applicable	
